# From Atomic Interactions to Molecular Miscibility and Philicity: Deciphering Enthalpic Driving Forces

**DOI:** 10.1021/acs.jpca.6c01567

**Published:** 2026-06-22

**Authors:** Anna Luisa Upterworth, Daniel Sebastiani

**Affiliations:** Department of Chemistry, 9176 Martin Luther University Halle-Wittenberg, 06120 Halle, Germany

## Abstract

We present a numerical analysis on the nature of the interactions that determine the philicity of a molecule based on molecular dynamics simulations. Specifically, the focus is on a binary mixture of alkanes and perfluoroalkanes representing lipophilic and fluorophilic molecules. The enthalpic factors that lead to the mixing/demixing phase behavior are subject to study, as this phase behavior constitutes the basis of the concept of philicity. A particular feature of the present study is the application of an energy decomposition scheme, which enables the elucidation of the sensitivity of effective interaction strengths between atoms of a given species to variations in the elementary parameters of the pairwise interatomic interaction potentials (in our case, Lennard-Jones potentials).

## Graphical abstract

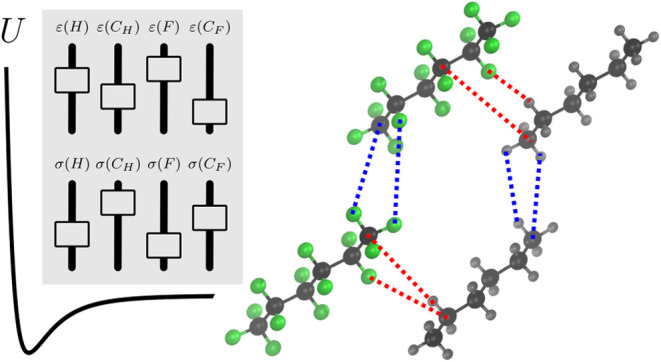

## Introduction

“Philicity” is a key concept in liquid chemistry that describes the affinity of a molecule (or moiety) toward a specific environment. Hydrophilicity refers to solvation in water, lipophilicity to solvation in fat-like solvents such as hydrocarbons, and fluorophilicity^1^ to solubility in fluorous compounds. Due to its qualitative character, philicity is typically discussed and described in a phenomenological manner in terms of miscibility with the respective reference solvent. According to the “like dissolves like” principle,^2,3^ matching philicities of two components A and B lead to mixing. There is an equilibrium of three groups of competing intermolecular interactions: the autointeractions of component A with component A and component B with component B, and the cross-interactions of component A with component B.^4^ If the difference in the components’ philicities is large, the affinity toward interactions with molecules of the same species versus the other species is high, favoring immiscibility/phase separation.^5^ However, this perspective considers only one part of the thermodynamics of mixing: the strength of the attractive intermolecular interactions solely determines the enthalpy of mixing. At the same time, temperature-dependent entropic forces drive the system toward mixing. The actual state of mixing is thus determined not only by the equilibrium of counteracting intermolecular interactions, but also by the competition between enthalpy and entropy. A special point is the aspect of the local morphology of the constituents at the atomic scale. The bare interaction potentials must always be seen in combination with the “accessibility” of the interaction, which is governed by the shape of the involved molecules and their steric compatibilities. This aspect has been shown to be highly relevant in the context of species-dependent diffusion behavior in multicomponent mixtures.^6,7^

A common approach to measuring and comparing philicity is to express it in terms of partition coefficients log *P*.^8^ Lipophilicity is generally associated with partition in organic solvent–water systems, such as 1-octanol–water.^9,10^ Similarly, Kiss et al. defined fluorophilicity in terms of partition between perfluoro­(methylcyclohexane) and toluene.^11^ However, philicity is a relative property that is influenced by many factors, e.g., temperature or pH,^10^ and can therefore not readily be represented in its full functionality by a single measurable parameter. It is also desirable to link apparent miscibility to the molecular structure via quantitative descriptors based on the elementary interactions. This idea is reflected in fragmental and group-contribution methods to predict log *P*,^9^ and elsewhere in the local electrophilicity descriptor introduced by Chattaraj et al. to compare the reactivity of specific sites.^12^ Yet, to the best of our knowledge, there is no equivalent approach to understanding macroscopic (im)­miscibility as a result of all local, site–site interactions, where each type of interaction contributes differently to the overall free energy of mixing.

To address this, we have previously conducted a comprehensive computational study on the microscopic origin of philicity on the example of a mixture of a regular alkane with a perfluorinated alkane.^13^ Such systems exhibit a strong philicity mismatch between the lipophilic alkane and the fluorophilic perfluoroalkane. They are characterized by comparably high upper critical solution temperatures.^14–17^ Although the phase behavior is dominated by van der Waals interactions,^18^ the intrinsic origin of their immiscibility continues to be debated,^18–21^ which is why they provide an interesting yet simple testing ground for our objectives. Specifically, we have previously investigated a mixture of hexane and perfluorohexane in a series of force field molecular dynamics simulations at molar fractions *x* = 0.5. The local (atomic) philicities have gradually been changed by systematically varying the Lennard-Jones energy (ε) and size (σ) parameters within the OPLS^22^ interaction potentials. Subsequently, the effect on the miscibility was analyzed. We found that the miscibility temperature is linearly correlated with all energy parameters and is more sensitive to changes in those of the aliphatic alkanes, whereas the size parameters have less influence on the miscibility temperature overall.^13^ However, some relationships could not be fully explained:

(1) Changes in the size parameters of outer atoms (H, F) have a different, and in some cases even opposite, effect compared to changing the size of the inner carbon atoms (C_H_, C_F_).^13^ Self-diffusion coefficients and pre-exponential factors of diffusion of hexane and perfluorohexane in an equimolar mixture were shown to increase with σ­(H) and σ­(F), while they decrease with σ­(C_H_) and σ­(C_F_). The miscibility temperature slowly increases with σ­(H) and σ­(F) before decreasing again at very large values of σ. In contrast, several maxima were observed in the miscibility temperature as a function of the size of the carbon atoms (σ­(C_H_) and σ­(C_F_)). Transition to the gas phase was observed in the simulations for very large values of σ­(H) and σ­(F), but for very small values of σ­(C_H_) and σ­(C_F_).
(2) For very small values of ε­(F), ε­(C_H_), and ε­(C_F_) below 0.05 kcal/mol, the trend in the miscibility temperature as a function of the energy parameters reverses. Contrary to our intuitive expectations, decreasing ε leads to a higher temperature of mixing.

In this work, we aim at a quantitative explanation for these phenomena, in particular, using a recently published tool for a state-sensitive energy repartition scheme. In this scheme, that we have labeled effective interaction strength,^23^ the species-dependent interaction enthalpy can be decomposed into underlying pairwise atomistic interactions, which in turn can be used to understand why the inter- and intramolecular interaction enthalpies react in a specific manner.

The effective strength of an interaction *U*_ab_^eff^ between atomic species a and b, that are elements of molecules A and B (a ∈ A, b ∈ B), is given by eq 1.

1 $$U_{\mathrm{ab}}^{\mathrm{eff}}=4\pi f_{\mathrm{AB}}\frac{N_{a}N_{b}}{V}\int r^{2}U_{\mathrm{ab}}\left(r\right)g_{\mathrm{ab}}\left(r\right)\mathrm{dr}$$

The pair interaction potential *U*_ab_(*r*) is weighted with the corresponding partial radial density distribution *g*_ab_(*r*). The factor *f*_AB_ is introduced to ensure correct counting of the interaction pairs: for the autointeractions between atoms of the same molecule type (A = B) $f_{\mathrm{AB}}=\frac{1}{2}$ , and for cross-interactions (A ≠ B) *f*_AB_ = 1. *N*_a_ and *N*_b_ are the numbers of atoms a and b, and *V* is the volume of the simulation box. In our analysis, we only consider the Lennard-Jones part of the interaction potential *U*_ab_(*r*) ≈ *U*_ab_^LJ^ (see eq 2)­

2 $$U_{\mathrm{ab}}^{\mathrm{LJ}}=4\varepsilon_{\mathrm{ab}}\left[{\left(\frac{\sigma_{\mathrm{ab}}}{r}\right)}^{12}-{\left(\frac{\sigma_{\mathrm{ab}}}{r}\right)}^{6}\right]$$

since dispersion interactions are dominant in the specific alkane–perfluoroalkane system.^18^ It is straightforward to include other contributions to the interaction potential, such as electrostatic interactions, whenever necessary in different systems.

The total effective interaction energies for the auto- and cross-interactions are obtained by summation of the relevant individual pairwise effective interaction strengths according to eq 3. Autointeractions between hexane molecules are denoted by the subscript “auto, H”, those between perfluorohexane molecules by the subscript “auto, F”, and the cross-interactions between hexane and perfluorohexane molecules by the subscript “cross, HF”.

3 $$\begin{array}{l} U_{\mathrm{auto},H}^{\mathrm{eff}}=U_{H-H}^{\mathrm{eff}}+U_{H-C_{H}}^{\mathrm{eff}}+U_{C_{H}-C_{H}}^{\mathrm{eff}}\\ U_{\mathrm{auto},F}^{\mathrm{eff}}=U_{F-F}^{\mathrm{eff}}+U_{F-C_{F}}^{\mathrm{eff}}+U_{C_{F}-C_{F}}^{\mathrm{eff}}\\ U_{\mathrm{cross},\mathrm{HF}}^{\mathrm{eff}}=U_{H-F}^{\mathrm{eff}}+U_{H-C_{F}}^{\mathrm{eff}}+U_{F-C_{H}}^{\mathrm{eff}}+U_{C_{H}-C_{F}}^{\mathrm{eff}} \end{array}$$

Finally, the difference between the effective interaction energies of the cross-interactions and the autointeraction yields an effective energy of mixing Δ*U*_mix_^eff^ (eq 4). This quantity represents the enthalpic contribution to the state of mixing.

4 $$\Delta U_{\mathrm{mix}}^{\mathrm{eff}}=U_{\mathrm{cross},\mathrm{HF}}^{\mathrm{eff}}-\left(U_{\mathrm{auto},H}^{\mathrm{eff}}+U_{\mathrm{auto},F}^{\mathrm{eff}}\right)$$

Due to the dependency on the state of mixing, the total energy of mixing does not represent the total energy difference between the mixed and phase-separated states in the manner of a reaction enthalpy. Instead, it measures the differential energy for the transition of one particle from the mixed to the separated phase in the specific instantaneous mixing state of the system. Thus, it rather has the character of a chemical potential for the two phases; for a regular chemical reaction, the chemical potential of the educt and product sides of the reaction is also dependent on the reaction progress.

Our primary aim is to understand the enthalpic driving forces responsible for the phase separation and mixing phenomena in molecular liquids. Particular focus is on the response of these interactions to changes in the chemical nature of the atoms. While decomposing the individual interactions at the atomic level, we also aim to incorporate adequate phase-space sampling via molecular dynamics simulations. Using an alkane–perfluoroalkane mixture modeled with the OPLS force field^22^ as an example, we compute the interaction energy contributions that lead to a mixed-phase state versus a phase-separated state separately. By analyzing the effect of atomic interaction parameters on phase behavior, we obtain a semiquantitative measure of the philicity of our mixture’s molecular components.

## Methods

This work builds on a data set of force field molecular dynamics (MD) simulations of a hexane–perfluorohexane system with modified interaction parameters that was curated in a previous study. Specifically, a cubic box containing 250 hexane and 250 perfluorohexane molecules was used. MD runs were performed for 10 ns starting from an initially separated configuration in two layers. In each run, a single Lennard-Jones parameter (ε­(H), ε­(C_H_), ε­(F), ε­(C_F_), σ­(H), σ­(C_H_), σ­(F), σ­(C_F_)) was modified from its original OPLS-AA^22,24^ value. Of course, the OPLS functional form also implements Coulomb interactions, which contribute to the configurational state sampled in the trajectory. However, preliminary results showed that the effects of the partial charges were negligible, in line with the understanding that dispersion forces dominate electrostatic interactions in this specific system.^18^ Thus, this work only considers the impact of changes in the Lennard-Jones term. For a detailed simulation protocol and complete data tables for all performed simulations, please refer to ref 13. The total densities of the simulation box as a function of the size parameters, as obtained during the equilibration runs and reported in ref 13 are plotted in Figure 1. For the sake of comprehensiveness, an analogous plot of the total density as a function of the energy parameters is provided in Figure S2. Isobaric coefficients of thermal expansion were calculated from the change in box volume with temperature for each parameter value. Details are provided in Section 2.2 of the Supporting Information.

**Figure 1:**
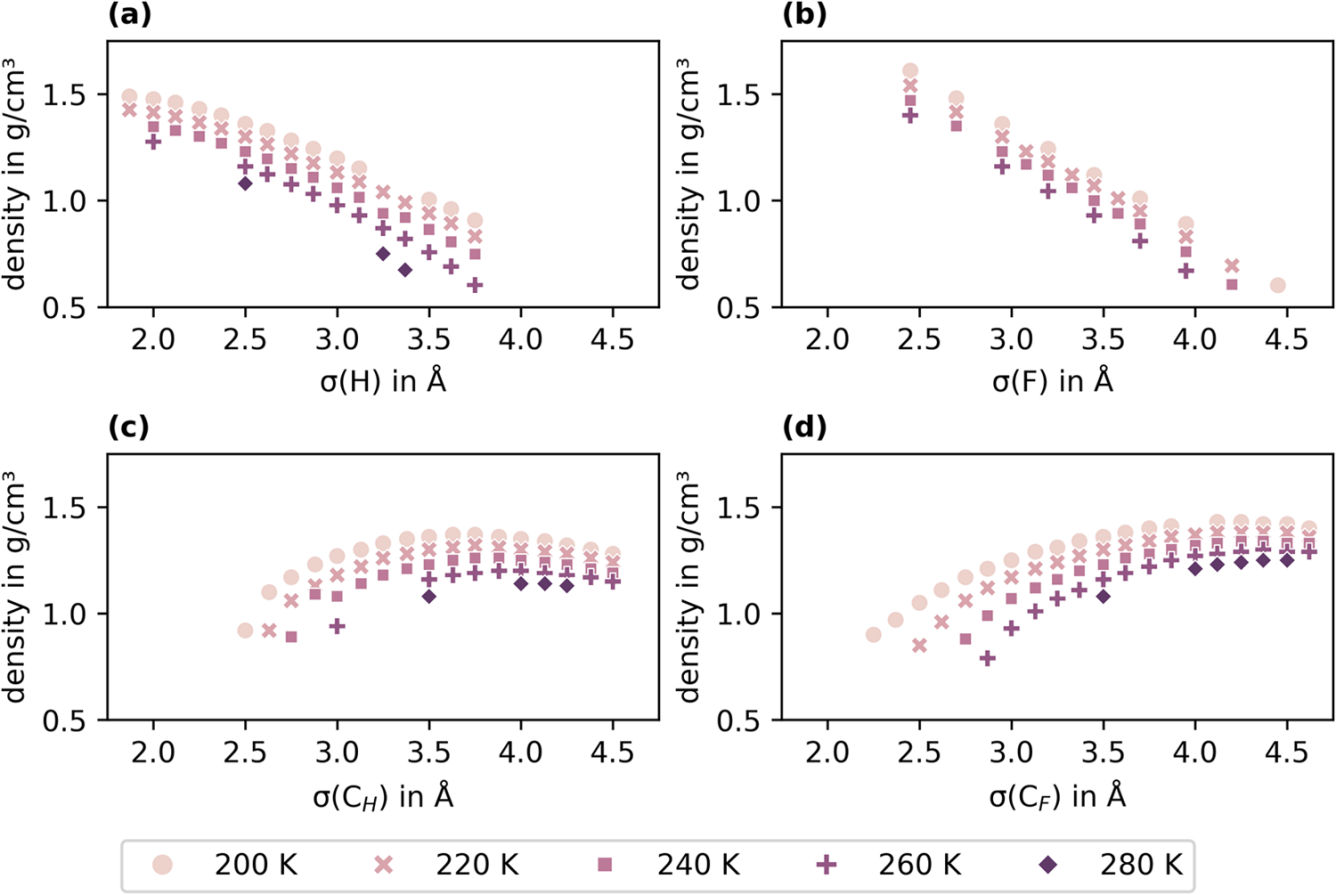
Global density of an equimolar hexane–perfluorohexane mixture as a function of the van der Waals size parameters: (a) σ­(H), (b) σ­(F), (c) σ­(C_H_), and (d) σ­(C_F_).

For the analysis of the effective interaction strengths, a subset of the data set in ref 13 was used. A simulation series from a range of values at a fixed temperature was selected as an example for each Lennard-Jones energy and size parameter. These are

(1) σ­(H): 1.87–3.75 Å (*T* = 220 K)
(2) σ­(C_H_): 2.63–4.5 Å (*T* = 220 K)
(3) σ­(F): 2.45–4.2 Å (*T* = 220 K)
(4) σ­(C_F_): 2.75–4.62 Å (*T* = 240 K)
(5) ε­(H): 0.015–0.06 kcal/mol (*T* = 260 K)
(6) ε­(C_H_): 0.012–0.093 kcal/mol (*T* = 200 K)
(7) ε­(F): 0.027–0.1 kcal/mol (*T* = 240 K)
(8) ε­(C_F_): 0.026–0.174 kcal/mol (*T* = 240 K)

For each trajectory within these eight series, the ten different atom–atom radial distribution functions *g*_ab_(*r*) were computed with data points taken every 100 fs using TRAVIS.^25,26^ Then, *r* ^2^ *U*_ab_(*r*)*g*_ab_(*r*) was numerically integrated with the scipy.integrate.trapezoid method^27^ and subsequently multiplied with the corresponding prefactor to yield the effective interaction strength *U*_ab_^eff^ according to eq 1. The integral values and prefactors used are provided in Tables S1–S8. Plots were generated using the Python libraries Matplotlib^28^ and Seaborn.^29^

## Results and Discussion

As a starting point, we provide an overview of the structural response of the hexane–perfluorohexane system at a molar fraction *x* = 0.5 to variations in the Lennard-Jones size parameters. This helps to rationalize the previously observed changes in the miscibility temperature and diffusion in terms of more fundamental effects at the structural level. The latter differ for changing the size of inner and outer atoms, which is illustrated and quantified by the effective interaction strengths of individual atom–atom interactions. In the second part, we use the effective interaction strength to analyze changes in the Lennard-Jones energy parameters and to explain their effect on the configurational state of the system. Finally, we compare the relative importance of the force field parameters with regard to the philicity mismatch between alkanes and perfluoroalkanes. The actually observed state of mixing results from the joint contributions of all of the individual pairwise interactions.

### Impact of Atomic Size Parameters

In a previous study, we identified differences in the response of dynamic properties of hexane and perfluorohexane molecules to changes in the size parameter of outer hydrogen and fluorine atoms versus changes in the size parameter of the inner carbon atoms.^13^ Here, we observe similar differences in the dependency of static properties such as density and coefficients of thermal expansion on the magnitude of the atomic size parameters σ. Figure 1 shows the density of an equimolar hexane–perfluorohexane mixture as a function of σ­(H), σ­(F), σ­(C_H_), and σ­(C_F_). The data was retrieved from the simulation data set in ref 13. Note that, instead of the negative excess density leading to large positive excess volumes for alkane–perfluoroalkane mixtures,^14,30^ we are examining the global density of the system in our simulation at varying degrees of mixing dependent on temperature and Lennard-Jones size parameter choice. There are no changes in the temperature dependency of the density. It decreases with temperature in all simulation series, which is fully consistent with the expected behavior. Changes in density as a function of the atomic size are, however, more complex. Increasing σ­(H) and σ­(F) reduces the density down to very small values. As with dynamic properties, the effect of changes to the carbon atom size is different. Only at σ­(C_H_) > 3.75Å, and σ­(C_F_) > 4.25 Å, there is a small decrease in the global density with σ. At lower values of σ­(C_H_) and σ­(C_F_), the density is higher the larger the parameter. Opposite trends can be observed in the isobaric coefficients of thermal expansion, which were calculated from the change in box volume with temperature for the different parameter values. The isobaric coefficient of thermal expansion decreases with σ­(C_H_) and σ­(C_F_), while it increases with σ­(H) and σ­(F) (cf. Figure S3).

Qualitatively, the observed trends in density and coefficients of thermal expansion can be understood with a simple model concept. Increasing the size of hydrogen and fluorine atoms (σ­(H) and σ­(F)) leads to expansion of the whole system, lower densities, and consequently weaker interactions. Since intermolecular interactions are inversely correlated to thermal expansion coefficients,^31,32^ the coefficients of thermal expansion are also higher the larger σ­(H) and σ­(F). In contrast to this, it is not as simple to explain the exact reason why increasing the size of carbon atoms (σ­(C_H_), σ­(C_F_)) does not induce the same but rather opposite changes in density and thermal expansion coefficients. It stands to reason that this is related to the different effective atomic distances. The carbon atoms within the molecules are further away from the other atoms, which affects intermolecular interactions differently.

In the following section, we will examine this reasoning, illustrate the design idea behind the effective interaction strength, and explain the connection to the concept of molecular philicity.

For this purpose, we examine the effect of increasing the size parameters σ­(F) and σ­(C_F_) by 10%. The elementary functions of the calculation of the effective interaction strength are compared in Figure 2. Specifically, we analyze the differences in the interatomic potentials *U* ^LJ^(*r*), the pair correlation functions, and the effective interaction potential *r* ^2^ *U* ^LJ^(*r*) *g*(*r*) of the F–F and C_F_–C_F_ interactions upon changing σ­(F) from 2.95 Å to 3.2 Å, and σ­(C_F_) from 3.5 Å to 3.87 Å, respectively.

**Figure 2:**
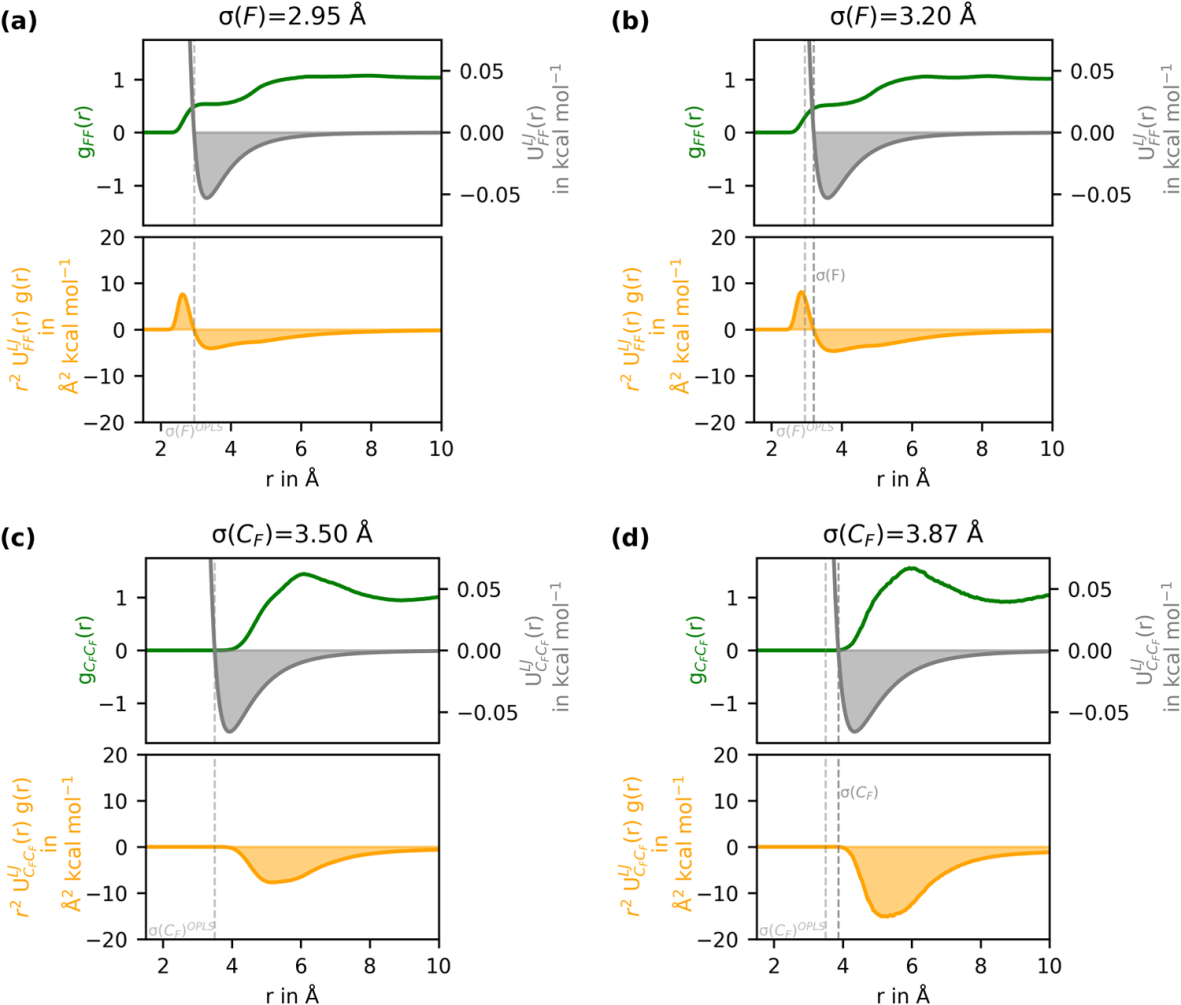
Elementary steps of the calculation of the effective interaction strength of the F–F (top) and C_F_–C_F_ (bottom) interactions in an equimolar hexane–perfluorohexane mixture. (a, c) Simulation with unmodified OPLS-AA^22,24^ parameters. Right: (b) simulation with modified size of fluorine (σ­(*F*) = 3.2Å) and (d) simulation with modified size of perfluorohexane carbon atoms (σ­(C_F_) = 3.87Å). In each subfigure, the upper panels show the pairwise interaction potential *U*_AB_^LJ^ (gray) and the radial distribution function *g*_AB_(*r*) (green). The lower panels show the effective interaction potential *r* ^2^ *U*_AB_^*LJ*^(*r*)*g*_AB_(*r*) (orange) as the integrand of eq 1.

Increasing σ shifts the potential energy minimum to larger distances while the potential well is broadened at the same time (compare the gray lines from left to right). In the case of the F–F interaction, the region of the potential well overlaps to a large extent with the distance distribution of intermolecular F–F pairs, resulting in an overall net attraction (see Figure 2a). Some fluorine atoms are too close to each other, such that the effective potential is repulsive at short distances, but this is outweighed by the larger attractive region. As σ­(F) is increased, the F–F radial distribution function is shifted along with the change in the Lennard-Jones potential, so that the shape of the integrand of eq 1 does not change. The broadened potential well, however, leads to a small strengthening of the number-weighted effective interaction strength from −419 kcal/mol to −468 kcal/mol. It is interesting to note that the density decreases upon increasing σ­(F) even though the attractive interactions between fluorine atoms are effectively becoming stronger. This is due to the fact that the other nine atom–atom interactions are partially negatively correlated with the increase in σ­(F) (cf. Table S3), leading to less attraction between the molecules and expansion of the system.

In contrast to this, the overlap between the potential well and the distance distribution of the C_F_–C_F_ interaction is much smaller for the OPLS value of σ­(C_F_) (see Figure 2c). There are no atom pairs in the most attractive region around the minimum of the interaction potential. This changes as σ­(C_F_) is increased. While the potential well is broadened and its minimum shifted to a larger distance, the pair correlation function between perfluorohexane carbon atoms shows little change, resulting in a larger overlap and a much more attractive effective interaction (see Figure 2d). A 10% increase in σ­(*C*_*F*_) almost doubles the number-weighted effective interaction strength from −173 kcal/mol to −337 kcal/mol. Together with the analogous strengthening of the other interactions with *C*_*F*_ atoms (compare Table S4), this explains the compression of the system and the increase of the density with σ­(C_F_). At very large values of σ­(C_F_) ≥ 4.12, we observe analogous behavior to increasing σ­(F) with a small but growing repulsive part of the effective potential, as depicted in Figure S1. This phenomenon is reflected in the slight decrease in density (Figure 1), where the larger molecule size with increasing σ does not allow for equally close packing.

We have illustrated the different responses of the radial distribution functions and effective interaction strengths upon varying σ­(F) and σ­(C_F_) by also plotting the difference functions according to eq 5 for the F–F interaction and the analogous functions for the C_F_–C_F_ interaction.

5 $$\begin{array}{l} \Delta g_{\mathrm{FF}}\left(r\right)=g_{\mathrm{FF}}{\left(r\right)}^{\left[\sigma \left(F\right)=3.20\right]}-g_{\mathrm{FF}}{\left(r\right)}^{\left[\sigma \left(F\right)=2.95\right]}\\ \Delta \left(r^{2}U_{\mathrm{FF}}^{\mathrm{LJ}}\left(r\right)g\left(r\right)\right)={\left(r^{2}U_{\mathrm{FF}}^{\mathrm{LJ}}\left(r\right)g_{\mathrm{FF}}\left(r\right)\right)}^{\left[\sigma \left(F\right)=3.20\right]}-{\left(r^{2}U_{\mathrm{FF}}^{\mathrm{LJ}}\left(r\right)g_{\mathrm{FF}}\left(r\right)\right)}^{\left[\sigma \left(F\right)=2.95\right]} \end{array}$$

Figure 3b clearly shows how the increased occurrence of C_F_–C_F_ distances between 4 and 7 Å strongly enhances the effective interaction strength. The comparatively smaller strengthening of the F–F interaction (Figure 3a) results from the counteracting repulsive contributions at short distances, which decrease as σ­(F) is increased.

**Figure 3:**
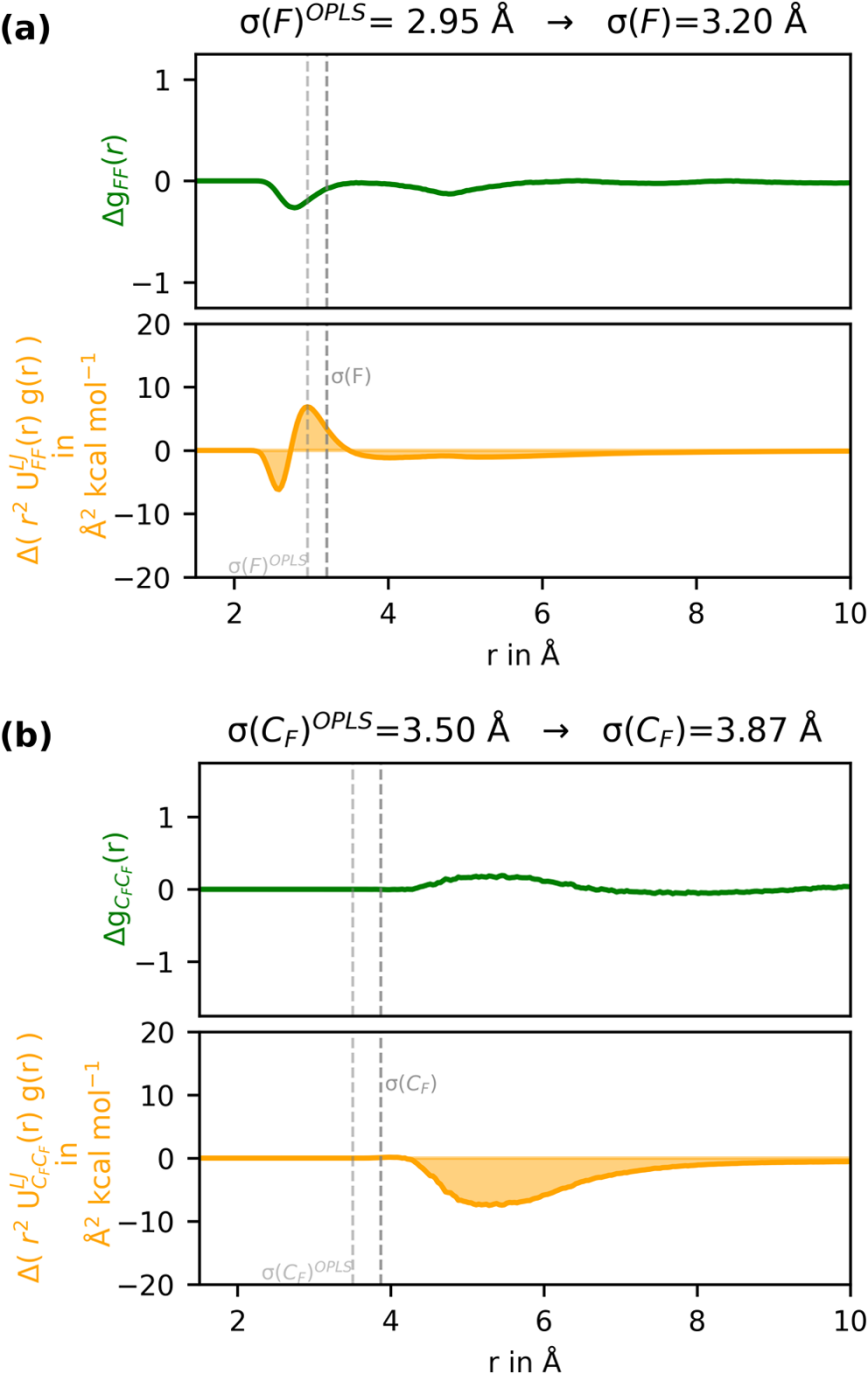
Illustration of the response of the radial distribution function and the effective interaction potential to a particular variation in the size parameter σ. Difference functions were calculated according to eq 5. (a) Response to increasing σ­(F) from 2.95 Å to 3.20 Å. (b) Response to increasing σ­(C_F_) from 3.5 Å to 3.87 Å.

The detailed analysis given in Figures 2 and 3 illustrates the underlying mechanism that leads to the specific numerical value of the effective interaction energy *U*_ab_^eff^ for a given atom pair a, b, and given interaction energy parameters σ, ε, as the result of integrating the distribution weighted pair potentials (eq 1). We now turn to the analysis of the dependency of these effective interaction strength features upon variation of the interaction parameters.

The main goal of this work is to decipher the interplay of intermolecular interactions between the atomic species among all pairs of atom types (hydrogen–hydrogen H–H, hydrogen–fluorine H–F, hydrogen–carbon H–C_F_, ...) and to identify how they control the effective miscibility of the binary system as a whole. The interactions are grouped based on their tendency to favor a mixed-phase state or a phase-separated state. Subsequently, the sensitivity of the individual effective interaction strengths upon a (relative) variation of the Lennard-Jones size and energy parameters is investigated. This measures the effective action of the fundamental force field coefficient, determining if increasing the specific value leads to mixed or separated phases. Equation 4, in turn, yields a direct measure of the philicity match/mismatch between the two components of our mixture. Thus, the analysis tool of the effective interaction strength provides a semiquantitative interpretation of the philicity concept.

Figure 4 shows all effective interaction strengths calculated from eq 1 as a function of the size parameters. First of all, it becomes clear that all ten atom–atom interactions are influenced by changes in σ, regardless of whether they involve the respective atom type or not. The extent of the influence varies, however.

**Figure 4:**
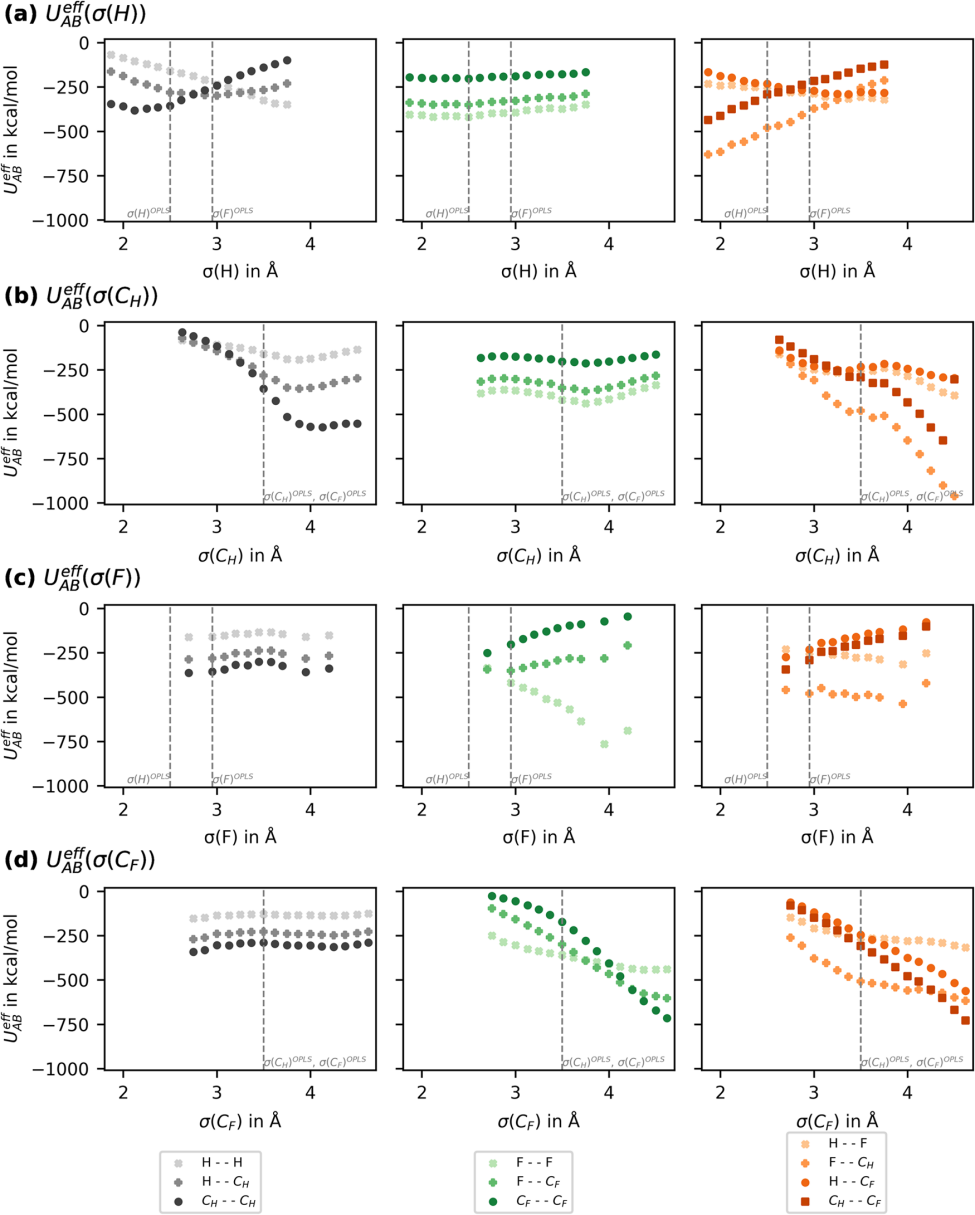
Effective interaction strengths of the atomic pairwise van der Waals interactions in a hexane–perfluorohexane mixture as a function of the size parameters σ: (a) *U*_AB_^eff^(σ­(H)) at 220 K, (b) *U*_AB_^eff^(σ­(C_H_)) at 220 K, (c) *U*_AB_^eff^(σ­(F)) at 220 K, and (d) *U*_AB_^eff^(σ­(C_F_)) at 240 K. The left column groups hexane autointeractions, the middle column groups perfluorohexane autointeractions, and the right column groups cross-interactions. The vertical dashed lines indicate the values of the size parameters in the OPLS-AA force field.^22,24^

It can be distinguished between direct and indirect impact on the effective interaction strength. Direct impact means the response of *U*_AB_^eff^ to changes of the parameters of either atom type A or B, while indirect impact means the response of *U*_AB_^eff^ to a variation of any other atomic parameter (≠ A, B). For the latter, the change of *U*_AB_^eff^ originates purely from variations in the liquid structure, i.e., the distance distribution functions *g*_AB_(*r*).

The direct impact is generally much stronger than the indirect one, such that autointeractions between atoms of the molecule type for which σ is changed are strongly dependent on σ. The autointeractions between atoms of the other molecule type are only mildly affected. The cross-interactions show different dependencies, depending on whether the respective atom type is involved or not. This becomes especially clear for changes in the carbon atom size (compare Figure 4b,d). In contrast, the impact of changes in the size of the outer atoms (H and F) is more complex. For instance, increasing σ­(H) from the OPLS value of 2.5–2.95 Å, the OPLS value of fluorine, strongly impacts the H–H, H–C_H_, and C_H_–C_H_ interactions (compare Figure 4a). Interestingly, the H–H interaction is positively correlated to σ­(H), meaning that a larger value of σ results in a more attractive effective interaction strength (negative values), whereas the H–C_H_ and C_H_–C_H_ interactions exhibit a negative correlation (positive values). The C_H_–C_F_ and F–C_H_ interactions exhibit such a negative correlation to σ­(H), too. Even though these interactions are only indirectly impacted by changes in the size of hydrogen atoms, the absolute magnitude of the impact is surprisingly strong. Changes in σ­(F) result in equivalent impacts on the effective interaction strengths (compare Figure 4c).

### Impact of ε on Interaction Strength

Figure 5 shows all interatomic effective interaction strengths as functions of the Lennard-Jones energy parameters ε­(H), ε­(C_H_), ε­(F), and ε­(C_F_). Similarly to the previously discussed size parameters, the impact of changes in the energy parameters on the effective interaction strength is of higher magnitude if the respective pairwise interaction is directly affected by the parameter; indirect effects are much weaker. This is particularly evident for changes in ε­(F) (see Figure 5c), but also applies to the other energy parameters. As expected, upon increasing the depth of the interaction potential well, all effective interaction strengths involving the respective atoms are strongly positively correlated to the strength of attraction, i.e., to the value of ε. It also meets expectations that this effect is greater for autointeractions than for cross-interactions. The indirect influence on all interactions that do not involve the respective atom type is comparatively much weaker. Autointeractions between the other molecular types are almost independent of ε. Cross-interactions show an intriguing dependence on the energy parameters. At large values of ε, there are small positive correlations. This is because, at these values of the energy parameters, autointeractions are so strongly preferred that cross-interactions become less common. It is also noteworthy that the effective interaction strength of the F–C_H_ interaction is more strongly influenced by changes in all of the energy parameters than the other individual cross-interactions. This indicates that this pairwise interaction is particularly relevant in terms of the philicity of the two components. Thus, it could act as a descriptor for the miscibility of alkanes and perfluoroalkanes, which we will examine in more detail in a later section discussing the relative impact of changing the energy and size parameters.

**Figure 5:**
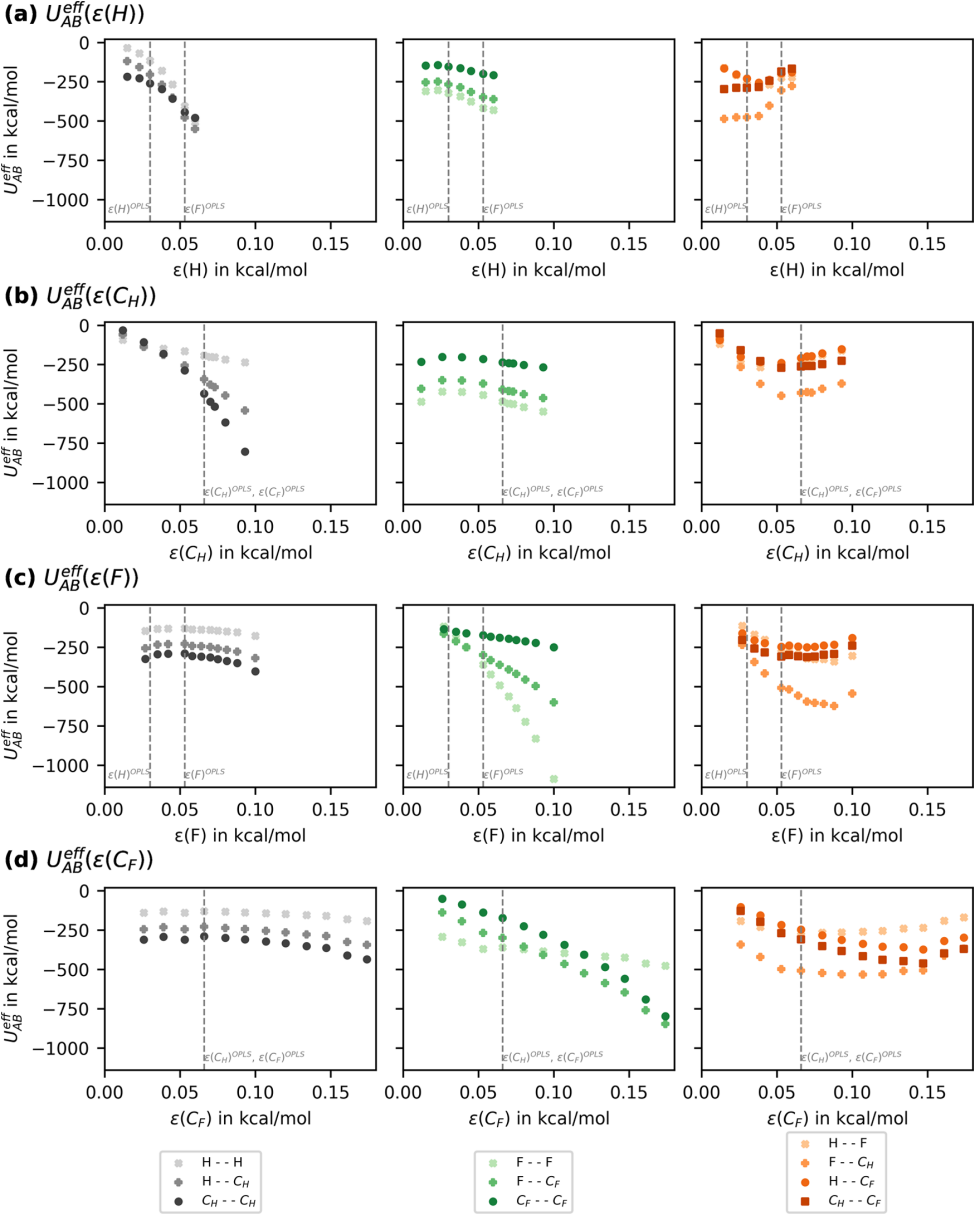
Effective interaction strengths of the atomic pairwise van der Waals interactions in a hexane–perfluorohexane mixture as a function of the energy parameters ε: (a) *U*_AB_^eff^(ε­(H)) at 260 K, (b) *U*_AB_^eff^(ε­(C_H_)) at 200 K, (c) *U*_AB_^eff^(ε­(F)) at 240 K, and (d) *U*_AB_^eff^(ε­(C_F_)) at 240 K. The left column groups hexane autointeractions, the middle column groups perfluorohexane autointeractions, and the right column groups cross-interactions. The vertical dashed lines indicate the values of the energy parameters in the OPLS-AA force field.^22,24^

The analysis of the individual effective interaction strengths also offers a tool to explain the previously reported seemingly paradoxical behavior of a trend toward phase separation, the smaller the energy parameter for very small values of ε­(F), ε­(C_F_), and ε­(C_H_) smaller than 0.05 kcal/mol.^13^ The intuitive perception is that a reduction of ε­(F) (ε­(F)^OPLS^ = 0.053 kcal/mol) leads to an assimilation of the philicities of perfluoroalkane and alkane (with a corresponding ε­(H)^OPLS^ = 0.03 kcal/mol). Thus, autointeractions between perfluoroalkane molecules should become less attractive, whereas cross-interactions should be enhanced, leading to mixing rather than phase separation. However, only parts of this hypothesis are confirmed by analysis of the effective interaction strengths, as shown in Figure 6. Indeed, all effective interaction strengths of perfluorohexane autointeractions are reduced (i.e., shifted to less negative values) upon shrinking ε­(F) from 0.042 to 0.027 kcal/mol. The cross-interactions involving fluorine atoms are directly influenced by the same effect. Interestingly, the reduction in effective interaction between unlike molecules also occurs indirectly in those cross-interactions that do not involve fluorine atoms (H–C_F_ and C_H_–C_F_). The surprising point is now that the magnitude of the reduction in effective cross-interaction strengths exceeds that of the autointeractions, so that the system as a whole has a lower degree of mixture at ε­(F) = 0.027 kcal/mol. This indirectly even strengthens the autointeractions between hexane molecules.

**Figure 6:**
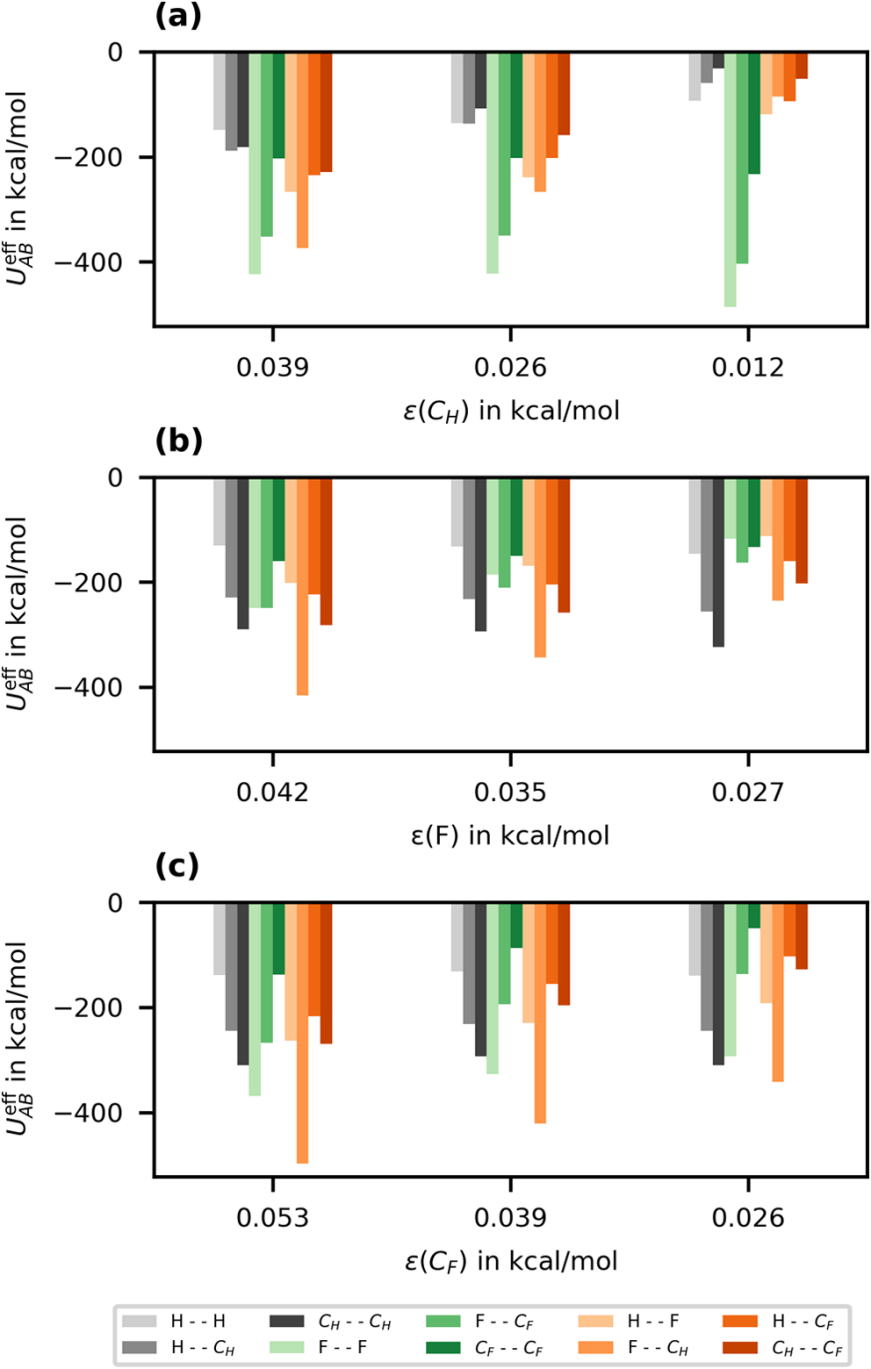
Comparison of the effective interaction strengths at progressively smaller energy parameters (a) ε­(C_H_) at 200 K, (b) ε­(F) at 240 K, and (c) ε­(C_F_) at 240 K. Gray bars are used for hexane autointeractions, green bars for perfluorohexane autointeractions, and orange bars for cross-interactions.

Similar patterns are observed for decreasing ε­(C_F_) from 0.053 to 0.026 kcal/mol and ε­(C_H_) from 0.039 to 0.012 kcal/mol. In both cases, the strength of autointeractions between the corresponding molecule type is reduced, but the reduction in the cross-interaction strength is even larger, thereby enhancing demixing. Only the effect on the autointeractions of the other molecule type is not quite as pronounced.

### Joint Effect of Size and Energy Parameters: A Comprehensive View of Miscibility

To compare the impact of the individual atomic parameters on the philicity difference between alkanes and perfluoroalkanes and thus on the mixing behavior, we have computed the relative sensitivity of the effective interaction strengths from the curves in Figures 4 and 5. This was achieved by calculating the slopes $\frac{{\mathrm{dU}}_{\mathrm{AB}}^{\mathrm{eff}}\left(\sigma \right)}{d\sigma }$ and $\frac{{\mathrm{dU}}_{\mathrm{AB}}^{\mathrm{eff}}\left(\varepsilon \right)}{d\varepsilon }$ within finite limits at the points where σ or *ε* equal their respective OPLS value. Subsequently, the slopes were multiplied with the respective parameter value for normalization. Numerical values are provided in Tables S9–S11. Figure 7 visualizes the resulting properties $\sigma \frac{{\mathrm{dU}}_{\mathrm{AB}}^{\mathrm{eff}}\left(\sigma \right)}{d\sigma }$ and $\varepsilon \frac{{\mathrm{dU}}_{\mathrm{AB}}^{\mathrm{eff}}\left(\varepsilon \right)}{d\varepsilon }$ as separate heatmaps for the auto- and cross-interactions. An enhancement of the individual effective interaction strength (corresponding to a more negative *U*_AB_^eff^ upon an increase of the σ or *ε* parameter) is color-coded with a blue color gradient, while a weakening is colored with a red gradient. For the autointeractions, an enhancement of the interactions leads toward phase separation, whereas weaker interactions lead to a mixing tendency. The opposite applies to the cross-interactions, where stronger interactions favor mixing and weaker interactions favor phase separation. Hence, an atomic property parameter can be considered a sensitive descriptor for the philicity (difference) if its variation yields a strong intensification (blue color code in Figure 7) of autointeractions and/or a strong weakening of cross-interactions (red color code).

**Figure 7:**
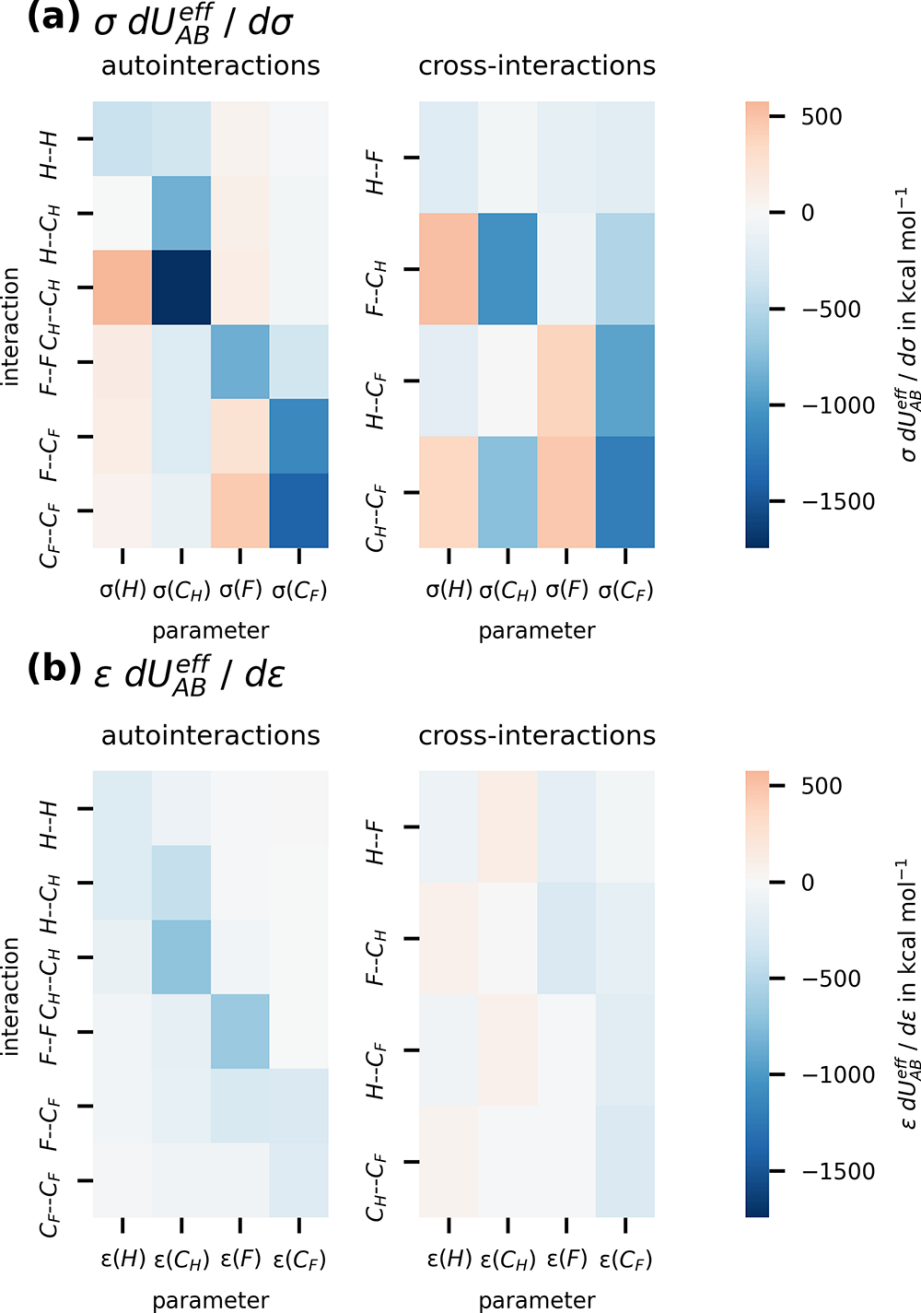
Relative change in the effective interaction strength when the parameters are changed around their OPLS values. Normalized with the respective OPLS values. (a) Impact of Lennard-Jones size parameters, (b) impact of Lennard-Jones energy parameters. Positive correlations, i.e., strengthening of the interaction in question, are represented by a blue gradient. Negative correlations, i.e., weakening of the respective interaction, are represented by a red gradient. For the autointeractions, blue colors correspond to an increased tendency for phase separation and red colors to an increased tendency for mixing. The opposite applies to the cross-interactions.

Increasing the carbon size parameters σ­(C_H_) and σ­(C_F_) compared to their OPLS value exerts the strongest relative impact on the effective interaction strengths (see Figure 7a). However, both auto- and cross-interactions are enhanced so that they partially cancel each other out. The slightly stronger impact on the effective strengths of the autointeraction indicates an intensification of the philicity difference and the tendency toward separation. An opposite feature is observed for the size of the outer atoms (σ­(H) and σ­(F)): increasing their size enhances the strength of direct interactions with the respective atom type solely. The indirect impact via structural changes on the other interactions (i.e., which do not involve the given atom type) leads to decreases in the effective interaction strengths of both auto- and cross-interactions. Compared to the carbon size parameters, there is a greater extent of compensation between the individual interactions. On the one hand, the effects cancel each other out within the auto- and cross-interactions. On the other hand, auto- and cross-interactions have opposite effects on the difference in philicity. No clear trend in mixing behavior can be derived from this, which is in line with the findings of our earlier study, where the state of mixing was determined from an analysis of the cross-radial distribution functions.^13^ The striking differences between outer (H, F) and inner (carbon) atoms can be explained in analogy to the analysis of the density. The interactions originating from these two atom categories are governed by different distance ranges of the interaction potentials. With the outer atoms, the most frequent interatomic distances coincide with the potential well region, leading to an overall attraction. At the same time, repulsive contributions are important because of the short interatomic distances. Now, an increase in the size parameter of the outer atoms increases the weight of the repulsive contributions. This contributes to the complex interplay of counteracting effects, resulting in no clear tendency in the miscibility. In contrast, it is the long-range region that defines interactions with carbon atoms. Here, the shifts of the potential well and the change in the amplitude of the attractive parts of the Lennard-Jones potential upon increasing the size parameter lead to stronger effective interactions.

The relative impact of increasing the Lennard-Jones energy parameters is smaller compared to the impact of changing the size parameters (see Figure 7b). This is at first sight surprising, as one intuitively expects changes in the depth of the interaction potential to be more significant than changes in the size parameter. However, the value of σ determines not only the effective spatial range of the interaction potential but also the functional shape of its attractive part. Increasing the size parameter enlarges the volume of the potential well enclosed by the *R* ^–6^ term. This translates to a larger relative impact of changes in σ compared to changes in *ε* upon spatially integrating the occurrence-weighted interaction potential to yield the effective interaction strength. In addition, the distribution of significance for the individual atom types differs from that observed for the size parameters. Among the energy parameters, ε­(C_H_) and ε­(F) cause the strongest, yet relatively mild response in the effective strengths of the autointeractions. The strength of the cross-interactions is very weakly influenced by changes in the energy parameters, so that their contribution to philicity is negligible. This results in an overall tendency toward phase separation when energy parameters are increased. The greater significance of hexane carbon atoms and fluorine atoms is consistent with the findings of Pollice et al. that dispersion interactions primarily originate from carbon atoms in alkanes and from fluorine atoms in perfluoroalkanes.^18^ As a result, the interaction between these two atom types accounts for a large portion of the total energy of mixing, and changes in the energy parameters have a particularly strong effect on this interaction.

Interestingly, changes in the size and energy parameters of perfluoroalkane carbon atoms have a smaller impact on all intermolecular interactions than alkane carbon parameters, despite the two types of carbon atoms having the same numerical value in the OPLS-AA force field.^22,24^ This observation points to a noteworthy aspect related to the perceived versus factual relevance of the energy and distance parameters for Lennard-Jones-type interactions. An intuitive assessment yields that (a) the energy parameter *ε* determines the strength of the interaction and the distance parameter σ stands for the switchover from attractive to repulsive forces, and (b) the parameters of the atoms in direct contact (here: H and F) are most relevant. However, our analysis of the sensitivity of the effective interaction strength values on parameter variations shows that the most decisive parameter for the determination of the molecular philicity is actually the distance parameter of the carbon atoms, followed by the distance parameters of the outer atoms. Hence, both “intuitive assessments” are misleading, which is a point that merits a more explicit consideration in both the design of force fields and the interpretation of philicity.

So far, we have analyzed and compared the importance of individual pairwise atomic interactions with respect to explaining philicity differences. In order to go a step beyond the direct numerical analysis in terms of the effective interaction strengths, we have also checked the predictive capability of their consolidated values (i.e., the energy of mixing Δ*U*_mix_^eff^ according to eq 4) for the visually observable mixing/separation phase transition of the alkane–perfluoroalkane mixture. Equation 4 condenses the concept of molecular philicity match/mismatch into a single number by balancing the total effective interaction energies of the auto- and cross-interactions (*U*_auto, H_^eff^, *U*_auto, F_^eff^, and *U*_cross, HF_^eff^), that each are the sum of the contributing effective interaction strengths according to eq 3.

In Figure 8, the total energy of mixing Δ*U*_mix_^eff^ is plotted along with its partial constituents as a function of some of the Lennard-Jones parameters. Plots corresponding to the other parameters are provided in Figure S5. Vertical dashed lines mark the critical values of those parameters at which the phase transition (mixed/phase separated) is actually observed in the molecular dynamics simulations. This observation was made by combining the evaluation of radial distribution functions and configurational entropy of mixing.^13,33^ In simplified terms, the parameter ranges labeled with “separated” correspond to configurations where more molecules of one kind are surrounded by more molecules of the same type than of the other type, as indicated by the peak height of the cross-radial distribution function. The horizontal line in Figure 8 represents the entropic contribution to the mixing free enthalpy, i.e., *T*Δ*S*_mix_, in the respective molecular configuration, as obtained from the computational approach presented in refs 33 and 13. These calculations yielded a value of Δ*S*_mix_ = 0.11*R* for the molar mixing entropy.

**Figure 8:**
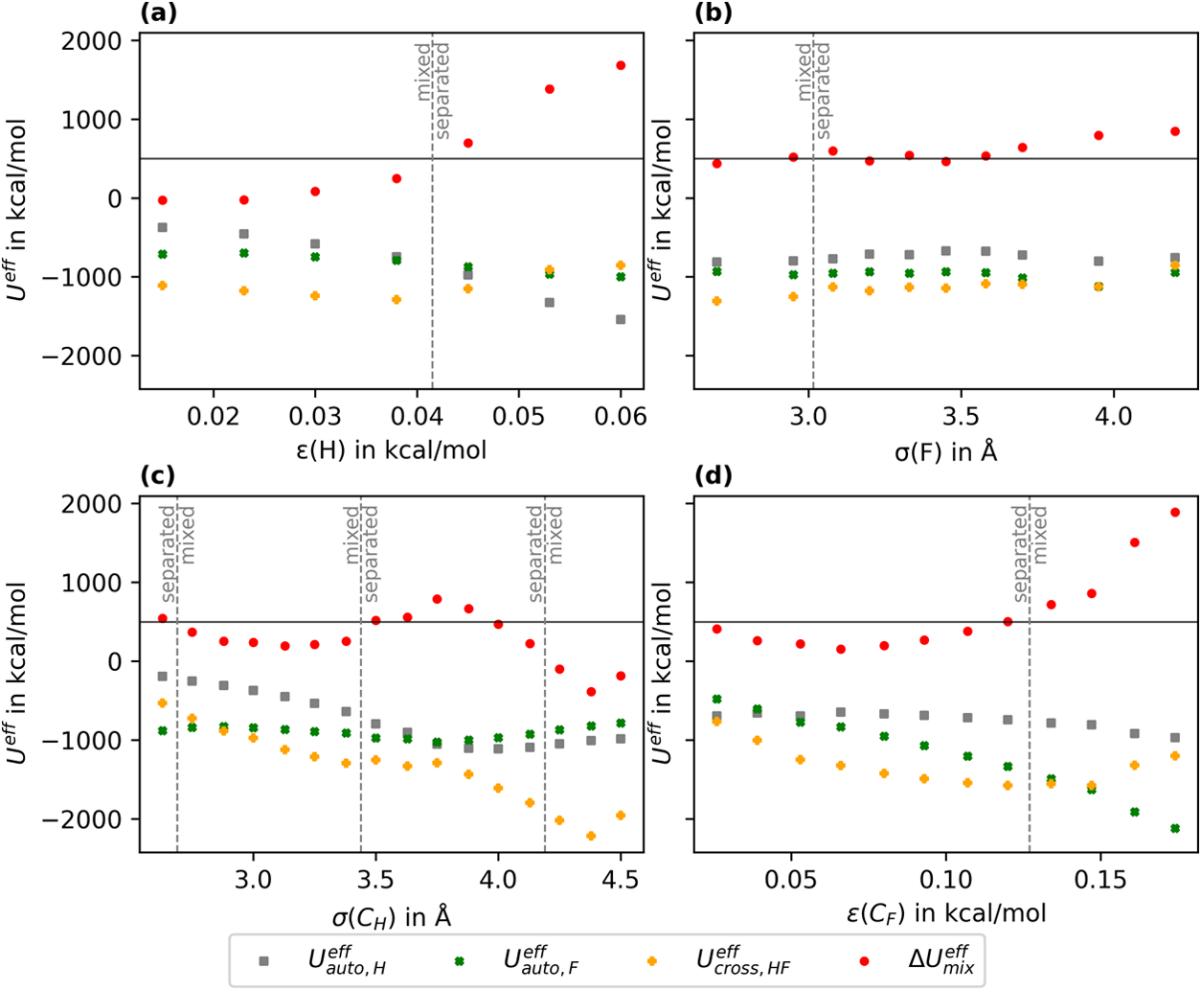
Effective interaction energies *U*_auto, H_^eff^, *U*_auto, F_^eff^, and *U*_cross, HF_^eff^ obtained from eq 3 and effective energy of mixing Δ*U*_mix_^eff^ obtained from eq 4 as a function of some Lennard-Jones parameters. (a) variation of ε­(H) at 260 K, (b) variation of σ­(F) at 220 K, (c) variation of σ­(C_H_) at 220 K, and (d) variation of ε­(C_F_) at 240 K. The vertical dashed lines indicate the mixed/separated phase transitions reported in the simulation data set in ref 13. The horizontal line at 500 kcal/mol acts as a visual aid to correlate Δ*U*_mix_^eff^ to these observed transitions.

It is striking that the observed phase transitions between mixed and phase-separated configurations (dashed vertical lines) almost perfectly match the intersection points of the mixing enthalpy (red dots) and the temperature-weighted mixing entropy (horizontal line). In other words, this plot confirms the consistency of the fundamental thermodynamic concept that the sign of Δ*G*_mix_ = Δ*H*_mix_ – *T*Δ*S*_mix_ determines the phase state (mixed/phase separated) of the system: all situations for which Δ*G*_mix_ < 0 lead to a mixed state (and vice versa). It should be mentioned that there are certain limitations of the predictive level of this approach. First, the finite system size used here presents a practical issue. Matching the phase-separating behavior of the corresponding macroscopic system^14–17^ would require considerably larger periodic simulation cells. Although this problem is only practical and not conceptual, our previous size analysis indicated that converging the entropy of mixing according to refs 13,33 would require a simulation cell that is at least ten times larger.^34^

A more detailed view of the individual contributions to the total effective energy of mixing Δ*U*_mix_^eff^ reveals further insights into the mixed/separated phase transition. In line with the results from the analysis of the individual effective interaction strengths, the ratio of the effective auto- and cross-interaction energies changes as the mixing/demixing transitions are approached with changes in the Lennard-Jones parameters. For instance, when ε­(H) is increased to values larger than 0.04 kcal/mol, the effective hexane autointeractions *U*_auto, H_^eff^ become stronger than the effective perfluorohexane autointeractions *U*_auto, F_^eff^ (Figure 8a, gray and green points). The effective cross-interactions (yellow points) become less prominent, on the other hand.

## Conclusion

In summary, our simulations of the hexane–perfluorohexane mixtures reveal a detailed numerical perspective of the mixing/demixing phase transition and its fundamental thermodynamic driving forces in terms of the two competing philicities. One of the common questions in this field is “Why do A and B not mix (at ambient conditions)?” This is, in principle, a simple consequence of the sign of the mixing free enthalpy. At the outset of this work, we anticipated that, at least for the exemplary system investigated here, we would be able to offer a relatively straightforward conclusion along the lines of “it is mainly because of the van der Waals interactions characterized by the Lennard-Jones parameter ε­(F)”. However, the answer to the question of the nature of the different philicities is more complex and involves contributions from different interatomic interactions that partially compensate each other. Using our technique to decompose the total energy of mixing, Δ*U*_mix_^eff^, into effective interaction strengths, we can elucidate the complex interplay of the direct and indirect enthalpic effects of a set of atomic parameters that lead to the phase behavior that is commonly known as lipophilicity and fluorophilicity.

Changes in the philicity of the molecules, as represented by their atomic Lennard-Jones parameters, induce changes in miscibility by directly and indirectly impacting the interaction strengths. The state of mixing in a given simulation can be assessed using the configurational entropy approach.^33^ On the other hand, an analysis based on effective interaction strengths provides an enthalpic explanation of this mixing state, decomposed into the contributions and relevance of individual interactions.

## Supplementary Material



## Data Availability

A data set of all atom–atom radial distribution functions used is freely available under the following DOI: 10.5281/zenodo.18889911.

## Notes

Complete tables for calculating the effective interaction strength and derived quantities; additional results for density, thermal expansion coefficients, and mixing energies (PDF)
