# Supplementary material for: From Atomic Interactions to Molecular Miscibility and Philicity: Deciphering Enthalpic Driving Forces
Source: J Phys Chem A. 2026 Jun 22;130(26):5045–56. doi: 10.1021/acs.jpca.6c01567 (PMC13339641; doi:10.1021/acs.jpca.6c01567)
Supplement: Supplementary file 1 [file jp6c01567_si_001.pdf]

Supporting Information
–
From Atomic Interactions to Molecular Miscibility and Philicity: Deciphering Enthalpic Driving Forces

Anna Luisa Upterworth[1] and Daniel Sebastiani*[1]

[1]Department of Chemistry, Martin Luther University Halle-Wittenberg, 06120 Halle, Germany

*Email: daniel.sebastiani@chemie.uni-halle.de

Contents

1 Calculation of effective interaction strengths and derived quantities S3

1.1 Effective interaction strengths as a function of the Lennard-Jones size parameters S3
1.2 Effective interaction strengths as a function of the Lennard-Jones energy parameters S21
1.3 Elementary functions of large perfluorohexane carbon size S35
1.4 Relative sensitivity of the effective interaction strengths S36
1.5 Calculation of total effective interaction energies for the size parameters S38
1.6 Calculation of total effective interaction energies for the energy parameters S44
1.7 Calculation of effective energies of mixing S47

2 Additional results S50

2.1 Density as a function of the Lennard-Jones energy parameters S50
2.2 Thermal expansion coefficients S51
2.3 Energy of mixing diagrams S55

1 Calculation of effective interaction strengths and derived quantities

Effective interaction strengths were calculated as the integral of the density weighted pair interaction potential [1] (Equation 1 of the main manuscript):

$$U_{ab}^{\text{eff}} = 4\pi f_{AB} \frac{N_a N_b}{V} \int r^2 \; U_{ab}(r) \; g_{ab}(r) \; dr \tag{1}$$

with $f_{AB} = \frac{1}{2}$ for auto-interactions and $f_{AB} = 1$ for cross-interactions.

1.1 Effective interaction strengths as a function of the Lennard-Jones size parameters

Table S1: Effective interaction strengths of all pairwise atom-atom interactions in an equimolar hexane-perfluorohexane mixture as a function of the Lennard-Jones size parameter of hydrogen atoms $\sigma(H)$. Calculation according to Equation 1. The atom numbers used are $N_H = N_F = 3500$ and $N_{C_H} = N_{C_F} = 1500$.

parameter	box volume	integral	prefactor	U^{eff}
$\sigma(H)$ in Å	V in Å^3	$\int r^2 g_{HH}(r) U_{HH}(r)$ in Å^3 kcal mol^{-1}	$4\pi N_H N_H / 2V$ in Å^{-3}	U_{HH}^{eff} in kcal mol^{-1}
1.87	123505.99	-0.11	623.20	-68.67
2	124550.54	-0.14	617.97	-85.57
2.12	126203.84	-0.17	609.88	-105.48
2.25	128940.7	-0.2	596.93	-120.44
2.37	131639.19	-0.23	584.70	-136.81
2.5	135242.69	-0.28	569.12	-159.08
2.62	139314.07	-0.31	552.49	-171.66
2.75	144372.63	-0.35	533.13	-188.17
2.87	149721.29	-0.41	514.08	-210.33
3	155634.05	-0.48	494.55	-235.84
3.12	161878.63	-0.53	475.47	-252.99
3.25	169847.38	-0.61	453.17	-276.51
3.37	177599.1	-0.68	433.39	-296.86
3.5	187247.42	-0.79	411.06	-325.64
3.62	197035.77	-0.88	390.63	-342.49
3.75	211708.74	-0.96	363.56	-348.6
$\sigma(H)$ in Å	V in Å^3	$\int r^2 g_{HC_H}(r) U_{HC_H}(r)\; dr$ in Å^3 kcal mol^{-1}	$4\pi N_H N_{C_H} / 2V$ in Å^{-3}	$U_{HC_H}^{\text{eff}}$ in kcal mol^{-1}
1.87	123505.99	-0.61	267.09	-164.05
2	124550.54	-0.71	264.85	-188.36
2.12	126203.84	-0.84	261.38	-219.56
2.25	128940.7	-0.93	255.83	-236.99
2.37	131639.19	-1.02	250.58	-256.46
2.5	135242.69	-1.15	243.91	-281.48
2.62	139314.07	-1.2	236.78	-285.22
2.75	144372.63	-1.26	228.48	-288.41
2.87	149721.29	-1.34	220.32	-295.76

continued on next page

Table S1: Effective interaction strengths as a function of $\sigma(H)$ - Extension.

parameter	box volume	integral	prefactor	U^{eff}
$\sigma(H)$ in Å	V in $Å^3$	$\int r^2 g_{HC_H}(r) U_{HC_H}(r)\ dr$ in $Å^3$ kcal mol^{-1}	$4\pi N_H N_{C_H}/2V$ in $Å^{-3}$	$U^{\text{eff}}_{HC_H}$ in kcal mol^{-1}
3	155634.05	-1.41	211.95	-299.27
3.12	161878.63	-1.42	203.77	-289.94
3.25	169847.38	-1.46	194.21	-283.12
3.37	177599.1	-1.48	185.74	-274.09
3.5	187247.42	-1.52	176.17	-267.14
3.62	197035.77	-1.51	167.41	-253.55
3.75	211708.74	-1.48	155.81	-230.93
$\sigma(H)$ in Å	V in $Å^3$	$\int r^2 g_{C_HC_H}(r) U_{C_HC_H}(r)\ dr$ in $Å^3$ kcal mol^{-1}	$4\pi N_{C_H} N_{C_H}/2V$ in $Å^{-3}$	$U^{\text{eff}}_{C_HC_H}$ in kcal mol^{-1}
1.87	123505.99	-3.02	114.47	-345.20
2	124550.54	-3.16	113.51	-358.61
2.12	126203.84	-3.41	112.02	-382.13
2.25	128940.7	-3.39	109.64	-372.12
2.37	131639.19	-3.39	107.39	-364.21
2.5	135242.69	-3.41	104.53	-356.02
2.62	139314.07	-3.20	101.48	-324.68
2.75	144372.63	-2.98	97.92	-291.94
2.87	149721.29	-2.84	94.42	-268.46
3	155634.05	-2.66	90.84	-241.80
3.12	161878.63	-2.41	87.33	-210.58
3.25	169847.38	-2.20	83.23	-183.28
3.37	177599.1	-2.01	79.60	-160.33
3.5	187247.42	-1.86	75.50	-140.20
3.62	197035.77	-1.69	71.75	-120.91
3.75	211708.74	-1.49	66.78	-99.39
$\sigma(H)$ in Å	V in $Å^3$	$\int r^2 g_{FF}(r) U_{FF}(r)\ dr$ in $Å^3$ kcal mol^{-1}	$4\pi N_F N_F/2V$ in $Å^{-3}$	U^{eff}_{FF} in kcal mol^{-1}
1.87	123505.99	-0.65	623.2	-405.46
2	124550.54	-0.66	617.97	-409.07
2.12	126203.84	-0.69	609.88	-418.36
2.25	128940.7	-0.69	596.93	-413.5
2.37	131639.19	-0.71	584.7	-414.52
2.5	135242.69	-0.74	569.12	-419.28
2.62	139314.07	-0.74	552.49	-407.96
2.75	144372.63	-0.75	533.13	-398.94
2.87	149721.29	-0.77	514.08	-396.31
3	155634.05	-0.8	494.55	-393.38
3.12	161878.63	-0.8	475.47	-381.18
3.25	169847.38	-0.83	453.17	-375.26
3.37	177599.1	-0.85	433.39	-368.8
3.5	187247.42	-0.91	411.06	-373.54
3.62	197035.77	-0.94	390.63	-365.54
3.75	211708.74	-0.96	363.56	-348.34

continued on next page

Table S1: Effective interaction strengths as a function of $\sigma(H)$ - Extension.

parameter	box volume	integral	prefactor	U^{eff}
$\sigma(H)$ in Å	V in Å^3	$\int r^2 g_{FC_F}(r) U_{FC_F}(r)\ dr$ in Å^3 kcal mol^{-1}	$4\pi N_F N_{C_F}/2V$ in Å^{-3}	$U^{\text{eff}}_{FC_F}$ in kcal mol^{-1}
1.87	123505.99	-1.27	267.09	-338.72
2	124550.54	-1.29	264.85	-342.96
2.12	126203.84	-1.34	261.38	-350.47
2.25	128940.7	-1.36	255.83	-346.98
2.37	131639.19	-1.39	250.58	-347.74
2.5	135242.69	-1.44	243.91	-351.23
2.62	139314.07	-1.45	236.78	-343.03
2.75	144372.63	-1.46	228.48	-333.77
2.87	149721.29	-1.5	220.32	-331.16
3	155634.05	-1.55	211.95	-328.31
3.12	161878.63	-1.56	203.77	-318.78
3.25	169847.38	-1.61	194.21	-312.96
3.37	177599.1	-1.66	185.74	-307.79
3.5	187247.42	-1.75	176.17	-309.16
3.62	197035.77	-1.81	167.41	-303.82
3.75	211708.74	-1.85	155.81	-288.6
$\sigma(H)$ in Å	V in Å^3	$\int r^2 g_{C_FC_F}(r) U_{C_FC_F}(r)\ dr$ in Å^3 kcal mol^{-1}	$4\pi N_{C_F} N_{C_F}/2V$ in Å^{-3}	$U^{\text{eff}}_{C_FC_F}$ in kcal mol^{-1}
1.87	123505.99	-1.71	114.47	-195.54
2	124550.54	-1.74	113.51	-198.01
2.12	126203.84	-1.81	112.02	-202.26
2.25	128940.7	-1.83	109.64	-200.25
2.37	131639.19	-1.87	107.39	-200.62
2.5	135242.69	-1.94	104.53	-202.58
2.62	139314.07	-1.95	101.48	-197.84
2.75	144372.63	-1.97	97.92	-192.5
2.87	149721.29	-2.02	94.42	-190.92
3	155634.05	-2.08	90.84	-189.26
3.12	161878.63	-2.1	87.33	-183.79
3.25	169847.38	-2.17	83.23	-180.41
3.37	177599.1	-2.23	79.6	-177.43
3.5	187247.42	-2.36	75.5	-178.07
3.62	197035.77	-2.44	71.75	-175.06
3.75	211708.74	-2.49	66.78	-166.22
$\sigma(H)$ in Å	V in Å^3	$\int r^2 g_{HF}(r) U_{HF}(r)\ dr$ in Å^3 kcal mol^{-1}	$4\pi N_H N_F/V$ in Å^{-3}	U^{eff}_{HF} in kcal mol^{-1}
1.87	123505.99	-0.19	1246.4	-232.51
2	124550.54	-0.2	1235.95	-242.33
2.12	126203.84	-0.2	1219.76	-239.61
2.25	128940.7	-0.21	1193.87	-249.1
2.37	131639.19	-0.22	1169.39	-251.68
2.5	135242.69	-0.22	1138.24	-248.34
2.62	139314.07	-0.24	1104.97	-264.69

continued on next page

Table S1: Effective interaction strengths as a function of $\sigma(H)$ - Extension.

parameter	box volume	integral	prefactor	U^{eff}
$\sigma(H)$ in Å	V in Å^3	$\int r^2 g_{HF}(r) U_{HF}(r)\ dr$ in Å^3 kcal mol^{-1}	$4\pi N_H N_F / V$ in Å^{-3}	U^{eff}_{HF} in kcal mol^{-1}
2.75	144372.63	-0.26	1066.26	-279.83
2.87	149721.29	-0.27	1028.16	-282.03
3	155634.05	-0.29	989.1	-287.77
3.12	161878.63	-0.32	950.95	-304.52
3.25	169847.38	-0.34	906.33	-310.09
3.37	177599.1	-0.36	866.77	-316.04
3.5	187247.42	-0.37	822.11	-306.75
3.62	197035.77	-0.4	781.27	-312.53
3.75	211708.74	-0.44	727.12	-321.21
$\sigma(H)$ in Å	V in Å^3	$\int r^2 g_{FC_H}(r) U_{FC_H}(r)\ dr$ in Å^3 kcal mol^{-1}	$4\pi N_F N_{C_H} / V$ in Å^{-3}	$U^{\text{eff}}_{FC_H}$ in kcal mol^{-1}
1.87	123505.99	-1.18	534.17	-629.76
2	124550.54	-1.16	529.69	-615.65
2.12	126203.84	-1.1	522.75	-574.35
2.25	128940.7	-1.09	511.66	-559.21
2.37	131639.19	-1.05	501.17	-527.92
2.5	135242.69	-0.98	487.82	-479.85
2.62	139314.07	-0.99	473.56	-467.95
2.75	144372.63	-0.98	456.97	-448.18
2.87	149721.29	-0.93	440.64	-408.11
3	155634.05	-0.88	423.9	-372.57
3.12	161878.63	-0.87	407.55	-355.59
3.25	169847.38	-0.83	388.43	-322.24
3.37	177599.1	-0.79	371.47	-294.8
3.5	187247.42	-0.72	352.33	-254.94
3.62	197035.77	-0.7	334.83	-234.08
3.75	211708.74	-0.68	311.62	-213.37
$\sigma(H)$ in Å	V in Å^3	$\int r^2 g_{HC_F}(r) U_{HC_F}(r)\ dr$ in Å^3 kcal mol^{-1}	$4\pi N_H N_{C_F} / V$ in Å^{-3}	$U^{\text{eff}}_{HC_F}$ in kcal mol^{-1}
1.87	123505.99	-0.31	534.17	-166.95
2	124550.54	-0.36	529.69	-188.13
2.12	126203.84	-0.38	522.75	-197.21
2.25	128940.7	-0.42	511.66	-216.33
2.37	131639.19	-0.45	501.17	-227.37
2.5	135242.69	-0.48	487.82	-231.98
2.62	139314.07	-0.53	473.56	-249.25
2.75	144372.63	-0.58	456.97	-266.12
2.87	149721.29	-0.61	440.64	-267.74
3	155634.05	-0.64	423.9	-272.05
3.12	161878.63	-0.7	407.55	-286.64
3.25	169847.38	-0.74	388.43	-288.93
3.37	177599.1	-0.78	371.47	-290.99
3.5	187247.42	-0.79	352.33	-278.92

continued on next page

Table S1: Effective interaction strengths as a function of $\sigma(H)$ - Extension.

parameter	box volume	integral	prefactor	U^{eff}
$\sigma(H)$ in Å	V in Å^3	$\int r^2 g_{HC_F}(r) U_{HC_F}(r)\ dr$ in Å^3 kcal mol^{-1}	$4\pi N_H N_{C_F}/V$ in Å^{-3}	$U^{\text{eff}}_{HC_F}$ in kcal mol^{-1}
3.62	197035.77	-0.84	334.83	-280.96
3.75	211708.74	-0.91	311.62	-283.53
$\sigma(H)$ in Å	V in Å^3	$\int r^2 g_{C_H C_F}(r) U_{C_H C_F}(r)\ dr$ in Å^3 kcal mol^{-1}	$4\pi N_{C_H} N_{C_F}/V$ in Å^{-3}	$U^{\text{eff}}_{C_H C_F}$ in kcal mol^{-1}
1.87	123505.99	-1.9	228.93	-436.06
2	124550.54	-1.82	227.01	-413.45
2.12	126203.84	-1.67	224.04	-374.99
2.25	128940.7	-1.62	219.28	-354.91
2.37	131639.19	-1.53	214.79	-327.65
2.5	135242.69	-1.4	209.06	-291.71
2.62	139314.07	-1.38	202.95	-279.85
2.75	144372.63	-1.35	195.84	-264.49
2.87	149721.29	-1.26	188.85	-238.62
3	155634.05	-1.19	181.67	-216.28
3.12	161878.63	-1.18	174.66	-205.69
3.25	169847.38	-1.12	166.47	-185.87
3.37	177599.1	-1.07	159.2	-169.98
3.5	187247.42	-0.97	151	-147.02
3.62	197035.77	-0.94	143.5	-135.18
3.75	211708.74	-0.92	133.55	-123.49

Table S2: Effective interaction strengths of all pairwise atom-atom interactions in an equimolar hexane-perfluorohexane mixture as a function of the Lennard-Jones size parameter of hexane carbon atoms $\sigma(C_H)$. Calculation according to Equation 1. The atom numbers used are $N_H = N_F = 3500$ and $N_{C_H} = N_{C_F} = 1500$.

parameter	box volume	integral	prefactor	U^{eff}
$\sigma(C_H)$ in Å	V in Å^3	$\int r^2 g_{HH}(r) U_{HH}(r)\ dr$ in Å^3 kcal mol^{-1}	$4\pi N_H N_H/2V$ in Å^{-3}	U^{eff}_{HH} in kcal mol^{-1}
2.63	190506.4	-0.21	404.02	-83.47
2.75	166284.27	-0.21	462.88	-95.65
2.88	155720.87	-0.21	494.28	-103.13
3	149129.95	-0.21	516.12	-108.63
3.13	143795.47	-0.22	535.27	-116.85
3.25	140121.84	-0.23	549.3	-125.73
3.38	137547.91	-0.24	559.58	-136.98
3.5	135242.69	-0.28	569.12	-159.08
3.63	134060.5	-0.3	574.14	-169.52
3.75	133589.56	-0.33	576.16	-189.68
3.88	134060.5	-0.33	574.14	-192.11
4	134445.92	-0.33	572.49	-187.36
4.13	136352.31	-0.31	564.49	-177.3
4.25	137947.99	-0.29	557.96	-162.99
4.38	140040.92	-0.27	549.62	-147.98
4.5	142318.41	-0.25	540.82	-135.61
$\sigma(C_H)$ in Å	V in Å^3	$\int r^2 g_{HC_H}(r) U_{HC_H}(r)\ dr$ in Å^3 kcal mol^{-1}	$4\pi N_H N_{C_H}/2V$ in Å^{-3}	$U^{\text{eff}}_{HC_H}$ in kcal mol^{-1}
2.63	190506.4	-0.42	173.15	-71.93
2.75	166284.27	-0.49	198.38	-97.09
2.88	155720.87	-0.57	211.83	-120.36
3	149129.95	-0.65	221.19	-144.16
3.13	143795.47	-0.75	229.4	-172.81
3.25	140121.84	-0.85	235.41	-200.73
3.38	137547.91	-0.97	239.82	-232.26
3.5	135242.69	-1.15	243.91	-281.48
3.63	134060.5	-1.25	246.06	-307.88
3.75	133589.56	-1.42	246.93	-349.7
3.88	134060.5	-1.45	246.06	-356.02
4	134445.92	-1.43	245.35	-351.94
4.13	136352.31	-1.41	241.92	-341.1
4.25	137947.99	-1.35	239.12	-323.58
4.38	140040.92	-1.31	235.55	-308.35
4.5	142318.41	-1.29	231.78	-297.85
$\sigma(C_H)$ in Å	V in Å^3	$\int r^2 g_{C_HC_H}(r) U_{C_HC_H}(r)\ dr$ in Å^3 kcal mol^{-1}	$4\pi N_{C_H} N_{C_H}/2V$ in Å^{-3}	$U^{\text{eff}}_{C_HC_H}$ in kcal mol^{-1}
2.63	190506.4	-0.52	74.21	-38.66
2.75	166284.27	-0.71	85.02	-60.01
2.88	155720.87	-0.95	90.79	-86.03
3	149129.95	-1.24	94.8	-117.09

continued on next page

Table S2: Effective interaction strengths as a function of $\sigma(C_H)$ - Extension.

parameter	box volume	integral	prefactor	U^{eff}
$\sigma(C_H)$ in Å	V in Å^3	$\int r^2 g_{C_H C_H}(r) U_{C_H C_H}(r)\ dr$ in Å^3 kcal mol^{-1}	$4\pi N_{C_H} N_{C_H}/2V$ in Å^{-3}	$U^{\text{eff}}_{C_H C_H}$ in kcal mol^{-1}
3.13	143795.47	-1.63	98.31	-160.2
3.25	140121.84	-2.06	100.89	-208.19
3.38	137547.91	-2.61	102.78	-268.7
3.5	135242.69	-3.41	104.53	-356.02
3.63	134060.5	-4.03	105.45	-424.9
3.75	133589.56	-4.86	105.83	-514.56
3.88	134060.5	-5.26	105.45	-554.99
4	134445.92	-5.43	105.15	-571.25
4.13	136352.31	-5.54	103.68	-574.29
4.25	137947.99	-5.48	102.48	-561.36
4.38	140040.92	-5.48	100.95	-553.02
4.5	142318.41	-5.57	99.33	-553.02
$\sigma(C_H)$ in Å	V in Å^3	$\int r^2 g_{FF}(r) U_{FF}(r)\ dr$ in Å^3 kcal mol^{-1}	$4\pi N_F N_F/2V$ in Å^{-3}	U^{eff}_{FF} in kcal mol^{-1}
2.63	190506.4	-0.95	404.02	-382.04
2.75	166284.27	-0.79	462.88	-365.84
2.88	155720.87	-0.73	494.28	-361.62
3	149129.95	-0.71	516.12	-366.85
3.13	143795.47	-0.7	535.27	-374.61
3.25	140121.84	-0.7	549.3	-385.62
3.38	137547.91	-0.71	559.58	-394.62
3.5	135242.69	-0.74	569.12	-419.28
3.63	134060.5	-0.74	574.14	-424.31
3.75	133589.56	-0.76	576.16	-439.93
3.88	134060.5	-0.75	574.14	-429.06
4	134445.92	-0.73	572.49	-415.27
4.13	136352.31	-0.7	564.49	-396.38
4.25	137947.99	-0.67	557.96	-372.67
4.38	140040.92	-0.64	549.62	-351.19
4.5	142318.41	-0.62	540.82	-335.26
$\sigma(C_H)$ in Å	V in Å^3	$\int r^2 g_{FC_F}(r) U_{FC_F}(r)\ dr$ in Å^3 kcal mol^{-1}	$4\pi N_F N_{C_F}/2V$ in Å^{-3}	$U^{\text{eff}}_{FC_F}$ in kcal mol^{-1}
2.63	190506.4	-1.83	173.15	-316.42
2.75	166284.27	-1.52	198.38	-301.14
2.88	155720.87	-1.41	211.83	-297.72
3	149129.95	-1.37	221.19	-302.37
3.13	143795.47	-1.36	229.4	-311.96
3.25	140121.84	-1.36	235.41	-321.2
3.38	137547.91	-1.37	239.82	-327.99
3.5	135242.69	-1.44	243.91	-351.23
3.63	134060.5	-1.45	246.06	-356.35
3.75	133589.56	-1.5	246.93	-370.79
3.88	134060.5	-1.47	246.06	-361.89

continued on next page

Table S2: Effective interaction strengths as a function of $\sigma(C_H)$ - Extension.

parameter	box volume	integral	prefactor	U^{eff}
$\sigma(C_H)$ in Å	V in Å^3	$\int r^2 g_{FC_F}(r) U_{FC_F}(r)\ dr$ in Å^3 kcal mol^{-1}	$4\pi N_F N_{C_F}/2V$ in Å^{-3}	$U^{\text{eff}}_{FC_F}$ in kcal mol^{-1}
4	134445.92	-1.43	245.35	-351.02
4.13	136352.31	-1.38	241.92	-335.04
4.25	137947.99	-1.32	239.12	-315.88
4.38	140040.92	-1.26	235.55	-297.61
4.5	142318.41	-1.22	231.78	-283.89
$\sigma(C_H)$ in Å	V in Å^3	$\int r^2 g_{C_FC_F}(r) U_{C_FC_F}(r)\ dr$ in Å^3 kcal mol^{-1}	$4\pi N_{C_F} N_{C_F}/2V$ in Å^{-3}	$U^{\text{eff}}_{C_FC_F}$ in kcal mol^{-1}
2.63	190506.4	-2.46	74.21	-182.48
2.75	166284.27	-2.04	85.02	-173.7
2.88	155720.87	-1.89	90.79	-171.75
3	149129.95	-1.84	94.8	-174.49
3.13	143795.47	-1.83	98.31	-180.14
3.25	140121.84	-1.84	100.89	-185.37
3.38	137547.91	-1.84	102.78	-189.2
3.5	135242.69	-1.94	104.53	-202.58
3.63	134060.5	-1.95	105.45	-205.53
3.75	133589.56	-2.02	105.83	-213.88
3.88	134060.5	-1.98	105.45	-208.73
4	134445.92	-1.93	105.15	-202.51
4.13	136352.31	-1.86	103.68	-193.32
4.25	137947.99	-1.78	102.48	-182.37
4.38	140040.92	-1.7	100.95	-171.9
4.5	142318.41	-1.65	99.33	-164.09
$\sigma(C_H)$ in Å	V in Å^3	$\int r^2 g_{HF}(r) U_{HF}(r)\ dr$ in Å^3 kcal mol^{-1}	$4\pi N_H N_F/V$ in Å^{-3}	U^{eff}_{HF} in kcal mol^{-1}
2.63	190506.4	-0.2	808.05	-161.54
2.75	166284.27	-0.22	925.75	-205.89
2.88	155720.87	-0.24	988.55	-234.89
3	149129.95	-0.24	1032.24	-248.65
3.13	143795.47	-0.24	1070.53	-258.7
3.25	140121.84	-0.24	1098.6	-265.08
3.38	137547.91	-0.24	1119.16	-267.96
3.5	135242.69	-0.22	1138.24	-248.34
3.63	134060.5	-0.22	1148.27	-255.13
3.75	133589.56	-0.21	1152.32	-238.73
3.88	134060.5	-0.23	1148.27	-260.99
4	134445.92	-0.25	1144.98	-285.11
4.13	136352.31	-0.28	1128.97	-312.96
4.25	137947.99	-0.31	1115.91	-348.11
4.38	140040.92	-0.34	1099.24	-376
4.5	142318.41	-0.36	1081.65	-394.6

continued on next page

Table S2: Effective interaction strengths as a function of $\sigma(C_H)$ - Extension.

parameter	box volume	integral	prefactor	U^{eff}
$\sigma(C_H)$ in Å	V in Å^3	$\int r^2 g_{FC_H}(r) U_{FC_H}(r)\ dr$ in Å^3 kcal mol^{-1}	$4\pi N_F N_{C_H}/V$ in Å^{-3}	$U^{\text{eff}}_{FC_H}$ in kcal mol^{-1}
2.63	190506.4	-0.43	346.31	-148.72
2.75	166284.27	-0.55	396.75	-217.43
2.88	155720.87	-0.67	423.66	-283.3
3	149129.95	-0.69	442.39	-307.45
3.13	143795.47	-0.86	458.8	-396.11
3.25	140121.84	-0.94	470.83	-441.79
3.38	137547.91	-1.01	479.64	-486.48
3.5	135242.69	-0.98	487.82	-479.85
3.63	134060.5	-1.06	492.12	-519.61
3.75	133589.56	-1.03	493.85	-509.13
3.88	134060.5	-1.16	492.12	-572.99
4	134445.92	-1.32	490.71	-647.59
4.13	136352.31	-1.5	483.85	-724.92
4.25	137947.99	-1.71	478.25	-818.5
4.38	140040.92	-1.91	471.1	-901.87
4.5	142318.41	-2.07	463.56	-961.75
$\sigma(C_H)$ in Å	V in Å^3	$\int r^2 g_{HC_F}(r) U_{HC_F}(r)\ dr$ in Å^3 kcal mol^{-1}	$4\pi N_H N_{C_F}/V$ in Å^{-3}	$U^{\text{eff}}_{HC_F}$ in kcal mol^{-1}
2.63	190506.4	-0.41	346.31	-140.76
2.75	166284.27	-0.46	396.75	-182.96
2.88	155720.87	-0.5	423.66	-211.84
3	149129.95	-0.52	442.39	-229.74
3.13	143795.47	-0.53	458.8	-242.95
3.25	140121.84	-0.53	470.83	-249.38
3.38	137547.91	-0.53	479.64	-252.2
3.5	135242.69	-0.48	487.82	-231.98
3.63	134060.5	-0.48	492.12	-233.87
3.75	133589.56	-0.44	493.85	-215.8
3.88	134060.5	-0.46	492.12	-228.2
4	134445.92	-0.5	490.71	-244.51
4.13	136352.31	-0.54	483.85	-259.17
4.25	137947.99	-0.58	478.25	-278.2
4.38	140040.92	-0.62	471.1	-291.63
4.5	142318.41	-0.64	463.56	-295.99
$\sigma(C_H)$ in Å	V in Å^3	$\int r^2 g_{C_H C_F}(r) U_{C_H C_F}(r)\ dr$ in Å^3 kcal mol^{-1}	$4\pi N_{C_H} N_{C_F}/V$ in Å^{-3}	$U^{\text{eff}}_{C_H C_F}$ in kcal mol^{-1}
2.63	190506.4	-0.54	148.42	-80.62
2.75	166284.27	-0.7	170.04	-118.77
2.88	155720.87	-0.86	181.57	-156.71
3	149129.95	-1	189.6	-190.29
3.13	143795.47	-1.15	196.63	-226.29
3.25	140121.84	-1.27	201.78	-257.25
3.38	137547.91	-1.4	205.56	-288.74

continued on next page

Table S2: Effective interaction strengths as a function of $\sigma(C_H)$ - Extension.

parameter	box volume	integral	prefactor	U^{eff}
$\sigma(C_H)$ in Å	V in $Å^3$	$\int r^2 g_{C_H C_F}(r) U_{C_H C_F}(r)\ dr$ in $Å^3$ kcal mol^{-1}	$4\pi N_{C_H} N_{C_F}/V$ in $Å^{-3}$	$U^{\mathrm{eff}}_{C_H C_F}$ in kcal mol^{-1}
3.5	135242.69	-1.4	209.06	-291.71
3.63	134060.5	-1.53	210.91	-323.37
3.75	133589.56	-1.54	211.65	-324.97
3.88	134060.5	-1.78	210.91	-374.68
4	134445.92	-2.06	210.3	-434.03
4.13	136352.31	-2.4	207.36	-497.87
4.25	137947.99	-2.8	204.96	-574.54
4.38	140040.92	-3.21	201.9	-648.45
4.5	142318.41	-1.53	198.67	-303.43

Table S3: Effective interaction strengths of all pairwise atom-atom interactions in an equimolar hexane-perfluorohexane mixture as a function of the Lennard-Jones size parameter of fluorine atoms $\sigma(F)$. Calculation according to Equation 1. The atom numbers used are $N_H = N_F = 3500$ and $N_{C_H} = N_{C_F} = 1500$.

parameter	box volume	integral	prefactor	U^{eff}
$\sigma(F)$ in Å	V in $Å^3$	$\int r^2 g_{HH}(r) U_{HH}(r)\, dr$ in $Å^3$ kcal mol^{-1}	$4\pi N_H N_H / 2V$ in Å^{-3}	U^{eff}_{HH} in kcal mol^{-1}
2.7	124326.21	-0.26	619.09	-161.84
2.95	135242.69	-0.28	569.12	-159.08
3.08	148792.75	-0.3	517.29	-153.47
3.2	156852.43	-0.29	490.71	-142.05
3.33	164566.59	-0.31	467.71	-143.01
3.45	174958.26	-0.31	439.93	-134.32
3.58	185388.01	-0.32	415.18	-133.97
3.7	185388.01	-0.35	415.18	-145.11
3.95	189514.87	-0.39	406.14	-160.42
4.2	253756.36	-0.5	303.32	-151.72
$\sigma(F)$ in Å	V in $Å^3$	$\int r^2 g_{HC_H}(r) U_{HC_H}(r)\, dr$ in $Å^3$ kcal mol^{-1}	$4\pi N_H N_{C_H} / 2V$ in Å^{-3}	$U^{\text{eff}}_{HC_H}$ in kcal mol^{-1}
2.7	124326.21	-1.08	265.32	-287.27
2.95	135242.69	-1.15	243.91	-281.48
3.08	148792.75	-1.23	221.7	-272.52
3.2	156852.43	-1.2	210.3	-252.07
3.33	164566.59	-1.27	200.45	-253.76
3.45	174958.26	-1.26	188.54	-237.41
3.58	185388.01	-1.34	177.93	-238.41
3.7	185388.01	-1.44	177.93	-256.24
3.95	189514.87	-1.63	174.06	-283.8
4.2	253756.36	-2.06	129.99	-267.5
$\sigma(F)$ in Å	V in $Å^3$	$\int r^2 g_{C_HC_H}(r) U_{C_HC_H}(r)\, dr$ in $Å^3$ kcal mol^{-1}	$4\pi N_{C_H} N_{C_H} / 2V$ in Å^{-3}	$U^{\text{eff}}_{C_HC_H}$ in kcal mol^{-1}
2.7	124326.21	-3.2	113.71	-363.58
2.95	135242.69	-3.41	104.53	-356.02
3.08	148792.75	-3.63	95.01	-345.12
3.2	156852.43	-3.54	90.13	-318.8
3.33	164566.59	-3.74	85.91	-320.91
3.45	174958.26	-3.71	80.8	-300.11
3.58	185388.01	-3.95	76.26	-301.53
3.7	185388.01	-4.25	76.26	-323.83
3.95	189514.87	-4.81	74.6	-358.65
4.2	253756.36	-6.07	55.71	-338.03
$\sigma(F)$ in Å	V in $Å^3$	$\int r^2 g_{FF}(r) U_{FF}(r)\, dr$ in $Å^3$ kcal mol^{-1}	$4\pi N_F N_F / 2V$ in Å^{-3}	U^{eff}_{FF} in kcal mol^{-1}
2.7	124326.21	-0.54	619.09	-336.5
2.95	135242.69	-0.74	569.12	-419.28
3.08	148792.75	-0.86	517.29	-445.99
3.2	156852.43	-0.95	490.71	-468.43

continued on next page

Table S3: Effective interaction strengths as a function of $\sigma(F)$ - Extension.

parameter	box volume	integral	prefactor	U^{eff}
$\sigma(F)$ in Å	V in Å^3	$\int r^2 g_{FF}(r) U_{FF}(r)\ dr$ in Å^3 kcal mol^{-1}	$4\pi N_F N_F / 2V$ in Å^{-3}	U^{eff}_{FF} in kcal mol^{-1}
3.33	164566.59	-1.09	467.71	-511.47
3.45	174958.26	-1.21	439.93	-531.88
3.58	185388.01	-1.37	415.18	-569.51
3.7	185388.01	-1.53	415.18	-637.08
3.95	189514.87	-1.88	406.14	-764.72
4.2	253756.36	-2.27	303.32	-688.19
$\sigma(F)$ in Å	V in Å^3	$\int r^2 g_{FC_F}(r) U_{FC_F}(r)$ in Å^3 kcal mol^{-1}	$4\pi N_F N_{C_F} / 2V$ in Å^{-3}	$U^{\text{eff}}_{FC_F}$ in kcal mol^{-1}
2.7	124326.21	-1.3	265.32	-345.1
2.95	135242.69	-1.44	243.91	-351.23
3.08	148792.75	-1.51	221.7	-335.43
3.2	156852.43	-1.52	210.3	-318.86
3.33	164566.59	-1.56	200.45	-312.16
3.45	174958.26	-1.56	188.54	-293.9
3.58	185388.01	-1.59	177.93	-282.29
3.7	185388.01	-1.61	177.93	-286.16
3.95	189514.87	-1.62	174.06	-282.05
4.2	253756.36	-1.61	129.99	-209.53
$\sigma(F)$ in Å	V in Å^3	$\int r^2 g_{C_F C_F}(r) U_{C_F C_F}(r)\ dr$ in Å^3 kcal mol^{-1}	$4\pi N_{C_F} N_{C_F} / 2V$ in Å^{-3}	$U^{\text{eff}}_{C_F C_F}$ in kcal mol^{-1}
2.7	124326.21	-2.21	113.71	-250.83
2.95	135242.69	-1.94	104.53	-202.58
3.08	148792.75	-1.82	95.01	-172.53
3.2	156852.43	-1.64	90.13	-147.7
3.33	164566.59	-1.51	85.91	-129.73
3.45	174958.26	-1.37	80.8	-110.76
3.58	185388.01	-1.26	76.26	-95.98
3.7	185388.01	-1.16	76.26	-88.73
3.95	189514.87	-0.98	74.6	-72.89
4.2	253756.36	-0.82	55.71	-45.58
$\sigma(F)$ in Å	V in Å^3	$\int r^2 g_{HF}(r) U_{HF}(r)$ in Å^3 kcal mol^{-1}	$4\pi N_H N_F / V$ in Å^{-3}	U^{eff}_{HF} in kcal mol^{-1}
2.7	124326.21	-0.19	1238.18	-230.61
2.95	135242.69	-0.22	1138.24	-248.34
3.08	148792.75	-0.23	1034.58	-238.41
3.2	156852.43	-0.27	981.42	-260.78
3.33	164566.59	-0.28	935.41	-264.34
3.45	174958.26	-0.32	879.86	-277.29
3.58	185388.01	-0.33	830.36	-276.04
3.7	185388.01	-0.35	830.36	-287.5
3.95	189514.87	-0.39	812.27	-315.77
4.2	253756.36	-0.42	606.64	-253.23

continued on next page

Table S3: Effective interaction strengths as a function of $\sigma(F)$ - Extension.

parameter	box volume	integral	prefactor	U^{eff}
$\sigma(F)$ in Å	V in Å^3	$\int r^2 g_{FC_H}(r) U_{FC_H}(r)\ dr$ in Å^3 kcal mol^{-1}	$4\pi N_F N_{C_H}/V$ in Å^{-3}	$U^{\text{eff}}_{FC_H}$ in kcal mol^{-1}
2.7	124326.21	-0.87	530.65	-460.19
2.95	135242.69	-0.98	487.82	-479.85
3.08	148792.75	-1.01	443.39	-449.14
3.2	156852.43	-1.15	420.61	-484.3
3.33	164566.59	-1.2	400.89	-480.83
3.45	174958.26	-1.32	377.08	-499.6
3.58	185388.01	-1.37	355.87	-488.01
3.7	185388.01	-1.41	355.87	-502.04
3.95	189514.87	-1.55	348.12	-537.92
4.2	253756.36	-1.62	259.99	-421.44
$\sigma(F)$ in Å	V in Å^3	$\int r^2 g_{HC_F}(r) U_{HC_F}(r)\ dr$ in Å^3 kcal mol^{-1}	$4\pi N_H N_{C_F}/V$ in Å^{-3}	$U^{\text{eff}}_{HC_F}$ in kcal mol^{-1}
2.7	124326.21	-0.52	530.65	-274.49
2.95	135242.69	-0.48	487.82	-231.98
3.08	148792.75	-0.44	443.39	-194.73
3.2	156852.43	-0.45	420.61	-190.18
3.33	164566.59	-0.42	400.89	-169.9
3.45	174958.26	-0.43	377.08	-160.78
3.58	185388.01	-0.4	355.87	-141.62
3.7	185388.01	-0.37	355.87	-132.91
3.95	189514.87	-0.34	348.12	-118.47
4.2	253756.36	-0.3	259.99	-77.75
$\sigma(F)$ in Å	V in Å^3	$\int r^2 g_{C_HC_F}(r) U_{C_HC_F}(r)\ dr$ in Å^3 kcal mol^{-1}	$4\pi N_{C_H} N_{C_F}/V$ in Å^{-3}	$U^{\text{eff}}_{C_HC_F}$ in kcal mol^{-1}
2.7	124326.21	-1.51	227.42	-344.01
2.95	135242.69	-1.4	209.06	-291.71
3.08	148792.75	-1.29	190.02	-245.93
3.2	156852.43	-1.34	180.26	-241
3.33	164566.59	-1.26	171.81	-216.37
3.45	174958.26	-1.27	161.61	-205.63
3.58	185388.01	-1.19	152.51	-182.25
3.7	185388.01	-1.13	152.51	-172.08
3.95	189514.87	-1.04	149.19	-155.11
4.2	253756.36	-0.92	111.42	-102.99

Table S4: Effective interaction strengths of all pairwise atom-atom interactions in an equimolar hexane-perfluorohexane mixture as a function of the Lennard-Jones size parameter of pefluorohexane carbon atoms $\sigma(C_F)$. Calculation according to Equation 1. The atom numbers used are $N_H = N_F = 3500$ and $N_{C_H} = N_{C_F} = 1500$.

parameter	box volume	integral	prefactor	U^{eff}
$\sigma(C_F)$ in Å	V in Å^3	$\int r^2 g_{HH}(r)U_{HH}(r)\ dr$ in Å^3 kcal mol^{-1}	$4\pi N_H N_H/2V$ in Å^{-3}	U^{eff}_{HH} in kcal mol^{-1}
2.75	198767.72	-0.4	387.23	-154.03
2.87	177504.33	-0.34	433.62	-149.53
3	164746.84	-0.29	467.2	-136.47
3.13	156765.2	-0.28	490.98	-136.21
3.25	151334.23	-0.26	508.6	-131.56
3.37	146529.88	-0.25	525.28	-129.54
3.5	142727.68	-0.24	539.27	-129.2
3.62	139556.07	-0.24	551.53	-131.03
3.75	137068.84	-0.24	561.54	-135.57
3.87	135163.66	-0.24	569.45	-134.35
4	133432.83	-0.23	576.84	-135.21
4.12	132651	-0.24	580.24	-137.91
4.25	131639.19	-0.24	584.7	-137.46
4.37	131639.19	-0.23	584.7	-136.15
4.5	131949.97	-0.22	583.32	-131.12
4.62	132572.99	-0.22	580.58	-125.61
$\sigma(C_F)$ in Å	V in Å^3	$\int r^2 g_{HC_H}(r)U_{HC_H}(r)\ dr$ in Å^3 kcal mol^{-1}	$4\pi N_H N_{C_H}/2V$ in Å^{-3}	$U^{\text{eff}}_{HC_H}$ in kcal mol^{-1}
2.75	198767.72	-1.62	165.96	-269.59
2.87	177504.33	-1.41	185.84	-261.74
3	164746.84	-1.2	200.23	-239.89
3.13	156765.2	-1.14	210.42	-240.73
3.25	151334.23	-1.06	217.97	-232.08
3.37	146529.88	-1.02	225.12	-229.42
3.5	142727.68	-0.99	231.12	-228.3
3.62	139556.07	-0.99	236.37	-234.15
3.75	137068.84	-1	240.66	-240.01
3.87	135163.66	-0.99	244.05	-241.16
4	133432.83	-0.98	247.22	-241.18
4.12	132651	-0.99	248.67	-246.03
4.25	131639.19	-0.99	250.58	-248.22
4.37	131639.19	-0.98	250.58	-245.19
4.5	131949.97	-0.94	249.99	-236.07
4.62	132572.99	-0.92	248.82	-227.93
$\sigma(C_F)$ in Å	V in Å^3	$\int r^2 g_{C_H C_H}(r)U_{C_H C_H}(r)\ dr$ in Å^3 kcal mol^{-1}	$4\pi N_{C_H} N_{C_H}/2V$ in Å^{-3}	$U^{\text{eff}}_{C_H C_H}$ in kcal mol^{-1}
2.75	198767.72	-4.79	71.12	-341.03
2.87	177504.33	-4.16	79.64	-331.14
3	164746.84	-3.54	85.81	-303.64
3.13	156765.2	-3.38	90.18	-304.59

continued on next page

Table S4: Effective interaction strengths as a function of $\sigma(C_F)$ - Extension.

parameter	box volume	integral	prefactor	U^{eff}
$\sigma(C_F)$ in Å	V in Å^3	$\int r^2 g_{C_H C_H}(r) U_{C_H C_H}(r)\ dr$ in Å^3 kcal mol^{-1}	$4\pi N_{C_H} N_{C_H}/2V$ in Å^{-3}	$U^{\mathrm{eff}}_{C_H C_H}$ in kcal mol^{-1}
3.25	151334.23	-3.15	93.42	-293.83
3.37	146529.88	-3.01	96.48	-290.63
3.5	142727.68	-2.92	99.05	-288.92
3.62	139556.07	-2.92	101.3	-296.23
3.75	137068.84	-2.94	103.14	-303.71
3.87	135163.66	-2.92	104.59	-305.34
4	133432.83	-2.88	105.95	-305.25
4.12	132651	-2.92	106.57	-311.43
4.25	131639.19	-2.93	107.39	-314.17
4.37	131639.19	-2.89	107.39	-310.31
4.5	131949.97	-2.79	107.14	-298.83
4.62	132572.99	-2.71	106.64	-288.5
$\sigma(C_F)$ in Å	V in Å^3	$\int r^2 g_{FF}(r) U_{FF}(r)\ dr$ in Å^3 kcal mol^{-1}	$4\pi N_F N_F/2V$ in Å^{-3}	U^{eff}_{FF} in kcal mol^{-1}
2.75	198767.72	-0.65	387.23	-250
2.87	177504.33	-0.66	433.62	-286.53
3	164746.84	-0.65	467.2	-304.23
3.13	156765.2	-0.66	490.98	-325.27
3.25	151334.23	-0.66	508.6	-337.16
3.37	146529.88	-0.66	525.28	-348.31
3.5	142727.68	-0.67	539.27	-359.12
3.62	139556.07	-0.68	551.53	-373.74
3.75	137068.84	-0.69	561.54	-388.94
3.87	135163.66	-0.7	569.45	-399
4	133432.83	-0.71	576.84	-409.47
4.12	132651	-0.73	580.24	-425.44
4.25	131639.19	-0.75	584.7	-437.13
4.37	131639.19	-0.76	584.7	-442.83
4.5	131949.97	-0.76	583.32	-441.45
4.62	132572.99	-0.76	580.58	-439.05
$\sigma(C_F)$ in Å	V in Å^3	$\int r^2 g_{FC_F}(r) U_{FC_F}(r)\ dr$ in Å^3 kcal mol^{-1}	$4\pi N_F N_{C_F}/2V$ in Å^{-3}	$U^{\mathrm{eff}}_{FC_F}$ in kcal mol^{-1}
2.75	198767.72	-0.58	165.96	-96.99
2.87	177504.33	-0.69	185.84	-128.19
3	164746.84	-0.79	200.23	-157.34
3.13	156765.2	-0.92	210.42	-193.17
3.25	151334.23	-1.03	217.97	-225.42
3.37	146529.88	-1.15	225.12	-259.97
3.5	142727.68	-1.3	231.12	-299.65
3.62	139556.07	-1.45	236.37	-343.57
3.75	137068.84	-1.62	240.66	-390.85
3.87	135163.66	-1.76	244.05	-430.23
4	133432.83	-1.88	247.22	-465.63

continued on next page

Table S4: Effective interaction strengths as a function of $\sigma(C_F)$ - Extension.

parameter	box volume	integral	prefactor	U^{eff}
$\sigma(C_F)$ in Å	V in Å^3	$\int r^2 g_{FC_F}(r) U_{FC_F}(r)\ dr$ in Å^3 kcal mol^{-1}	$4\pi N_F N_{C_F}/2V$ in Å^{-3}	$U^{\text{eff}}_{FC_F}$ in kcal mol^{-1}
4.12	132651	-2.06	248.67	-513.3
4.25	131639.19	-2.2	250.58	-552.39
4.37	131639.19	-2.3	250.58	-577.05
4.5	131949.97	-2.36	249.99	-590.72
4.62	132572.99	-2.42	248.82	-602.23
$\sigma(C_F)$ in Å	V in Å^3	$\int r^2 g_{C_FC_F}(r) U_{C_FC_F}(r)\ dr$ in Å^3 kcal mol^{-1}	$4\pi N_{C_F} N_{C_F}/2V$ in Å^{-3}	$U^{\text{eff}}_{C_FC_F}$ in kcal mol^{-1}
2.75	198767.72	-0.37	71.12	-26.39
2.87	177504.33	-0.5	79.64	-39.73
3	164746.84	-0.65	85.81	-55.96
3.13	156765.2	-0.87	90.18	-78.4
3.25	151334.23	-1.1	93.42	-103.03
3.37	146529.88	-1.38	96.48	-133.11
3.5	142727.68	-1.74	99.05	-172.8
3.62	139556.07	-2.17	101.3	-219.82
3.75	137068.84	-2.7	103.14	-278.74
3.87	135163.66	-3.22	104.59	-336.82
4	133432.83	-3.83	105.95	-405.55
4.12	132651	-4.49	106.57	-478.48
4.25	131639.19	-5.18	107.39	-555.92
4.37	131639.19	-5.76	107.39	-618.84
4.5	131949.97	-6.27	107.14	-671.45
4.62	132572.99	-6.71	106.64	-715.88
$\sigma(C_F)$ in Å	V in Å^3	$\int r^2 g_{HF}(r) U_{HF}(r)\ dr$ in Å^3 kcal mol^{-1}	$4\pi N_H N_F/V$ in Å^{-3}	U^{eff}_{HF} in kcal mol^{-1}
2.75	198767.72	-0.19	774.46	-147.83
2.87	177504.33	-0.2	867.24	-171.19
3	164746.84	-0.22	934.39	-208.61
3.13	156765.2	-0.22	981.97	-219.87
3.25	151334.23	-0.23	1017.21	-237.94
3.37	146529.88	-0.24	1050.56	-253.8
3.5	142727.68	-0.24	1078.54	-263.35
3.62	139556.07	-0.24	1103.06	-266.46
3.75	137068.84	-0.24	1123.07	-267.93
3.87	135163.66	-0.24	1138.9	-273.55
4	133432.83	-0.24	1153.67	-280.65
4.12	132651	-0.24	1160.47	-276.69
4.25	131639.19	-0.24	1169.39	-283.42
4.37	131639.19	-0.25	1169.39	-290.8
4.5	131949.97	-0.26	1166.64	-305.15
4.62	132572.99	-0.27	1161.16	-317.39

continued on next page

Table S4: Effective interaction strengths as a function of $\sigma(C_F)$ - Extension.

parameter	box volume	integral	prefactor	U^{eff}
$\sigma(C_F)$ in Å	V in Å^3	$\int r^2 g_{FC_H}(r) U_{FC_H}(r)\ dr$ in Å^3 kcal mol^{-1}	$4\pi N_F N_{C_H}/V$ in Å^{-3}	$U^{\text{eff}}_{FC_H}$ in kcal mol^{-1}
2.75	198767.72	-0.79	331.91	-260.85
2.87	177504.33	-0.82	371.67	-306.48
3	164746.84	-0.94	400.45	-377.73
3.13	156765.2	-0.96	420.84	-404.88
3.25	151334.23	-1.02	435.95	-445.23
3.37	146529.88	-1.07	450.24	-481.85
3.5	142727.68	-1.1	462.23	-508.07
3.62	139556.07	-1.1	472.74	-518.48
3.75	137068.84	-1.09	481.32	-526.69
3.87	135163.66	-1.11	488.1	-541.02
4	133432.83	-1.13	494.43	-558.73
4.12	132651	-1.11	497.35	-552.84
4.25	131639.19	-1.12	501.17	-562.24
4.37	131639.19	-1.14	501.17	-572.66
4.5	131949.97	-1.2	499.99	-598.93
4.62	132572.99	-1.24	497.64	-617.49
$\sigma(C_F)$ in Å	V in Å^3	$\int r^2 g_{HC_F}(r) U_{HC_F}(r)\ dr$ in Å^3 kcal mol^{-1}	$4\pi N_H N_{C_F}/V$ in Å^{-3}	$U^{\text{eff}}_{HC_F}$ in kcal mol^{-1}
2.75	198767.72	-0.19	331.91	-63.39
2.87	177504.33	-0.23	371.67	-84.28
3	164746.84	-0.3	400.45	-118.48
3.13	156765.2	-0.34	420.84	-143.08
3.25	151334.23	-0.4	435.95	-174.97
3.37	146529.88	-0.47	450.24	-209.5
3.5	142727.68	-0.53	462.23	-245.51
3.62	139556.07	-0.58	472.74	-272.38
3.75	137068.84	-0.63	481.32	-302.81
3.87	135163.66	-0.69	488.1	-336.21
4	133432.83	-0.76	494.43	-374.88
4.12	132651	-0.8	497.35	-396.67
4.25	131639.19	-0.86	501.17	-431.65
4.37	131639.19	-0.93	501.17	-466.21
4.5	131949.97	-1.03	499.99	-517.03
4.62	132572.99	-1.13	497.64	-561.37
$\sigma(C_F)$ in Å	V in Å^3	$\int r^2 g_{C_HC_F}(r) U_{C_HC_F}(r)\ dr$ in Å^3 kcal mol^{-1}	$4\pi N_{C_H} N_{C_F}/V$ in Å^{-3}	$U^{\text{eff}}_{C_HC_F}$ in kcal mol^{-1}
2.75	198767.72	-0.56	142.25	-80
2.87	177504.33	-0.67	159.29	-106.39
3	164746.84	-0.87	171.62	-149.01
3.13	156765.2	-1	180.36	-180.34
3.25	151334.23	-1.18	186.83	-220.66
3.37	146529.88	-1.37	192.96	-263.99
3.5	142727.68	-1.56	198.1	-308.62

continued on next page

Table S4: Effective interaction strengths as a function of $\sigma(C_F)$ - Extension.

parameter	box volume	integral	prefactor	U^{eff}
$\sigma(C_F)$ in Å	V in Å^3	$\int r^2 g_{C_H C_F}(r) U_{C_H C_F}(r)\ dr$ in Å^3 kcal mol^{-1}	$4\pi N_{C_H} N_{C_F}/V$ in Å^{-3}	$U^{\text{eff}}_{C_H C_F}$ in kcal mol^{-1}
3.62	139556.07	-1.7	202.6	-344.2
3.75	137068.84	-1.86	206.28	-383.76
3.87	135163.66	-2.04	209.19	-427.17
4	133432.83	-2.26	211.9	-478.49
4.12	132651	-2.38	213.15	-507.5
4.25	131639.19	-2.58	214.79	-554.57
4.37	131639.19	-2.8	214.79	-601.11
4.5	131949.97	-3.12	214.28	-668.93
4.62	132572.99	-3.42	213.27	-728.42

1.2 Effective interaction strengths as a function of the Lennard-Jones energy parameters

Table S5: Effective interaction strengths of all pairwise atom-atom interactions in an equimolar hexane-perfluorohexane mixture as a function of the Lennard-Jones energy parameter of hydrogen atoms $\varepsilon(H)$. Calculation according to Equation 1. The atom numbers used are $N_H = N_F = 3500$ and $N_{C_H} = N_{C_F} = 1500$.

parameter	box volume	integral	prefactor	U^{eff}
$\varepsilon(H)$ in kcal mol-1	V in Å^3	$\int r^2 g_{HH}(r) U_{HH}(r)\, dr$ in Å^3 kcal mol-1	$4\pi N_H N_H / 2V$ in Å-3	U^{eff}_{HH} in kcal mol-1
0.015	154422	-0.07	498.43	-34.92
0.023	157901.81	-0.14	487.45	-70.36
0.03	151589.95	-0.23	507.74	-115.85
0.038	146529.88	-0.34	525.28	-180.55
0.045	143301.98	-0.5	537.11	-266.99
0.053	140689.14	-0.74	547.09	-405.57
0.06	138991.83	-0.92	553.77	-509.4
$\varepsilon(H)$ in kcal mol-1	V in Å^3	$\int r^2 g_{HC_H}(r) U_{HC_H}(r)\, dr$ in Å^3 kcal mol-1	$4\pi N_H N_{C_H} / 2V$ in Å-3	$U^{\text{eff}}_{HC_H}$ in kcal mol-1
0.015	154422	-0.56	213.61	-118.64
0.023	157901.81	-0.76	208.91	-157.79
0.03	151589.95	-0.94	217.6	-205.13
0.038	146529.88	-1.19	225.12	-268.4
0.045	143301.98	-1.53	230.19	-351.09
0.053	140689.14	-2.04	234.47	-477.45
0.06	138991.83	-2.32	237.33	-551.37
$\varepsilon(H)$ in kcal mol-1	V in Å^3	$\int r^2 g_{C_H C_H}(r) U_{C_H C_H}(r)\, dr$ in Å^3 kcal mol-1	$4\pi N_{C_H} N_{C_H} / 2V$ in Å-3	$U^{\text{eff}}_{C_H C_H}$ in kcal mol-1
0.015	154422	-2.39	91.55	-218.64
0.023	157901.81	-2.54	89.53	-227.84
0.03	151589.95	-2.79	93.26	-260.06
0.038	146529.88	-3.08	96.48	-297.38
0.045	143301.98	-3.63	98.65	-358.51
0.053	140689.14	-4.42	100.49	-444.38
0.06	138991.83	-4.71	101.71	-479.19
$\varepsilon(H)$ in kcal mol-1	V in Å^3	$\int r^2 g_{FF}(r) U_{FF}(r)\, dr$ in Å^3 kcal mol-1	$4\pi N_F N_F / 2V$ in Å-3	U^{eff}_{FF} in kcal mol-1
0.015	154422	-0.62	498.43	-311.01
0.023	157901.81	-0.62	487.45	-303.22
0.03	151589.95	-0.63	507.74	-322.19
0.038	146529.88	-0.65	525.28	-342.76
0.045	143301.98	-0.7	537.11	-375.81
0.053	140689.14	-0.76	547.09	-416.45
0.06	138991.83	-0.78	553.77	-431.01

continued on next page

Table S5: Effective interaction strengths as a function of $\varepsilon(H)$ - Extension.

parameter	box volume	integral	prefactor	U^{eff}
$\varepsilon(H)$ in kcal mol^{-1}	V in Å^3	$\int r^2 g_{FC_F}(r) U_{FC_F}(r)\ dr$ in Å^3 kcal mol^{-1}	$4\pi N_F N_{C_F}/2V$ in Å^{-3}	$U^{\text{eff}}_{FC_F}$ in kcal mol^{-1}
0.015	154422	-1.19	213.61	-254.61
0.023	157901.81	-1.19	208.91	-249.23
0.03	151589.95	-1.23	217.6	-267.23
0.038	146529.88	-1.27	225.12	-284.99
0.045	143301.98	-1.37	230.19	-315.14
0.053	140689.14	-1.48	234.47	-347.93
0.06	138991.83	-1.52	237.33	-360.84
$\varepsilon(H)$ in kcal mol^{-1}	V in Å^3	$\int r^2 g_{C_FC_F}(r) U_{C_FC_F}(r)\ dr$ in Å^3 kcal mol^{-1}	$4\pi N_{C_F} N_{C_F}/2V$ in Å^{-3}	$U^{\text{eff}}_{C_FC_F}$ in kcal mol^{-1}
0.015	154422	-1.6	91.55	-146.92
0.023	157901.81	-1.61	89.53	-143.78
0.03	151589.95	-1.65	93.26	-154.1
0.038	146529.88	-1.7	96.48	-164.24
0.045	143301.98	-1.84	98.65	-181.56
0.053	140689.14	-1.99	100.49	-200.39
0.06	138991.83	-2.04	101.71	-207.79
$\varepsilon(H)$ in kcal mol^{-1}	V in Å^3	$\int r^2 g_{HF}(r) U_{HF}(r)\ dr$ in Å^3 kcal mol^{-1}	$4\pi N_H N_F/V$ in Å^{-3}	U^{eff}_{HF} in kcal mol^{-1}
0.015	154422	-0.16	996.87	-161.42
0.023	157901.81	-0.21	974.9	-207.9
0.03	151589.95	-0.24	1015.49	-246.8
0.038	146529.88	-0.27	1050.56	-282.39
0.045	143301.98	-0.25	1074.22	-268.3
0.053	140689.14	-0.2	1094.17	-224.27
0.06	138991.83	-0.2	1107.53	-222.19
$\varepsilon(H)$ in kcal mol^{-1}	V in Å^3	$\int r^2 g_{FC_H}(r) U_{FC_H}(r)\ dr$ in Å^3 kcal mol^{-1}	$4\pi N_F N_{C_H}/V$ in Å^{-3}	$U^{\text{eff}}_{FC_H}$ in kcal mol^{-1}
0.015	154422	-1.14	427.23	-486.29
0.023	157901.81	-1.14	417.81	-475.99
0.03	151589.95	-1.09	435.21	-476.5
0.038	146529.88	-1.04	450.24	-467.84
0.045	143301.98	-0.88	460.38	-403.21
0.053	140689.14	-0.65	468.93	-305.57
0.06	138991.83	-0.58	474.66	-276.51
$\varepsilon(H)$ in kcal mol^{-1}	V in Å^3	$\int r^2 g_{HC_F}(r) U_{HC_F}(r)\ dr$ in Å^3 kcal mol^{-1}	$4\pi N_H N_{C_F}/V$ in Å^{-3}	$U^{\text{eff}}_{HC_F}$ in kcal mol^{-1}
0.015	154422	-0.39	427.23	-165.56
0.023	157901.81	-0.49	417.81	-203.17
0.03	151589.95	-0.53	435.21	-229.52
0.038	146529.88	-0.57	450.24	-255.7
0.045	143301.98	-0.52	460.38	-237.6
0.053	140689.14	-0.42	468.93	-196.43
0.06	138991.83	-0.4	474.66	-189.43

continued on next page

Table S5: Effective interaction strengths as a function of $\varepsilon(H)$ - Extension.

parameter	box volume	integral	prefactor	U^{eff}
$\varepsilon(H)$ in kcal mol^{-1}	V in Å^3	$\int r^2 g_{C_H C_F}(r) U_{C_H C_F}(r)\ dr$ in Å^3 kcal mol^{-1}	$4\pi N_{C_H} N_{C_F}/V$ in Å^{-3}	$U^{\mathrm{eff}}_{C_H C_F}$ in kcal mol^{-1}
0.015	154422	-1.62	183.1	-297.13
0.023	157901.81	-1.62	179.06	-289.22
0.03	151589.95	-1.55	186.52	-289.13
0.038	146529.88	-1.47	192.96	-283.28
0.045	143301.98	-1.24	197.31	-244.05
0.053	140689.14	-0.92	200.97	-185.17
0.06	138991.83	-0.82	203.42	-167.29

Table S6: Effective interaction strengths of all pairwise atom-atom interactions in an equimolar hexane-perfluorohexane mixture as a function of the Lennard-Jones energy parameter of hexane carbon atoms $\varepsilon(C_H)$. Calculation according to Equation 1. The atom numbers used are $N_H = N_F = 3500$ and $N_{C_H} = N_{C_F} = 1500$.

parameter	box volume	integral	prefactor	U^{eff}
$\varepsilon(C_H)$ in kcal mol^-1	V in Å^3	$\int r^2 g_{HH}(r) U_{HH}(r)\ dr$ in Å^3 kcal mol^-1	$4\pi N_H N_H/2V$ in Å^-3	U^{eff}_{HH} in kcal mol^-1
0.012	202055.33	-0.24	380.93	-92.77
0.026	147532.75	-0.26	521.71	-136.27
0.039	138830.9	-0.27	554.41	-148.25
0.053	132885.23	-0.28	579.21	-164.83
0.066	129247.22	-0.32	595.52	-193.49
0.07	128252.81	-0.34	600.14	-201.2
0.073	127567.38	-0.34	603.36	-203.9
0.08	126354.87	-0.36	609.15	-218.05
0.093	124102.16	-0.38	620.21	-236.49
$\varepsilon(C_H)$ in kcal mol^-1	V in Å^3	$\int r^2 g_{HC_H}(r) U_{HC_H}(r)\ dr$ in Å^3 kcal mol^-1	$4\pi N_H N_{C_H}/2V$ in Å^-3	$U^{\text{eff}}_{HC_H}$ in kcal mol^-1
0.012	202055.33	-0.37	163.26	-59.75
0.026	147532.75	-0.61	223.59	-137.28
0.039	138830.9	-0.79	237.6	-188.1
0.053	132885.23	-1.03	248.23	-256.78
0.066	129247.22	-1.35	255.22	-343.51
0.07	128252.81	-1.47	257.2	-378.12
0.073	127567.38	-1.53	258.58	-394.52
0.08	126354.87	-1.71	261.06	-447.65
0.093	124102.16	-2.04	265.8	-542.27
$\varepsilon(C_H)$ in kcal mol^-1	V in Å^3	$\int r^2 g_{C_HC_H}(r) U_{C_HC_H}(r)\ dr$ in Å^3 kcal mol^-1	$4\pi N_{C_H} N_{C_H}/2V$ in Å^-3	$U^{\text{eff}}_{C_HC_H}$ in kcal mol^-1
0.012	202055.33	-0.46	69.97	-32.08
0.026	147532.75	-1.12	95.82	-107.69
0.039	138830.9	-1.79	101.83	-181.85
0.053	132885.23	-2.7	106.39	-286.73
0.066	129247.22	-3.97	109.38	-434.71
0.07	128252.81	-4.41	110.23	-485.77
0.073	127567.38	-4.67	110.82	-517.18
0.08	126354.87	-5.52	111.88	-617.57
0.093	124102.16	-7.06	113.92	-804.74
$\varepsilon(C_H)$ in kcal mol^-1	V in Å^3	$\int r^2 g_{FF}(r) U_{FF}(r)\ dr$ in Å^3 kcal mol^-1	$4\pi N_F N_F/2V$ in Å^-3	U^{eff}_{FF} in kcal mol^-1
0.012	202055.33	-1.28	380.93	-486.1
0.026	147532.75	-0.81	521.71	-422.3
0.039	138830.9	-0.76	554.41	-423.11
0.053	132885.23	-0.77	579.21	-444.35
0.066	129247.22	-0.82	595.52	-486.41
0.07	128252.81	-0.83	600.14	-497.86
0.073	127567.38	-0.83	603.36	-501.88

continued on next page

Table S6: Effective interaction strengths as a function of $\varepsilon(C_H)$ - Extension.

parameter	box volume	integral	prefactor	U^{eff}
$\varepsilon(C_H)$ in kcal mol^{-1}	V in Å^3	$\int r^2 g_{FF}(r) U_{FF}(r)\ dr$ in Å^3 kcal mol^{-1}	$4\pi N_F N_F / 2V$ in Å^{-3}	U^{eff}_{FF} in kcal mol^{-1}
0.08	126354.87	-0.86	609.15	-521.06
0.093	124102.16	-0.88	620.21	-548.53
$\varepsilon(C_H)$ in kcal mol^{-1}	V in Å^3	$\int r^2 g_{FC_F}(r) U_{FC_F}(r)\ dr$ in Å^3 kcal mol^{-1}	$4\pi N_F N_{C_F} / 2V$ in Å^{-3}	$U^{\text{eff}}_{FC_F}$ in kcal mol^{-1}
0.012	202055.33	-2.47	163.26	-403.38
0.026	147532.75	-1.57	223.59	-350.15
0.039	138830.9	-1.48	237.6	-351.54
0.053	132885.23	-1.5	248.23	-371.57
0.066	129247.22	-1.6	255.22	-408.32
0.07	128252.81	-1.63	257.2	-418.37
0.073	127567.38	-1.63	258.58	-422.2
0.08	126354.87	-1.68	261.06	-438.75
0.093	124102.16	-1.74	265.8	-462.83
$\varepsilon(C_H)$ in kcal mol^{-1}	V in Å^3	$\int r^2 g_{C_FC_F}(r) U_{C_FC_F}(r)\ dr$ in Å^3 kcal mol^{-1}	$4\pi N_{C_F} N_{C_F} / 2V$ in Å^{-3}	$U^{\text{eff}}_{C_FC_F}$ in kcal mol^{-1}
0.012	202055.33	-3.32	69.97	-232.59
0.026	147532.75	-2.11	95.82	-202.04
0.039	138830.9	-1.99	101.83	-202.89
0.053	132885.23	-2.02	106.39	-214.42
0.066	129247.22	-2.15	109.38	-235.64
0.07	128252.81	-2.19	110.23	-241.47
0.073	127567.38	-2.2	110.82	-243.62
0.08	126354.87	-2.26	111.88	-253.18
0.093	124102.16	-2.34	113.92	-266.89
$\varepsilon(C_H)$ in kcal mol^{-1}	V in Å^3	$\int r^2 g_{HF}(r) U_{HF}(r)\ dr$ in Å^3 kcal mol^{-1}	$4\pi N_H N_F / V$ in Å^{-3}	U^{eff}_{HF} in kcal mol^{-1}
0.012	202055.33	-0.16	761.86	-118.67
0.026	147532.75	-0.23	1043.42	-239.11
0.039	138830.9	-0.24	1108.82	-266.8
0.053	132885.23	-0.23	1158.43	-264.56
0.066	129247.22	-0.19	1191.04	-224.06
0.07	128252.81	-0.18	1200.27	-213.75
0.073	127567.38	-0.17	1206.72	-210.16
0.08	126354.87	-0.16	1218.3	-189.12
0.093	124102.16	-0.13	1240.41	-159.71
$\varepsilon(C_H)$ in kcal mol^{-1}	V in Å^3	$\int r^2 g_{FC_H}(r) U_{FC_H}(r)\ dr$ in Å^3 kcal mol^{-1}	$4\pi N_F N_{C_H} / V$ in Å^{-3}	$U^{\text{eff}}_{FC_H}$ in kcal mol^{-1}
0.012	202055.33	-0.26	326.51	-84.79
0.026	147532.75	-0.6	447.18	-266.31
0.039	138830.9	-0.79	475.21	-373.52
0.053	132885.23	-0.9	496.47	-448.03
0.066	129247.22	-0.84	510.44	-430.35

continued on next page

Table S6: Effective interaction strengths as a function of $\varepsilon(C_H)$ - Extension.

parameter	box volume	integral	prefactor	U^{eff}
$\varepsilon(C_H)$ in kcal mol^{-1}	V in Å^3	$\int r^2 g_{FC_H}(r) U_{FC_H}(r)\ dr$ in Å^3 kcal mol^{-1}	$4\pi N_F N_{C_H}/V$ in Å^{-3}	$U^{\text{eff}}_{FC_H}$ in kcal mol^{-1}
0.07	128252.81	-0.83	514.4	-426.07
0.073	127567.38	-0.83	517.17	-428.21
0.08	126354.87	-0.78	522.13	-404.95
0.093	124102.16	-0.7	531.61	-371.31
$\varepsilon(C_H)$ in kcal mol^{-1}	V in Å^3	$\int r^2 g_{HC_F}(r) U_{HC_F}(r)\ dr$ in Å^3 kcal mol^{-1}	$4\pi N_H N_{C_F}/V$ in Å^{-3}	$U^{\text{eff}}_{HC_F}$ in kcal mol^{-1}
0.012	202055.33	-0.29	326.51	-94.07
0.026	147532.75	-0.45	447.18	-201.97
0.039	138830.9	-0.49	475.21	-234.52
0.053	132885.23	-0.48	496.47	-240.03
0.066	129247.22	-0.41	510.44	-207.77
0.07	128252.81	-0.39	514.4	-199.06
0.073	127567.38	-0.38	517.17	-197.17
0.08	126354.87	-0.34	522.13	-177.84
0.093	124102.16	-0.29	531.61	-151.84
$\varepsilon(C_H)$ in kcal mol^{-1}	V in Å^3	$\int r^2 g_{C_H C_F}(r) U_{C_H C_F}(r)\ dr$ in Å^3 kcal mol^{-1}	$4\pi N_{C_H} N_{C_F}/V$ in Å^{-3}	$U^{\text{eff}}_{C_H C_F}$ in kcal mol^{-1}
0.012	202055.33	-0.37	139.93	-51.11
0.026	147532.75	-0.83	191.65	-159.05
0.039	138830.9	-1.12	203.66	-228.71
0.053	132885.23	-1.27	212.77	-270.29
0.066	129247.22	-1.2	218.76	-261.81
0.07	128252.81	-1.17	220.46	-258.55
0.073	127567.38	-1.17	221.64	-259.64
0.08	126354.87	-1.11	223.77	-248.18
0.093	124102.16	-0.99	227.83	-226.52

Table S7: Effective interaction strengths of all pairwise atom-atom interactions in an equimolar hexane-perfluorohexane mixture as a function of the Lennard-Jones energy parameter of fluorine atoms $\varepsilon(F)$. Calculation according to Equation 1. The atom numbers used are $N_H = N_F = 3500$ and $N_{C_H} = N_{C_F} = 1500$.

parameter	box volume	integral	prefactor	U^{eff}
$\varepsilon(F)$ in kcal mol^{-1}	V in Å^3	$\int r^2 g_{HH}(r) U_{HH}(r)\ dr$ in Å^3 kcal mol^{-1}	$4\pi N_H N_H/2V$ in Å^{-3}	U^{eff}_{HH} in kcal mol^{-1}
0.027	172715.64	-0.33	445.64	-146.09
0.035	156242.45	-0.27	492.63	-132.1
0.042	149467.67	-0.25	514.95	-130.29
0.053	142727.68	-0.24	539.27	-129.2
0.058	140770.3	-0.25	546.77	-136.38
0.064	138589.73	-0.25	555.37	-136.82
0.07	136352.31	-0.25	564.49	-138.88
0.075	134453.8	-0.25	572.46	-143.87
0.081	133589.56	-0.26	576.16	-149.21
0.088	131872.23	-0.27	583.66	-154.76
0.1	129477.42	-0.3	594.46	-177.55
$\varepsilon(F)$ in kcal mol^{-1}	V in Å^3	$\int r^2 g_{HC_H}(r) U_{HC_H}(r)\ dr$ in Å^3 kcal mol^{-1}	$4\pi N_H N_{C_H}/2V$ in Å^{-3}	$U^{\text{eff}}_{HC_H}$ in kcal mol^{-1}
0.027	172715.64	-1.34	190.99	-256.03
0.035	156242.45	-1.1	211.13	-232.18
0.042	149467.67	-1.04	220.69	-229.11
0.053	142727.68	-0.99	231.12	-228.3
0.058	140770.3	-1.03	234.33	-241.6
0.064	138589.73	-1.02	238.02	-243.3
0.07	136352.31	-1.02	241.92	-247.3
0.075	134453.8	-1.05	245.34	-257.39
0.081	133589.56	-1.08	246.93	-266.64
0.088	131872.23	-1.11	250.14	-276.89
0.1	129477.42	-1.25	254.77	-318.54
$\varepsilon(F)$ in kcal mol^{-1}	V in Å^3	$\int r^2 g_{C_HC_H}(r) U_{C_HC_H}(r)\ dr$ in Å^3 kcal mol^{-1}	$4\pi N_{C_H} N_{C_H}/2V$ in Å^{-3}	$U^{\text{eff}}_{C_HC_H}$ in kcal mol^{-1}
0.027	172715.64	-3.96	81.85	-323.91
0.035	156242.45	-3.25	90.48	-293.81
0.042	149467.67	-3.07	94.58	-289.93
0.053	142727.68	-2.92	99.05	-288.92
0.058	140770.3	-3.05	100.43	-305.89
0.064	138589.73	-3.02	102.01	-307.95
0.07	136352.31	-3.02	103.68	-313.05
0.075	134453.8	-3.1	105.15	-325.79
0.081	133589.56	-3.19	105.83	-337.42
0.088	131872.23	-3.27	107.2	-350.45
0.1	129477.42	-3.69	109.19	-403.32
$\varepsilon(F)$ in kcal mol^{-1}	V in Å^3	$\int r^2 g_{FF}(r) U_{FF}(r)\ dr$ in Å^3 kcal mol^{-1}	$4\pi N_F N_F/2V$ in Å^{-3}	U^{eff}_{FF} in kcal mol^{-1}
0.027	172715.64	-0.26	445.64	-117.26

continued on next page

Table S7: Effective interaction strengths as a function of $\varepsilon(F)$ - Extension.

parameter	box volume	integral	prefactor	U^{eff}
$\varepsilon(F)$ in kcal mol^{-1}	V in Å^3	$\int r^2 g_{FF}(r) U_{FF}(r)\ dr$ in Å^3 kcal mol^{-1}	$4\pi N_F N_F / 2V$ in Å^{-3}	U^{eff}_{FF} in kcal mol^{-1}
0.035	156242.45	-0.38	492.63	-185.92
0.042	149467.67	-0.48	514.95	-249.53
0.053	142727.68	-0.67	539.27	-359.12
0.058	140770.3	-0.77	546.77	-422.72
0.064	138589.73	-0.88	555.37	-490.71
0.07	136352.31	-1	564.49	-562.73
0.075	134453.8	-1.11	572.46	-635.65
0.081	133589.56	-1.26	576.16	-723.35
0.088	131872.23	-1.42	583.66	-831.23
0.1	129477.42	-1.83	594.46	-1087.46
$\varepsilon(F)$ in kcal mol^{-1}	V in Å^3	$\int r^2 g_{FC_F}(r) U_{FC_F}(r)\ dr$ in Å^3 kcal mol^{-1}	$4\pi N_F N_{C_F} / 2V$ in Å^{-3}	$U^{\text{eff}}_{FC_F}$ in kcal mol^{-1}
0.027	172715.64	-0.85	190.99	-163.28
0.035	156242.45	-1	211.13	-210.87
0.042	149467.67	-1.13	220.69	-248.89
0.053	142727.68	-1.3	231.12	-299.65
0.058	140770.3	-1.42	234.33	-332.08
0.064	138589.73	-1.52	238.02	-360.69
0.07	136352.31	-1.61	241.92	-390.6
0.075	134453.8	-1.71	245.34	-419.16
0.081	133589.56	-1.84	246.93	-455.5
0.088	131872.23	-1.98	250.14	-496.39
0.1	129477.42	-2.35	254.77	-599.49
$\varepsilon(F)$ in kcal mol^{-1}	V in Å^3	$\int r^2 g_{C_FC_F}(r) U_{C_FC_F}(r)\ dr$ in Å^3 kcal mol^{-1}	$4\pi N_{C_F} N_{C_F} / 2V$ in Å^{-3}	$U^{\text{eff}}_{C_FC_F}$ in kcal mol^{-1}
0.027	172715.64	-1.63	81.85	-133.75
0.035	156242.45	-1.66	90.48	-150.4
0.042	149467.67	-1.69	94.58	-160.28
0.053	142727.68	-1.74	99.05	-172.8
0.058	140770.3	-1.81	100.43	-182.01
0.064	138589.73	-1.85	102.01	-188.4
0.07	136352.31	-1.88	103.68	-194.85
0.075	134453.8	-1.93	105.15	-202.9
0.081	133589.56	-2	105.83	-211.32
0.088	131872.23	-2.06	107.2	-221.11
0.1	129477.42	-2.29	109.19	-250.17
$\varepsilon(F)$ in kcal mol^{-1}	V in Å^3	$\int r^2 g_{HF}(r) U_{HF}(r)\ dr$ in Å^3 kcal mol^{-1}	$4\pi N_H N_F / V$ in Å^{-3}	U^{eff}_{HF} in kcal mol^{-1}
0.027	172715.64	-0.13	891.28	-112.57
0.035	156242.45	-0.17	985.25	-169.29
0.042	149467.67	-0.2	1029.91	-202.03
0.053	142727.68	-0.24	1078.54	-263.35
0.058	140770.3	-0.24	1093.54	-265.35

continued on next page

Table S7: Effective interaction strengths as a function of $\varepsilon(F)$ - Extension.

parameter	box volume	integral	prefactor	U^{eff}
$\varepsilon(F)$ in kcal mol^{-1}	V in Å^3	$\int r^2 g_{HF}(r) U_{HF}(r)\ dr$ in Å^3 kcal mol^{-1}	$4\pi N_H N_F / V$ in Å^{-3}	U^{eff}_{HF} in kcal mol^{-1}
0.064	138589.73	-0.26	1110.75	-293.36
0.07	136352.31	-0.28	1128.97	-320.29
0.075	134453.8	-0.28	1144.91	-325.58
0.081	133589.56	-0.28	1152.32	-325.69
0.088	131872.23	-0.29	1167.33	-339.8
0.1	129477.42	-0.26	1188.92	-303.43
$\varepsilon(F)$ in kcal mol^{-1}	V in Å^3	$\int r^2 g_{FC_H}(r) U_{FC_H}(r)\ dr$ in Å^3 kcal mol^{-1}	$4\pi N_F N_{C_H} / V$ in Å^{-3}	$U^{\text{eff}}_{FC_H}$ in kcal mol^{-1}
0.027	172715.64	-0.62	381.98	-234.98
0.035	156242.45	-0.81	422.25	-343.83
0.042	149467.67	-0.94	441.39	-415.47
0.053	142727.68	-1.1	462.23	-508.07
0.058	140770.3	-1.11	468.66	-518.48
0.064	138589.73	-1.17	476.03	-557.16
0.07	136352.31	-1.23	483.85	-595.04
0.075	134453.8	-1.23	490.68	-604.01
0.081	133589.56	-1.23	493.85	-609.59
0.088	131872.23	-1.25	500.28	-623.5
0.1	129477.42	-1.07	509.54	-543.49
$\varepsilon(F)$ in kcal mol^{-1}	V in Å^3	$\int r^2 g_{HC_F}(r) U_{HC_F}(r)\ dr$ in Å^3 kcal mol^{-1}	$4\pi N_H N_{C_F} / V$ in Å^{-3}	$U^{\text{eff}}_{HC_F}$ in kcal mol^{-1}
0.027	172715.64	-0.42	381.98	-159.95
0.035	156242.45	-0.48	422.25	-204.52
0.042	149467.67	-0.51	441.39	-223.81
0.053	142727.68	-0.53	462.23	-245.51
0.058	140770.3	-0.51	468.66	-238.16
0.064	138589.73	-0.51	476.03	-244.02
0.07	136352.31	-0.51	483.85	-248.91
0.075	134453.8	-0.5	490.68	-245.36
0.081	133589.56	-0.48	493.85	-237.28
0.088	131872.23	-0.47	500.28	-232.91
0.1	129477.42	-0.37	509.54	-189.91
$\varepsilon(F)$ in kcal mol^{-1}	V in Å^3	$\int r^2 g_{C_H C_F}(r) U_{C_H C_F}(r)\ dr$ in Å^3 kcal mol^{-1}	$4\pi N_{C_H} N_{C_F} / V$ in Å^{-3}	$U^{\text{eff}}_{C_H C_F}$ in kcal mol^{-1}
0.027	172715.64	-1.24	163.7	-202.64
0.035	156242.45	-1.43	180.96	-258.24
0.042	149467.67	-1.49	189.17	-282.05
0.053	142727.68	-1.56	198.1	-308.62
0.058	140770.3	-1.49	200.85	-299.41
0.064	138589.73	-1.5	204.01	-306.51
0.07	136352.31	-1.51	207.36	-312.41
0.075	134453.8	-1.46	210.29	-307.94
0.081	133589.56	-1.41	211.65	-297.86

continued on next page

Table S7: Effective interaction strengths as a function of $\varepsilon(F)$ - Extension.

parameter	box volume	integral	prefactor	U^{eff}
$\varepsilon(F)$<br>in kcal mol^{-1}	V<br>in Å^3	$\int r^2 g_{C_H C_F}(r) U_{C_H C_F}(r)\ dr$<br>in Å^3 kcal mol^{-1}	$4\pi N_{C_H} N_{C_F}/V$<br>in Å^{-3}	$U^{\text{eff}}_{C_H C_F}$<br>in kcal mol^{-1}
0.088	131872.23	-1.36	214.41	-292.32
0.1	129477.42	-1.09	218.37	-238.8

Table S8: Effective interaction strengths of all pairwise atom-atom interactions in an equimolar hexane-perfluorohexane mixture as a function of the Lennard-Jones energy parameter of perfluorohexane carbon atoms $\varepsilon(C_F)$. Calculation according to Equation 1. The atom numbers used are $N_H = N_F = 3500$ and $N_{C_H} = N_{C_F} = 1500$.

parameter	box volume	integral	prefactor	U^{eff}
$\varepsilon(C_F)$ in kcal mol^{-1}	V in Å^3	$\int r^2 g_{HH}(r) U_{HH}(r)\ dr$ in Å^3 kcal mol^{-1}	$4\pi N_H N_H/2V$ in Å^{-3}	U^{eff}_{HH} in kcal mol^{-1}
0.026	173274.48	-0.31	444.2	-139.27
0.039	156677.99	-0.27	491.26	-131.72
0.053	139556.07	-0.25	551.53	-139.08
0.066	142727.68	-0.24	539.27	-129.2
0.08	138348.85	-0.24	556.34	-133.13
0.093	135242.69	-0.24	569.12	-136.84
0.107	132183.37	-0.24	582.29	-142.19
0.12	129861.7	-0.25	592.7	-146.96
0.134	128024.06	-0.26	601.21	-154.73
0.147	126128.38	-0.26	610.24	-158.97
0.161	124251.5	-0.29	619.46	-180.94
0.174	122911.74	-0.31	626.21	-191.7
$\varepsilon(C_F)$ in kcal mol^{-1}	V in Å^3	$\int r^2 g_{HC_H}(r) U_{HC_H}(r)\ dr$ in Å^3 kcal mol^{-1}	$4\pi N_H N_{C_H}/2V$ in Å^{-3}	$U^{\mathrm{eff}}_{HC_H}$ in kcal mol^{-1}
0.026	173274.48	-1.29	190.37	-244.96
0.039	156677.99	-1.1	210.54	-231.7
0.053	139556.07	-1.04	236.37	-244.75
0.066	142727.68	-0.99	231.12	-228.3
0.08	138348.85	-0.99	238.43	-236.16
0.093	135242.69	-1	243.91	-243.69
0.107	132183.37	-1.02	249.55	-254.06
0.12	129861.7	-1.04	254.01	-264.03
0.134	128024.06	-1.08	257.66	-277.42
0.147	126128.38	-1.1	261.53	-286.59
0.161	124251.5	-1.22	265.48	-324.85
0.174	122911.74	-1.28	268.38	-344.03
$\varepsilon(C_F)$ in kcal mol^{-1}	V in Å^3	$\int r^2 g_{C_HC_H}(r) U_{C_HC_H}(r)\ dr$ in Å^3 kcal mol^{-1}	$4\pi N_{C_H} N_{C_H}/2V$ in Å^{-3}	$U^{\mathrm{eff}}_{C_HC_H}$ in kcal mol^{-1}
0.026	173274.48	-3.8	81.59	-310
0.039	156677.99	-3.25	90.23	-293.14
0.053	139556.07	-3.06	101.3	-309.73
0.066	142727.68	-2.92	99.05	-288.92
0.08	138348.85	-2.92	102.18	-298.88
0.093	135242.69	-2.95	104.53	-308.42
0.107	132183.37	-3.01	106.95	-321.55
0.12	129861.7	-3.07	108.86	-334.3
0.134	128024.06	-3.18	110.43	-351.21
0.147	126128.38	-3.24	112.09	-362.72
0.161	124251.5	-3.61	113.78	-411.2
0.174	122911.74	-3.79	115.02	-435.46

continued on next page

Table S8: Effective interaction strengths as a function of $\varepsilon(C_F)$ - Extension.

parameter	box volume	integral	prefactor	U^{eff}
$\varepsilon(C_F)$ in kcal mol-1	V in Å^3	$\int r^2 g_{FF}(r) U_{FF}(r)\ dr$ in Å^3 kcal mol-1	$4\pi N_F N_F / 2V$ in Å-3	U^{eff}_{FF} in kcal mol-1
0.026	173274.48	-0.66	444.2	-292.86
0.039	156677.99	-0.67	491.26	-326.95
0.053	139556.07	-0.67	551.53	-369.19
0.066	142727.68	-0.67	539.27	-359.12
0.08	138348.85	-0.67	556.34	-372.11
0.093	135242.69	-0.68	569.12	-385.33
0.107	132183.37	-0.68	582.29	-396.24
0.12	129861.7	-0.68	592.7	-405.59
0.134	128024.06	-0.7	601.21	-418.04
0.147	126128.38	-0.69	610.24	-424.01
0.161	124251.5	-0.74	619.46	-461.11
0.174	122911.74	-0.76	626.21	-476.82
$\varepsilon(C_F)$ in kcal mol-1	V in Å^3	$\int r^2 g_{FC_F}(r) U_{FC_F}(r)\ dr$ in Å^3 kcal mol-1	$4\pi N_F N_{C_F} / 2V$ in Å-3	$U^{\text{eff}}_{FC_F}$ in kcal mol-1
0.026	173274.48	-0.72	190.37	-137.05
0.039	156677.99	-0.92	210.54	-193.88
0.053	139556.07	-1.13	236.37	-267.81
0.066	142727.68	-1.3	231.12	-299.65
0.08	138348.85	-1.49	238.43	-354.37
0.093	135242.69	-1.67	243.91	-407.24
0.107	132183.37	-1.87	249.55	-465.58
0.12	129861.7	-2.06	254.01	-524.17
0.134	128024.06	-2.28	257.66	-587.52
0.147	126128.38	-2.47	261.53	-645.71
0.161	124251.5	-2.86	265.48	-760.02
0.174	122911.74	-3.16	268.38	-847.56
$\varepsilon(C_F)$ in kcal mol-1	V in Å^3	$\int r^2 g_{C_FC_F}(r) U_{C_FC_F}(r)\ dr$ in Å^3 kcal mol-1	$4\pi N_{C_F} N_{C_F} / 2V$ in Å-3	$U^{\text{eff}}_{C_FC_F}$ in kcal mol-1
0.026	173274.48	-0.61	81.59	-49.74
0.039	156677.99	-0.96	90.23	-86.64
0.053	139556.07	-1.36	101.3	-138.01
0.066	142727.68	-1.74	99.05	-172.8
0.08	138348.85	-2.2	102.18	-224.82
0.093	135242.69	-2.67	104.53	-279.24
0.107	132183.37	-3.21	106.95	-343.08
0.12	129861.7	-3.73	108.86	-406.49
0.134	128024.06	-4.39	110.43	-485.12
0.147	126128.38	-4.99	112.09	-559.05
0.161	124251.5	-6.07	113.78	-690.09
0.174	122911.74	-6.94	115.02	-798.05
$\varepsilon(C_F)$ in kcal mol-1	V in Å^3	$\int r^2 g_{HF}(r) U_{HF}(r)\ dr$ in Å^3 kcal mol-1	$4\pi N_H N_F / V$ in Å-3	U^{eff}_{HF} in kcal mol-1
0.026	173274.48	-0.22	888.41	-192.42

continued on next page

Table S8: Effective interaction strengths as a function of $\varepsilon(C_F)$ - Extension.

parameter	box volume	integral	prefactor	U^{eff}
$\varepsilon(C_F)$ in kcal mol^{-1}	V in Å^3	$\int r^2 g_{HF}(r) U_{HF}(r)\ dr$ in Å^3 kcal mol^{-1}	$4\pi N_H N_F / V$ in Å^{-3}	U^{eff}_{HF} in kcal mol^{-1}
0.039	156677.99	-0.23	982.51	-229.88
0.053	139556.07	-0.24	1103.06	-263.97
0.066	142727.68	-0.24	1078.54	-263.35
0.08	138348.85	-0.24	1112.68	-265.27
0.093	135242.69	-0.23	1138.24	-264.45
0.107	132183.37	-0.22	1164.58	-259.7
0.12	129861.7	-0.21	1185.4	-254.05
0.134	128024.06	-0.2	1202.41	-240.21
0.147	126128.38	-0.19	1220.49	-235.08
0.161	124251.5	-0.15	1238.92	-190.27
0.174	122911.74	-0.13	1252.43	-168.41
$\varepsilon(C_F)$ in kcal mol^{-1}	V in Å^3	$\int r^2 g_{FC_H}(r) U_{FC_H}(r)\ dr$ in Å^3 kcal mol^{-1}	$4\pi N_F N_{C_H} / V$ in Å^{-3}	$U^{\text{eff}}_{FC_H}$ in kcal mol^{-1}
0.026	173274.48	-0.9	380.75	-342.19
0.039	156677.99	-1	421.08	-421.32
0.053	139556.07	-1.05	472.74	-497.53
0.066	142727.68	-1.1	462.23	-508.07
0.08	138348.85	-1.1	476.86	-522.86
0.093	135242.69	-1.09	487.82	-531.74
0.107	132183.37	-1.07	499.11	-532.86
0.12	129861.7	-1.04	508.03	-530.47
0.134	128024.06	-0.99	515.32	-509.76
0.147	126128.38	-0.97	523.07	-505.65
0.161	124251.5	-0.78	530.97	-413.24
0.174	122911.74	-0.69	536.75	-368.34
$\varepsilon(C_F)$ in kcal mol^{-1}	V in Å^3	$\int r^2 g_{HC_F}(r) U_{HC_F}(r)\ dr$ in Å^3 kcal mol^{-1}	$4\pi N_H N_{C_F} / V$ in Å^{-3}	$U^{\text{eff}}_{HC_F}$ in kcal mol^{-1}
0.026	173274.48	-0.27	380.75	-103.3
0.039	156677.99	-0.37	421.08	-155.59
0.053	139556.07	-0.46	472.74	-217.35
0.066	142727.68	-0.53	462.23	-245.51
0.08	138348.85	-0.59	476.86	-282.46
0.093	135242.69	-0.64	487.82	-311.85
0.107	132183.37	-0.68	499.11	-337.29
0.12	129861.7	-0.7	508.03	-354.29
0.134	128024.06	-0.7	515.32	-358.36
0.147	126128.38	-0.71	523.07	-372.94
0.161	124251.5	-0.6	530.97	-318.99
0.174	122911.74	-0.55	536.75	-296.85
$\varepsilon(C_F)$ in kcal mol^{-1}	V in Å^3	$\int r^2 g_{C_HC_F}(r) U_{C_HC_F}(r)\ dr$ in Å^3 kcal mol^{-1}	$4\pi N_{C_H} N_{C_F} / V$ in Å^{-3}	$U^{\text{eff}}_{C_HC_F}$ in kcal mol^{-1}
0.026	173274.48	-0.78	163.18	-127.98
0.039	156677.99	-1.09	180.46	-196.6

continued on next page

Table S8: Effective interaction strengths as a function of $\varepsilon(C_F)$ - Extension.

parameter	box volume	integral	prefactor	U^{eff}
$\varepsilon(C_F)$ in kcal mol-1	V in Å^3	$\int r^2 g_{C_H C_F}(r) U_{C_H C_F}(r)\ dr$ in Å^3 kcal mol-1	$4\pi N_{C_H} N_{C_F}/V$ in Å-3	$U^{\text{eff}}_{C_H C_F}$ in kcal mol-1
0.053	139556.07	-1.33	202.6	-269.36
0.066	142727.68	-1.56	198.1	-308.62
0.08	138348.85	-1.72	204.37	-352.03
0.093	135242.69	-1.84	209.06	-383.64
0.107	132183.37	-1.94	213.9	-415.17
0.12	129861.7	-2.01	217.73	-438.67
0.134	128024.06	-2.02	220.85	-446.32
0.147	126128.38	-2.06	224.17	-462.35
0.161	124251.5	-1.75	227.56	-397.94
0.174	122911.74	-1.6	230.04	-369.08

1.3 Elementary functions of large perfluorohexane carbon size

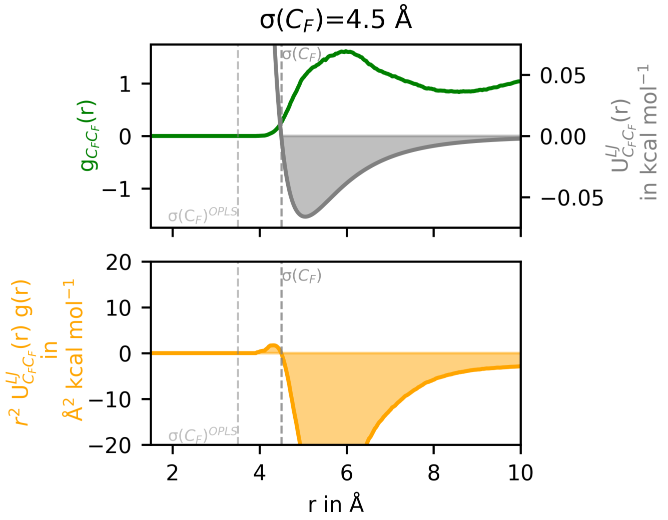

Figure S1: Elementary functions of the calculation of the effective interaction strength of the C_F–C_F interaction in an equimolar hexane-perfluorohexane mixture in a simulation with very large perfluorohexane carbon size parameter ($\sigma(C_F) = 4.5\,\text{Å}$). Upper panel: pairwise interaction potential $U^{LJ}_{C_F C_F}$ (grey) and pair correlation function $g_{C_F C_F}(r)$ (green). Lower panel: effective interaction potential $r^2 U^{LJ}_{C_F C_F} g_{C_F C_F}(r)$ (orange) as the integrand of Equation 1 in the manuscript.

1.4 Relative sensitivity of the effective interaction strengths

Table S9: Numerical values for the relative change in the effective interaction strength of the auto-interactions between hexane molecules when the parameters are changed around their OPLS values. The slopes $\frac{dU^{\text{eff}}_{\text{AB}}(\sigma)}{d\sigma}$ and $\frac{dU^{\text{eff}}_{\text{AB}}(\varepsilon)}{d\varepsilon}$ are calculated within finite limits around the OPLS values of σ or ε. Normalization with the respective OPLS values yielded $\sigma\frac{dU^{\text{eff}}_{\text{AB}}(\sigma)}{d\sigma}$ and $\varepsilon\frac{dU^{\text{eff}}_{\text{AB}}(\varepsilon)}{d\varepsilon}$.

parameter			H–H		H–C_H		C_H–C_H	
σ	σ^{OPLS} in Å	range in Å	$\frac{dU^{\text{eff}}_{HH}}{d\sigma}$ in $\frac{\text{kcal}}{\text{mol Å}}$	$\sigma\frac{dU^{\text{eff}}_{HH}}{d\sigma}$ in $\frac{\text{kcal}}{\text{mol}}$	$\frac{dU^{\text{eff}}_{HC_H}}{d\sigma}$ in $\frac{\text{kcal}}{\text{mol Å}}$	$\sigma\frac{dU^{\text{eff}}_{HC_H}}{d\sigma}$ in $\frac{\text{kcal}}{\text{mol}}$	$\frac{dU^{\text{eff}}_{C_HC_H}}{d\sigma}$ in $\frac{\text{kcal}}{\text{mol Å}}$	$\sigma\frac{dU^{\text{eff}}_{C_HC_H}}{d\sigma}$ in $\frac{\text{kcal}}{\text{mol}}$
$\sigma(H)$	2.5 [2]	2.5-3.25	-157	-392.5	-2	-5	230	575
$\sigma(C_H)$	3.5 [2]	3.0-3.88	-95	-332.5	-241	-843.5	-498	-1743
$\sigma(F)$	2.95 [3]	2.95-3.7	19	56	34	100.3	43	126.9
$\sigma(C_F)$	3.5 [3]	3.25-4	-5	-17.5	-12	-42	-15	-52.5
ε	$\varepsilon^{\text{OPLS}}$ in $\frac{\text{kcal}}{\text{mol}}$	range in $\frac{\text{kcal}}{\text{mol}}$	$\frac{dU^{\text{eff}}_{HH}}{d\varepsilon}$ in $\frac{\text{kcal/mol}}{\text{kcal/mol}}$	$\varepsilon\frac{dU^{\text{eff}}_{HH}}{d\varepsilon}$ in $\frac{\text{kcal}}{\text{mol}}$	$\frac{dU^{\text{eff}}_{HC_H}}{d\varepsilon}$ in $\frac{\text{kcal/mol}}{\text{kcal/mol}}$	$\varepsilon\frac{dU^{\text{eff}}_{HC_H}}{d\varepsilon}$ in $\frac{\text{kcal}}{\text{mol}}$	$\frac{dU^{\text{eff}}_{C_HC_H}}{d\varepsilon}$ in $\frac{\text{kcal/mol}}{\text{kcal/mol}}$	$\varepsilon\frac{dU^{\text{eff}}_{C_HC_H}}{d\varepsilon}$ in $\frac{\text{kcal}}{\text{mol}}$
$\varepsilon(H)$	0.03 [2]	0.015-0.045	-7736	-232.1	-7748	-232.4	-4662	-139.9
$\varepsilon(C_H)$	0.066 [2]	0.039-0.08	-1702	-112.3	-6330	-417.8	-10627	-701.4
$\varepsilon(F)$	0.053 [3]	0.042-0.081	-486	-25.8	-562	-29.8	-1218	-64.5
$\varepsilon(C_F$	0.066 [3]	0.039-0.08	-34	-2.2	-109	-7.2	-140	-9.2

Table S10: Numerical values for the relative change in the effective interaction strength of the auto-interactions between perfluorohexane molecules when the parameters are changed around their OPLS values. The slopes $\frac{dU_{\mathrm{AB}}^{\mathrm{eff}}(\sigma)}{d\sigma}$ and $\frac{dU_{\mathrm{AB}}^{\mathrm{eff}}(\varepsilon)}{d\varepsilon}$ are calculated within finite limits around the OPLS values of σ or ε. Normalization with the respective OPLS values yielded $\sigma\frac{dU_{\mathrm{AB}}^{\mathrm{eff}}(\sigma)}{d\sigma}$ and $\varepsilon\frac{dU_{\mathrm{AB}}^{\mathrm{eff}}(\varepsilon)}{d\varepsilon}$.

parameter			F–F		F–C_F		C_F–C_F	
σ	σ^{OPLS} in Å	range in Å	$\frac{dU_{FF}^{\mathrm{eff}}}{d\sigma}$ in $\frac{\mathrm{kcal}}{\mathrm{mol\ Å}}$	$\sigma\frac{dU_{FF}^{\mathrm{eff}}}{d\sigma}$ in $\frac{\mathrm{kcal}}{\mathrm{mol}}$	$\frac{dU_{FC_F}^{\mathrm{eff}}}{d\sigma}$ in $\frac{\mathrm{kcal}}{\mathrm{mol\ Å}}$	$\sigma\frac{dU_{FC_F}^{\mathrm{eff}}}{d\sigma}$ in $\frac{\mathrm{kcal}}{\mathrm{mol}}$	$\frac{dU_{C_FC_F}^{\mathrm{eff}}}{d\sigma}$ in $\frac{\mathrm{kcal}}{\mathrm{mol\ Å}}$	$\sigma\frac{dU_{C_FC_F}^{\mathrm{eff}}}{d\sigma}$ in $\frac{\mathrm{kcal}}{\mathrm{mol}}$
$\sigma(H)$	2.5 [2]	2.5-3.25	58	145	51	127.5	30	75
$\sigma(C_H)$	3.5 [2]	3.0-3.88	-71	-248.5	-68	-238	-39	-136.5
$\sigma(F)$	2.95 [3]	2.95-3.7	-290	-855.5	87	256.6	152	448.4
$\sigma(C_F)$	3.5 [3]	3.25-4	-96	-336	-320	-1120	-403	-1410.5
ε	$\varepsilon^{\mathrm{OPLS}}$ in $\frac{\mathrm{kcal}}{\mathrm{mol}}$	range in $\frac{\mathrm{kcal}}{\mathrm{mol}}$	$\frac{dU_{FF}^{\mathrm{eff}}}{d\varepsilon}$ in $\frac{\mathrm{kcal/mol}}{\mathrm{kcal/mol}}$	$\varepsilon\frac{dU_{FF}^{\mathrm{eff}}}{d\varepsilon}$ in $\frac{\mathrm{kcal}}{\mathrm{mol}}$	$\frac{dU_{FC_F}^{\mathrm{eff}}}{d\varepsilon}$ in $\frac{\mathrm{kcal/mol}}{\mathrm{kcal/mol}}$	$\varepsilon\frac{dU_{FC_F}^{\mathrm{eff}}}{d\varepsilon}$ in $\frac{\mathrm{kcal}}{\mathrm{mol}}$	$\frac{dU_{C_FC_F}^{\mathrm{eff}}}{d\varepsilon}$ in $\frac{\mathrm{kcal/mol}}{\mathrm{kcal/mol}}$	$\varepsilon\frac{dU_{C_FC_F}^{\mathrm{eff}}}{d\varepsilon}$ in $\frac{\mathrm{kcal}}{\mathrm{mol}}$
$\varepsilon(H)$	0.03 [2]	0.015-0.045	-2160	-64.8	-2018	-60.5	-1155	-34.6
$\varepsilon(C_H)$	0.066 [2]	0.039-0.08	-2389	-157.7	-2127	-140.4	-1227	-80.9
$\varepsilon(F)$	0.053 [3]	0.042-0.081	-12149	-643.9	-5298	-280.8	-1309	-69.4
$\varepsilon(C_F$	0.066 [3]	0.039-0.08	-101	-6.7	-3914	-258.3	-3370	-222.4

Table S11: Numerical values for the relative change in the effective interaction strength of the cross-interactions between hexane and perfluorohexane molecules when the parameters are changed around their OPLS values. The slopes $\frac{dU_{\mathrm{AB}}^{\mathrm{eff}}(\sigma)}{d\sigma}$ and $\frac{dU_{\mathrm{AB}}^{\mathrm{eff}}(\varepsilon)}{d\varepsilon}$ are calculated within finite limits around the OPLS values of σ or ε. Normalization with the respective OPLS values yielded $\sigma\frac{dU_{\mathrm{AB}}^{\mathrm{eff}}(\sigma)}{d\sigma}$ and $\varepsilon\frac{dU_{\mathrm{AB}}^{\mathrm{eff}}(\varepsilon)}{d\varepsilon}$.

parameter			H–F		F–C_H		H–C_F		C_H–C_F	
σ	σ^{OPLS} in Å	range in Å	$\frac{dU_{FH}^{\mathrm{eff}}}{d\sigma}$ in $\frac{\mathrm{kcal}}{\mathrm{mol\ Å}}$	$\sigma\frac{dU_{FH}^{\mathrm{eff}}}{d\sigma}$ in $\frac{\mathrm{kcal}}{\mathrm{mol}}$	$\frac{dU_{FC_H}^{\mathrm{eff}}}{d\sigma}$ in $\frac{\mathrm{kcal}}{\mathrm{mol\ Å}}$	$\sigma\frac{dU_{FC_H}^{\mathrm{eff}}}{d\sigma}$ in $\frac{\mathrm{kcal}}{\mathrm{mol}}$	$\frac{dU_{HC_F}^{\mathrm{eff}}}{d\sigma}$ in $\frac{\mathrm{kcal}}{\mathrm{mol\ Å}}$	$\sigma\frac{dU_{HC_F}^{\mathrm{eff}}}{d\sigma}$ in $\frac{\mathrm{kcal}}{\mathrm{mol}}$	$\frac{dU_{C_HC_F}^{\mathrm{eff}}}{d\sigma}$	$\sigma\frac{dU_{C_HC_F}^{\mathrm{eff}}}{d\sigma}$
$\sigma(H)$	2.5 [2]	2.5-3.25	-82	-205	210	525	-76	-190	141	-352.5
$\sigma(C_H)$	3.5 [2]	3.0-3.88	-14	-49	-302	-1057	2	7	-209	-731.5
$\sigma(F)$	2.95 [3]	2.95-3.7	-52	-153.4	-30	-88.5	132	389.4	159	469
$\sigma(C_F)$	3.5 [3]	3.25-4	-57	-199.5	-151	-528.5	-266	-931	-344	-1204
ε	$\varepsilon^{\mathrm{OPLS}}$ in $\frac{\mathrm{kcal}}{\mathrm{mol}}$	range in $\frac{\mathrm{kcal}}{\mathrm{mol}}$	$\frac{dU_{HF}^{\mathrm{eff}}}{d\varepsilon}$ in $\frac{\mathrm{kcal/mol}}{\mathrm{kcal/mol}}$	$\varepsilon\frac{dU_{HF}^{\mathrm{eff}}}{d\varepsilon}$ in $\frac{\mathrm{kcal}}{\mathrm{mol}}$	$\frac{dU_{FC_H}^{\mathrm{eff}}}{d\varepsilon}$ in $\frac{\mathrm{kcal/mol}}{\mathrm{kcal/mol}}$	$\varepsilon\frac{dU_{FC_H}^{\mathrm{eff}}}{d\varepsilon}$ in $\frac{\mathrm{kcal}}{\mathrm{mol}}$	$\frac{dU_{HC_F}^{\mathrm{eff}}}{d\varepsilon}$ in $\frac{\mathrm{kcal/mol}}{\mathrm{kcal/mol}}$	$\varepsilon\frac{dU_{HC_F}^{\mathrm{eff}}}{d\varepsilon}$ in $\frac{\mathrm{kcal}}{\mathrm{mol}}$	$\frac{dU_{C_HC_F}^{\mathrm{eff}}}{d\varepsilon}$	$\varepsilon\frac{dU_{C_HC_F}^{\mathrm{eff}}}{d\varepsilon}$
$\varepsilon(H)$	0.03 [2]	0.015-0.045	-3563	-106.9	2769	83.1	-2401	-72	1769	53.1
$\varepsilon(C_H)$	0.066 [2]	0.039-0.08	1895	125.1	-67	-4.4	1382	91.2	-475	-31.3
$\varepsilon(F)$	0.053 [3]	0.042-0.081	-3171	-168.1	-4977	-263.8	-345	-18.3	-405	-21.5
$\varepsilon(C_F$	0.066 [3]	0.039-0.08	-863	-57	-2477	-163.5	-3084	-203.5	-3791	-250.2

1.5 Calculation of total effective interaction energies for the size parameters

Grouped by their molecule type (hexane or perfluorohexane), the sums of the individual effective interaction strengths yield the total effective interaction energies for the auto- and cross-interactions (Equation 3 in the manuscript):

$$\begin{aligned} U^{\text{eff}}_{\text{auto,H}} &= U^{\text{eff}}_{H-H} + U^{\text{eff}}_{H-C_H} + U^{\text{eff}}_{C_H-C_H} \\ U^{\text{eff}}_{\text{auto,F}} &= U^{\text{eff}}_{F-F} + U^{\text{eff}}_{F-C_F} + U^{\text{eff}}_{C_F-C_F} \\ U^{\text{eff}}_{\text{cross,HF}} &= U^{\text{eff}}_{H-F} + U^{\text{eff}}_{H-C_F} + U^{\text{eff}}_{F-C_H} + U^{\text{eff}}_{C_H-C_F} \end{aligned} \tag{2}$$

Table S12: Total effective interaction energies of auto-interactions between hexane molecules as a function of the Lennard-Jones size parameters $\sigma(H)$, $\sigma(C_H)$, $\sigma(F)$, $\sigma(C_F)$. Calculation with Equation 2 from the individual effective interaction strengths in an equimolar hexane-perfluorohexane mixture.

parameter	value in Å	U^{eff}_{HH} in kcal mol-1	$U^{\text{eff}}_{HC_H}$ in kcal mol-1	$U^{\text{eff}}_{C_HC_H}$ in kcal mol-1	$U^{\text{eff}}_{\text{auto,H}}$ in kcal mol-1
$\sigma(H)$	1.87	-68.67	-164.05	-345.2	-578
$\sigma(H)$	2	-85.57	-188.36	-358.61	-633
$\sigma(H)$	2.12	-105.48	-219.56	-382.13	-707
$\sigma(H)$	2.25	-120.44	-236.99	-372.12	-730
$\sigma(H)$	2.37	-136.81	-256.46	-364.21	-757
$\sigma(H)$	2.5	-159.08	-281.48	-356.02	-797
$\sigma(H)$	2.62	-171.66	-285.22	-324.68	-782
$\sigma(H)$	2.75	-188.17	-288.41	-291.94	-769
$\sigma(H)$	2.87	-210.33	-295.76	-268.46	-775
$\sigma(H)$	3	-235.84	-299.27	-241.8	-777
$\sigma(H)$	3.12	-252.99	-289.94	-210.58	-754
$\sigma(H)$	3.25	-276.51	-283.12	-183.28	-743
$\sigma(H)$	3.37	-296.86	-274.09	-160.33	-731
$\sigma(H)$	3.5	-325.64	-267.14	-140.2	-733
$\sigma(H)$	3.62	-342.49	-253.55	-120.91	-717
$\sigma(H)$	3.75	-348.6	-230.93	-99.39	-679
$\sigma(C_H)$	2.63	-83.47	-71.93	-38.66	-194
$\sigma(C_H)$	2.75	-95.65	-97.09	-60.01	-253
$\sigma(C_H)$	2.88	-103.13	-120.36	-86.03	-310
$\sigma(C_H)$	3	-108.63	-144.16	-117.09	-370
$\sigma(C_H)$	3.13	-116.85	-172.81	-160.2	-450
$\sigma(C_H)$	3.25	-125.73	-200.73	-208.19	-535
$\sigma(C_H)$	3.38	-136.98	-232.26	-268.7	-638
$\sigma(C_H)$	3.5	-159.08	-281.48	-356.02	-797
$\sigma(C_H)$	3.63	-169.52	-307.88	-424.9	-902
$\sigma(C_H)$	3.75	-189.68	-349.7	-514.56	-1054
$\sigma(C_H)$	3.88	-192.11	-356.02	-554.99	-1103
$\sigma(C_H)$	4	-187.36	-351.94	-571.25	-1111
$\sigma(C_H)$	4.13	-177.3	-341.1	-574.29	-1093
$\sigma(C_H)$	4.25	-162.99	-323.58	-561.36	-1048
$\sigma(C_H)$	4.38	-147.98	-308.35	-553.02	-1009

continued on next page

Table S12: Total effective interactions energies for hexane auto-interactions as a function of σ - Extension.

parameter	value in Å	U^{eff}_{HH} in kcal mol^{-1}	$U^{\text{eff}}_{HC_H}$ in kcal mol^{-1}	$U^{\text{eff}}_{C_HC_H}$ in kcal mol^{-1}	$U^{\text{eff}}_{\text{auto,H}}$ in kcal mol^{-1}
$\sigma(C_H)$	4.5	-135.61	-297.85	-553.02	-986
$\sigma(F)$	2.7	-161.84	-287.27	-363.58	-813
$\sigma(F)$	2.95	-159.08	-281.48	-356.02	-797
$\sigma(F)$	3.08	-153.47	-272.52	-345.12	-771
$\sigma(F)$	3.2	-142.05	-252.07	-318.8	-713
$\sigma(F)$	3.33	-143.01	-253.76	-320.91	-718
$\sigma(F)$	3.45	-134.32	-237.41	-300.11	-672
$\sigma(F)$	3.58	-133.97	-238.41	-301.53	-674
$\sigma(F)$	3.7	-145.11	-256.24	-323.83	-725
$\sigma(F)$	3.95	-160.42	-283.8	-358.65	-803
$\sigma(F)$	4.2	-151.72	-267.5	-338.03	-757
$\sigma(C_F)$	2.75	-154.03	-269.59	-341.03	-765
$\sigma(C_F)$	2.87	-149.53	-261.74	-331.14	-742
$\sigma(C_F)$	3	-136.47	-239.89	-303.64	-680
$\sigma(C_F)$	3.13	-136.21	-240.73	-304.59	-682
$\sigma(C_F)$	3.25	-131.56	-232.08	-293.83	-657
$\sigma(C_F)$	3.37	-129.54	-229.42	-290.63	-650
$\sigma(C_F)$	3.5	-129.2	-228.3	-288.92	-646
$\sigma(C_F)$	3.62	-131.03	-234.15	-296.23	-661
$\sigma(C_F)$	3.75	-135.57	-240.01	-303.71	-679
$\sigma(C_F)$	3.87	-134.35	-241.16	-305.34	-681
$\sigma(C_F)$	4	-135.21	-241.18	-305.25	-682
$\sigma(C_F)$	4.12	-137.91	-246.03	-311.43	-695
$\sigma(C_F)$	4.25	-137.46	-248.22	-314.17	-700
$\sigma(C_F)$	4.37	-136.15	-245.19	-310.31	-692
$\sigma(C_F)$	4.5	-131.12	-236.07	-298.83	-666
$\sigma(C_F)$	4.62	-125.61	-227.93	-288.5	-642

Table S13: Total effective interaction energies of auto-interactions between perfluorohexane molecules as a function of the Lennard-Jones size parameters $\sigma(H)$, $\sigma(C_H)$, $\sigma(F)$, $\sigma(C_F)$. Calculation with Equation 2 from the individual effective interaction strengths in an equimolar hexane-perfluorohexane mixture.

parameter	value in Å	U^{eff}_{FF} in kcal mol-1	$U^{\text{eff}}_{FC_F}$ in kcal mol-1	$U^{\text{eff}}_{C_FC_F}$ in kcal mol-1	$U^{\text{eff}}_{\text{auto,F}}$ in kcal mol-1
$\sigma(H)$	1.87	-405.46	-338.72	-195.54	-940
$\sigma(H)$	2	-409.07	-342.96	-198.01	-950
$\sigma(H)$	2.12	-418.36	-350.47	-202.26	-971
$\sigma(H)$	2.25	-413.5	-346.98	-200.25	-961
$\sigma(H)$	2.37	-414.52	-347.74	-200.62	-963
$\sigma(H)$	2.5	-419.28	-351.23	-202.58	-973
$\sigma(H)$	2.62	-407.96	-343.03	-197.84	-949
$\sigma(H)$	2.75	-398.94	-333.77	-192.5	-925
$\sigma(H)$	2.87	-396.31	-331.16	-190.92	-918
$\sigma(H)$	3	-393.38	-328.31	-189.26	-911
$\sigma(H)$	3.12	-381.18	-318.78	-183.79	-884
$\sigma(H)$	3.25	-375.26	-312.96	-180.41	-869
$\sigma(H)$	3.37	-368.8	-307.79	-177.43	-854
$\sigma(H)$	3.5	-373.54	-309.16	-178.07	-861
$\sigma(H)$	3.62	-365.54	-303.82	-175.06	-844
$\sigma(H)$	3.75	-348.34	-288.6	-166.22	-803
$\sigma(C_H)$	2.63	-382.04	-316.42	-182.48	-881
$\sigma(C_H)$	2.75	-365.84	-301.14	-173.7	-841
$\sigma(C_H)$	2.88	-361.62	-297.72	-171.75	-831
$\sigma(C_H)$	3	-366.85	-302.37	-174.49	-844
$\sigma(C_H)$	3.13	-374.61	-311.96	-180.14	-867
$\sigma(C_H)$	3.25	-385.62	-321.2	-185.37	-892
$\sigma(C_H)$	3.38	-394.62	-327.99	-189.2	-912
$\sigma(C_H)$	3.5	-419.28	-351.23	-202.58	-973
$\sigma(C_H)$	3.63	-424.31	-356.35	-205.53	-986
$\sigma(C_H)$	3.75	-439.93	-370.79	-213.88	-1025
$\sigma(C_H)$	3.88	-429.06	-361.89	-208.73	-1000
$\sigma(C_H)$	4	-415.27	-351.02	-202.51	-969
$\sigma(C_H)$	4.13	-396.38	-335.04	-193.32	-925
$\sigma(C_H)$	4.25	-372.67	-315.88	-182.37	-871
$\sigma(C_H)$	4.38	-351.19	-297.61	-171.9	-821
$\sigma(C_H)$	4.5	-335.26	-283.89	-164.09	-783
$\sigma(F)$	2.7	-336.5	-345.1	-250.83	-932
$\sigma(F)$	2.95	-419.28	-351.23	-202.58	-973
$\sigma(F)$	3.08	-445.99	-335.43	-172.53	-954
$\sigma(F)$	3.2	-468.43	-318.86	-147.7	-935
$\sigma(F)$	3.33	-511.47	-312.16	-129.73	-953
$\sigma(F)$	3.45	-531.88	-293.9	-110.76	-937
$\sigma(F)$	3.58	-569.51	-282.29	-95.98	-948
$\sigma(F)$	3.7	-637.08	-286.16	-88.73	-1012
$\sigma(F)$	3.95	-764.72	-282.05	-72.89	-1120

continued on next page

Table S13: Total effective interactions energies for perfluorohexane auto-interactions as a function of σ - Extension.

parameter	value in Å	U^{eff}_{FF} in kcal mol^{-1}	$U^{\text{eff}}_{FC_F}$ in kcal mol^{-1}	$U^{\text{eff}}_{C_FC_F}$ in kcal mol^{-1}	$U^{\text{eff}}_{\text{auto,F}}$ in kcal mol^{-1}
$\sigma(F)$	4.2	-688.19	-209.53	-45.58	-943
$\sigma(C_F)$	2.75	-250	-96.99	-26.39	-373
$\sigma(C_F)$	2.87	-286.53	-128.19	-39.73	-454
$\sigma(C_F)$	3	-304.23	-157.34	-55.96	-518
$\sigma(C_F)$	3.13	-325.27	-193.17	-78.4	-597
$\sigma(C_F)$	3.25	-337.16	-225.42	-103.03	-666
$\sigma(C_F)$	3.37	-348.31	-259.97	-133.11	-741
$\sigma(C_F)$	3.5	-359.12	-299.65	-172.8	-832
$\sigma(C_F)$	3.62	-373.74	-343.57	-219.82	-937
$\sigma(C_F)$	3.75	-388.94	-390.85	-278.74	-1059
$\sigma(C_F)$	3.87	-399	-430.23	-336.82	-1166
$\sigma(C_F)$	4	-409.47	-465.63	-405.55	-1281
$\sigma(C_F)$	4.12	-425.44	-513.3	-478.48	-1417
$\sigma(C_F)$	4.25	-437.13	-552.39	-555.92	-1545
$\sigma(C_F)$	4.37	-442.83	-577.05	-618.84	-1639
$\sigma(C_F)$	4.5	-441.45	-590.72	-671.45	-1704
$\sigma(C_F)$	4.62	-439.05	-602.23	-715.88	-1757

Table S14: Total effective interaction energies of cross-interactions between hexane and perfluorohexane molecules as a function of the Lennard-Jones size parameters $\sigma(H)$, $\sigma(C_H)$, $\sigma(F)$, $\sigma(C_F)$. Calculation with Equation 2 from the individual effective interaction strengths in an equimolar hexane-perfluorohexane mixture.

parameter	value in Å	U^{eff}_{HF} in kcal mol^{-1}	$U^{\text{eff}}_{FC_H}$ in kcal mol^{-1}	$U^{\text{eff}}_{HC_F}$ in kcal mol^{-1}	$U^{\text{eff}}_{C_HC_F}$ in kcal mol^{-1}	$U^{\text{eff}}_{\text{cross,HF}}$ in kcal mol^{-1}
$\sigma(H)$	1.87	-232.51	-629.76	-166.95	-436.06	-1465
$\sigma(H)$	2	-242.33	-615.65	-188.13	-413.45	-1460
$\sigma(H)$	2.12	-239.61	-574.35	-197.21	-374.99	-1386
$\sigma(H)$	2.25	-249.1	-559.21	-216.33	-354.91	-1380
$\sigma(H)$	2.37	-251.68	-527.92	-227.37	-327.65	-1335
$\sigma(H)$	2.5	-248.34	-479.85	-231.98	-291.71	-1252
$\sigma(H)$	2.62	-264.69	-467.95	-249.25	-279.85	-1262
$\sigma(H)$	2.75	-279.83	-448.18	-266.12	-264.49	-1259
$\sigma(H)$	2.87	-282.03	-408.11	-267.74	-238.62	-1197
$\sigma(H)$	3	-287.77	-372.57	-272.05	-216.28	-1149
$\sigma(H)$	3.12	-304.52	-355.59	-286.64	-205.69	-1152
$\sigma(H)$	3.25	-310.09	-322.24	-288.93	-185.87	-1107
$\sigma(H)$	3.37	-316.04	-294.8	-290.99	-169.98	-1072
$\sigma(H)$	3.5	-306.75	-254.94	-278.92	-147.02	-988
$\sigma(H)$	3.62	-312.53	-234.08	-280.96	-135.18	-963
$\sigma(H)$	3.75	-321.21	-213.37	-283.53	-123.49	-942
$\sigma(C_H)$	2.63	-161.54	-148.72	-140.76	-80.62	-532
$\sigma(C_H)$	2.75	-205.89	-217.43	-182.96	-118.77	-725
$\sigma(C_H)$	2.88	-234.89	-283.3	-211.84	-156.71	-887
$\sigma(C_H)$	3	-248.65	-307.45	-229.74	-190.29	-976
$\sigma(C_H)$	3.13	-258.7	-396.11	-242.95	-226.29	-1124
$\sigma(C_H)$	3.25	-265.08	-441.79	-249.38	-257.25	-1213
$\sigma(C_H)$	3.38	-267.96	-486.48	-252.2	-288.74	-1295
$\sigma(C_H)$	3.5	-248.34	-479.85	-231.98	-291.71	-1252
$\sigma(C_H)$	3.63	-255.13	-519.61	-233.87	-323.37	-1332
$\sigma(C_H)$	3.75	-238.73	-509.13	-215.8	-324.97	-1289
$\sigma(C_H)$	3.88	-260.99	-572.99	-228.2	-374.68	-1437
$\sigma(C_H)$	4	-285.11	-647.59	-244.51	-434.03	-1611
$\sigma(C_H)$	4.13	-312.96	-724.92	-259.17	-497.87	-1795
$\sigma(C_H)$	4.25	-348.11	-818.5	-278.2	-574.54	-2019
$\sigma(C_H)$	4.38	-376	-901.87	-291.63	-648.45	-2218
$\sigma(C_H)$	4.5	-394.6	-961.75	-295.99	-303.43	-1956
$\sigma(F)$	2.7	-230.61	-460.19	-274.49	-344.01	-1309
$\sigma(F)$	2.95	-248.34	-479.85	-231.98	-291.71	-1252
$\sigma(F)$	3.08	-238.41	-449.14	-194.73	-245.93	-1128
$\sigma(F)$	3.2	-260.78	-484.3	-190.18	-241	-1176
$\sigma(F)$	3.33	-264.34	-480.83	-169.9	-216.37	-1131
$\sigma(F)$	3.45	-277.29	-499.6	-160.78	-205.63	-1143
$\sigma(F)$	3.58	-276.04	-488.01	-141.62	-182.25	-1088
$\sigma(F)$	3.7	-287.5	-502.04	-132.91	-172.08	-1095
$\sigma(F)$	3.95	-315.77	-537.92	-118.47	-155.11	-1127

continued on next page

Table S14: Total effective interactions energies of cross-interactions as a function of σ - Extension.

parameter	value in Å	U^{eff}_{HF} in kcal mol^{-1}	$U^{\text{eff}}_{FC_H}$ in kcal mol^{-1}	$U^{\text{eff}}_{HC_F}$ in kcal mol^{-1}	$U^{\text{eff}}_{C_HC_F}$ in kcal mol^{-1}	$U^{\text{eff}}_{\text{cross,HF}}$ in kcal mol^{-1}
$\sigma(F)$	4.2	-253.23	-421.44	-77.75	-102.99	-855
$\sigma(C_F)$	2.75	-147.83	-260.85	-63.39	-80	-552
$\sigma(C_F)$	2.87	-171.19	-306.48	-84.28	-106.39	-668
$\sigma(C_F)$	3	-208.61	-377.73	-118.48	-149.01	-854
$\sigma(C_F)$	3.13	-219.87	-404.88	-143.08	-180.34	-948
$\sigma(C_F)$	3.25	-237.94	-445.23	-174.97	-220.66	-1079
$\sigma(C_F)$	3.37	-253.8	-481.85	-209.5	-263.99	-1209
$\sigma(C_F)$	3.5	-263.35	-508.07	-245.51	-308.62	-1326
$\sigma(C_F)$	3.62	-266.46	-518.48	-272.38	-344.2	-1402
$\sigma(C_F)$	3.75	-267.93	-526.69	-302.81	-383.76	-1481
$\sigma(C_F)$	3.87	-273.55	-541.02	-336.21	-427.17	-1578
$\sigma(C_F)$	4	-280.65	-558.73	-374.88	-478.49	-1693
$\sigma(C_F)$	4.12	-276.69	-552.84	-396.67	-507.5	-1734
$\sigma(C_F)$	4.25	-283.42	-562.24	-431.65	-554.57	-1832
$\sigma(C_F)$	4.37	-290.8	-572.66	-466.21	-601.11	-1931
$\sigma(C_F)$	4.5	-305.15	-598.93	-517.03	-668.93	-2090
$\sigma(C_F)$	4.62	-317.39	-617.49	-561.37	-728.42	-2225

1.6 Calculation of total effective interaction energies for the energy parameters

Table S15: Total effective interaction energies of auto-interactions between hexane molecules as a function of the Lennard-Jones energy parameters $\varepsilon(H)$, $\varepsilon(C_H)$, $\varepsilon(F)$, $\varepsilon(C_F)$. Calculation with Equation 2 from the individual effective interaction strengths in an equimolar hexane-perfluorohexane mixture.

parameter	value in kcal mol^{-1}	U_{HH}^{eff} in kcal mol^{-1}	$U_{HC_H}^{\text{eff}}$ in kcal mol^{-1}	$U_{C_HC_H}^{\text{eff}}$ in kcal mol^{-1}	$U_{\text{auto,H}}^{\text{eff}}$ in kcal mol^{-1}
$\varepsilon(H)$	0.015	-34.92	-118.64	-218.64	-372
$\varepsilon(H)$	0.023	-70.36	-157.79	-227.84	-456
$\varepsilon(H)$	0.03	-115.85	-205.13	-260.06	-581
$\varepsilon(H)$	0.038	-180.55	-268.4	-297.38	-746
$\varepsilon(H)$	0.045	-266.99	-351.09	-358.51	-977
$\varepsilon(H)$	0.053	-405.57	-477.45	-444.38	-1327
$\varepsilon(H)$	0.06	-509.4	-551.37	-479.19	-1540
$\varepsilon(C_H)$	0.012	-92.77	-59.75	-32.08	-185
$\varepsilon(C_H)$	0.026	-136.27	-137.28	-107.69	-381
$\varepsilon(C_H)$	0.039	-148.25	-188.1	-181.85	-518
$\varepsilon(C_H)$	0.053	-164.83	-256.78	-286.73	-708
$\varepsilon(C_H)$	0.066	-193.49	-343.51	-434.71	-972
$\varepsilon(C_H)$	0.07	-201.2	-378.12	-485.77	-1065
$\varepsilon(C_H)$	0.073	-203.9	-394.52	-517.18	-1116
$\varepsilon(C_H)$	0.08	-218.05	-447.65	-617.57	-1283
$\varepsilon(C_H)$	0.093	-236.49	-542.27	-804.74	-1583
$\varepsilon(F)$	0.027	-146.09	-256.03	-323.91	-726
$\varepsilon(F)$	0.035	-132.1	-232.18	-293.81	-658
$\varepsilon(F)$	0.042	-130.29	-229.11	-289.93	-649
$\varepsilon(F)$	0.053	-129.2	-228.3	-288.92	-646
$\varepsilon(F)$	0.058	-136.38	-241.6	-305.89	-684
$\varepsilon(F)$	0.064	-136.82	-243.3	-307.95	-688
$\varepsilon(F)$	0.07	-138.88	-247.3	-313.05	-699
$\varepsilon(F)$	0.075	-143.87	-257.39	-325.79	-727
$\varepsilon(F)$	0.081	-149.21	-266.64	-337.42	-753
$\varepsilon(F)$	0.088	-154.76	-276.89	-350.45	-782
$\varepsilon(F)$	0.1	-177.55	-318.54	-403.32	-899
$\varepsilon(C_F)$	0.026	-139.27	-244.96	-310	-694
$\varepsilon(C_F)$	0.039	-131.72	-231.7	-293.14	-657
$\varepsilon(C_F)$	0.053	-139.08	-244.75	-309.73	-694
$\varepsilon(C_F)$	0.066	-129.2	-228.3	-288.92	-646
$\varepsilon(C_F)$	0.08	-133.13	-236.16	-298.88	-668
$\varepsilon(C_F)$	0.093	-136.84	-243.69	-308.42	-689
$\varepsilon(C_F)$	0.107	-142.19	-254.06	-321.55	-718
$\varepsilon(C_F)$	0.12	-146.96	-264.03	-334.3	-745
$\varepsilon(C_F)$	0.134	-154.73	-277.42	-351.21	-783
$\varepsilon(C_F)$	0.147	-158.97	-286.59	-362.72	-808
$\varepsilon(C_F)$	0.161	-180.94	-324.85	-411.2	-917
$\varepsilon(C_F)$	0.174	-191.7	-344.03	-435.46	-971

Table S16: Total effective interaction energies of auto-interactions between perfluorohexane molecules as a function of the Lennard-Jones energy parameters $\varepsilon(H)$, $\varepsilon(C_H)$, $\varepsilon(F)$, $\varepsilon(C_F)$. Calculation with Equation 2 from the individual effective interaction strengths in an equimolar hexane-perfluorohexane mixture.

parameter	value in kcal mol^{-1}	U^{eff}_{FF} in kcal mol^{-1}	$U^{\text{eff}}_{FC_F}$ in kcal mol^{-1}	$U^{\text{eff}}_{C_FC_F}$ in kcal mol^{-1}	$U^{\text{eff}}_{\text{auto,F}}$ in kcal mol^{-1}
$\varepsilon(H)$	0.015	-311.01	-254.61	-146.92	-713
$\varepsilon(H)$	0.023	-303.22	-249.23	-143.78	-696
$\varepsilon(H)$	0.03	-322.19	-267.23	-154.1	-744
$\varepsilon(H)$	0.038	-342.76	-284.99	-164.24	-792
$\varepsilon(H)$	0.045	-375.81	-315.14	-181.56	-873
$\varepsilon(H)$	0.053	-416.45	-347.93	-200.39	-965
$\varepsilon(H)$	0.06	-431.01	-360.84	-207.79	-1000
$\varepsilon(C_H)$	0.012	-486.1	-403.38	-232.59	-1122
$\varepsilon(C_H)$	0.026	-422.3	-350.15	-202.04	-974
$\varepsilon(C_H)$	0.039	-423.11	-351.54	-202.89	-978
$\varepsilon(C_H)$	0.053	-444.35	-371.57	-214.42	-1030
$\varepsilon(C_H)$	0.066	-486.41	-408.32	-235.64	-1130
$\varepsilon(C_H)$	0.07	-497.86	-418.37	-241.47	-1158
$\varepsilon(C_H)$	0.073	-501.88	-422.2	-243.62	-1168
$\varepsilon(C_H)$	0.08	-521.06	-438.75	-253.18	-1213
$\varepsilon(C_H)$	0.093	-548.53	-462.83	-266.89	-1278
$\varepsilon(F)$	0.027	-117.26	-163.28	-133.75	-414
$\varepsilon(F)$	0.035	-185.92	-210.87	-150.4	-547
$\varepsilon(F)$	0.042	-249.53	-248.89	-160.28	-659
$\varepsilon(F)$	0.053	-359.12	-299.65	-172.8	-832
$\varepsilon(F)$	0.058	-422.72	-332.08	-182.01	-937
$\varepsilon(F)$	0.064	-490.71	-360.69	-188.4	-1040
$\varepsilon(F)$	0.07	-562.73	-390.6	-194.85	-1148
$\varepsilon(F)$	0.075	-635.65	-419.16	-202.9	-1258
$\varepsilon(F)$	0.081	-723.35	-455.5	-211.32	-1390
$\varepsilon(F)$	0.088	-831.23	-496.39	-221.11	-1549
$\varepsilon(F)$	0.1	-1087.46	-599.49	-250.17	-1937
$\varepsilon(C_F)$	0.026	-292.86	-137.05	-49.74	-480
$\varepsilon(C_F)$	0.039	-326.95	-193.88	-86.64	-607
$\varepsilon(C_F)$	0.053	-369.19	-267.81	-138.01	-775
$\varepsilon(C_F)$	0.066	-359.12	-299.65	-172.8	-832
$\varepsilon(C_F)$	0.08	-372.11	-354.37	-224.82	-951
$\varepsilon(C_F)$	0.093	-385.33	-407.24	-279.24	-1072
$\varepsilon(C_F)$	0.107	-396.24	-465.58	-343.08	-1205
$\varepsilon(C_F)$	0.12	-405.59	-524.17	-406.49	-1336
$\varepsilon(C_F)$	0.134	-418.04	-587.52	-485.12	-1491
$\varepsilon(C_F)$	0.147	-424.01	-645.71	-559.05	-1629
$\varepsilon(C_F)$	0.161	-461.11	-760.02	-690.09	-1911
$\varepsilon(C_F)$	0.174	-476.82	-847.56	-798.05	-2122

Table S17: Total effective interaction energies of cross-interactions between hexane and perfluorohexane molecules as a function of the Lennard-Jones energy parameters $\varepsilon(H)$, $\varepsilon(C_H)$, $\varepsilon(F)$, $\varepsilon(C_F)$. Calculation with Equation 2 from the individual effective interaction strengths in an equimolar hexane-perfluorohexane mixture.

parameter	value in kcal mol^{-1}	U^{eff}_{HF} in kcal mol^{-1}	$U^{\text{eff}}_{FC_H}$ in kcal mol^{-1}	$U^{\text{eff}}_{HC_F}$ in kcal mol^{-1}	$U^{\text{eff}}_{C_HC_F}$ in kcal mol^{-1}	$U^{\text{eff}}_{\text{cross,HF}}$ in kcal mol^{-1}
$\varepsilon(H)$	0.015	-161.42	-486.29	-165.56	-297.13	-26
$\varepsilon(H)$	0.023	-207.9	-475.99	-203.17	-289.22	-24
$\varepsilon(H)$	0.03	-246.8	-476.5	-229.52	-289.13	83
$\varepsilon(H)$	0.038	-282.39	-467.84	-255.7	-283.28	249
$\varepsilon(H)$	0.045	-268.3	-403.21	-237.6	-244.05	696
$\varepsilon(H)$	0.053	-224.27	-305.57	-196.43	-185.17	1381
$\varepsilon(H)$	0.06	-222.19	-276.51	-189.43	-167.29	1684
$\varepsilon(C_H)$	0.012	-118.67	-84.79	-94.07	-51.11	-349
$\varepsilon(C_H)$	0.026	-239.11	-266.31	-201.97	-159.05	-866
$\varepsilon(C_H)$	0.039	-266.8	-373.52	-234.52	-228.71	-1104
$\varepsilon(C_H)$	0.053	-264.56	-448.03	-240.03	-270.29	-1223
$\varepsilon(C_H)$	0.066	-224.06	-430.35	-207.77	-261.81	-1124
$\varepsilon(C_H)$	0.07	-213.75	-426.07	-199.06	-258.55	-1097
$\varepsilon(C_H)$	0.073	-210.16	-428.21	-197.17	-259.64	-1095
$\varepsilon(C_H)$	0.08	-189.12	-404.95	-177.84	-248.18	-1020
$\varepsilon(C_H)$	0.093	-159.71	-371.31	-151.84	-226.52	-909
$\varepsilon(F)$	0.027	-112.57	-234.98	-159.95	-202.64	-710
$\varepsilon(F)$	0.035	-169.29	-343.83	-204.52	-258.24	-976
$\varepsilon(F)$	0.042	-202.03	-415.47	-223.81	-282.05	-1123
$\varepsilon(F)$	0.053	-263.35	-508.07	-245.51	-308.62	-1326
$\varepsilon(F)$	0.058	-265.35	-518.48	-238.16	-299.41	-1321
$\varepsilon(F)$	0.064	-293.36	-557.16	-244.02	-306.51	-1401
$\varepsilon(F)$	0.07	-320.29	-595.04	-248.91	-312.41	-1477
$\varepsilon(F)$	0.075	-325.58	-604.01	-245.36	-307.94	-1483
$\varepsilon(F)$	0.081	-325.69	-609.59	-237.28	-297.86	-1470
$\varepsilon(F)$	0.088	-339.8	-623.5	-232.91	-292.32	-1489
$\varepsilon(F)$	0.1	-303.43	-543.49	-189.91	-238.8	-1276
$\varepsilon(C_F)$	0.026	-192.42	-342.19	-103.3	-127.98	-766
$\varepsilon(C_F)$	0.039	-229.88	-421.32	-155.59	-196.6	-1003
$\varepsilon(C_F)$	0.053	-263.97	-497.53	-217.35	-269.36	-1248
$\varepsilon(C_F)$	0.066	-263.35	-508.07	-245.51	-308.62	-1326
$\varepsilon(C_F)$	0.08	-265.27	-522.86	-282.46	-352.03	-1423
$\varepsilon(C_F)$	0.093	-264.45	-531.74	-311.85	-383.64	-1492
$\varepsilon(C_F)$	0.107	-259.7	-532.86	-337.29	-415.17	-1545
$\varepsilon(C_F)$	0.12	-254.05	-530.47	-354.29	-438.67	-1577
$\varepsilon(C_F)$	0.134	-240.21	-509.76	-358.36	-446.32	-1555
$\varepsilon(C_F)$	0.147	-235.08	-505.65	-372.94	-462.35	-1576
$\varepsilon(C_F)$	0.161	-190.27	-413.24	-318.99	-397.94	-1320
$\varepsilon(C_F)$	0.174	-168.41	-368.34	-296.85	-369.08	-1203

1.7 Calculation of effective energies of mixing

The effective energy of mixing, which has the character of a mixing enthalpy, can be calculated from the total effective interaction energies by substracting the total effective interaction energies of the auto-interactions from those of the cross-interactions (Equation 4 of the manuscript):

$$\Delta U_{mix}^{\text{eff}} = U_{\text{cross,HF}}^{\text{eff}} - (U_{\text{auto,H}}^{\text{eff}} + U_{\text{auto,F}}^{\text{eff}}) \quad (3)$$

Table S18: Effective energy of mixing in an equimolar hexane-perfluorohexane mixture as a function of the Lennard-Jones size parameters $\sigma(H)$, $\sigma(C_H)$, $\sigma(F)$, $\sigma(C_F)$. Calculation according to Equation 3 from the effective interaction energies of the auto- and cross-interactions.

parameter	value in Å	$U_{\text{auto,H}}^{\text{eff}}$ in kcal mol^{-1}	$U_{\text{auto,F}}^{\text{eff}}$, in kcal mol^{-1}	$U_{\text{cross,HF}}^{\text{eff}}$ in kcal mol^{-1}	$\Delta U_{\text{mix}}^{\text{eff}}$ in kcal mol^{-1}
$\sigma(H)$	1.87	-578	-940	-1465	52
$\sigma(H)$	2	-633	-950	-1460	123
$\sigma(H)$	2.12	-707	-971	-1386	292
$\sigma(H)$	2.25	-730	-961	-1380	311
$\sigma(H)$	2.37	-757	-963	-1335	386
$\sigma(H)$	2.5	-797	-973	-1252	518
$\sigma(H)$	2.62	-782	-949	-1262	469
$\sigma(H)$	2.75	-769	-925	-1259	435
$\sigma(H)$	2.87	-775	-918	-1197	496
$\sigma(H)$	3	-777	-911	-1149	539
$\sigma(H)$	3.12	-754	-884	-1152	485
$\sigma(H)$	3.25	-743	-869	-1107	504
$\sigma(H)$	3.37	-731	-854	-1072	513
$\sigma(H)$	3.5	-733	-861	-988	606
$\sigma(H)$	3.62	-717	-844	-963	599
$\sigma(H)$	3.75	-679	-803	-942	540
$\sigma(C_H)$	2.63	-194	-881	-532	543
$\sigma(C_H)$	2.75	-253	-841	-725	368
$\sigma(C_H)$	2.88	-310	-831	-887	254
$\sigma(C_H)$	3	-370	-844	-976	237
$\sigma(C_H)$	3.13	-450	-867	-1124	192
$\sigma(C_H)$	3.25	-535	-892	-1213	213
$\sigma(C_H)$	3.38	-638	-912	-1295	254
$\sigma(C_H)$	3.5	-797	-973	-1252	518
$\sigma(C_H)$	3.63	-902	-986	-1332	557
$\sigma(C_H)$	3.75	-1054	-1025	-1289	790
$\sigma(C_H)$	3.88	-1103	-1000	-1437	666
$\sigma(C_H)$	4	-1111	-969	-1611	468
$\sigma(C_H)$	4.13	-1093	-925	-1795	223
$\sigma(C_H)$	4.25	-1048	-871	-2019	-101
$\sigma(C_H)$	4.38	-1009	-821	-2218	-388
$\sigma(C_H)$	4.5	-986	-783	-1956	-186
$\sigma(F)$	2.7	-813	-932	-1309	436
$\sigma(F)$	2.95	-797	-973	-1252	518
$\sigma(F)$	3.08	-771	-954	-1128	597

continued on next page

Table S18: Effective energy of mixing as a function of σ - Extension.

parameter	value in Å	$U^{\mathrm{eff}}_{\mathrm{auto,H}}$ in kcal mol^{-1}	$U^{\mathrm{eff}}_{\mathrm{auto,F}}$, in kcal mol^{-1}	$U^{\mathrm{eff}}_{\mathrm{cross,HF}}$ in kcal mol^{-1}	$\Delta U^{\mathrm{eff}}_{\mathrm{mix}}$ in kcal mol^{-1}
$\sigma(F)$	3.2	-713	-935	-1176	472
$\sigma(F)$	3.33	-718	-953	-1131	540
$\sigma(F)$	3.45	-672	-937	-1143	465
$\sigma(F)$	3.58	-674	-948	-1088	534
$\sigma(F)$	3.7	-725	-1012	-1095	643
$\sigma(F)$	3.95	-803	-1120	-1127	795
$\sigma(F)$	4.2	-757	-943	-855	845
$\sigma(C_F)$	2.75	-765	-373	-552	586
$\sigma(C_F)$	2.87	-742	-454	-668	529
$\sigma(C_F)$	3	-680	-518	-854	344
$\sigma(C_F)$	3.13	-682	-597	-948	330
$\sigma(C_F)$	3.25	-657	-666	-1079	244
$\sigma(C_F)$	3.37	-650	-741	-1209	182
$\sigma(C_F)$	3.5	-646	-832	-1326	152
$\sigma(C_F)$	3.62	-661	-937	-1402	197
$\sigma(C_F)$	3.75	-679	-1059	-1481	257
$\sigma(C_F)$	3.87	-681	-1166	-1578	269
$\sigma(C_F)$	4	-682	-1281	-1693	270
$\sigma(C_F)$	4.12	-695	-1417	-1734	379
$\sigma(C_F)$	4.25	-700	-1545	-1832	413
$\sigma(C_F)$	4.37	-692	-1639	-1931	400
$\sigma(C_F)$	4.5	-666	-1704	-2090	280
$\sigma(C_F)$	4.62	-642	-1757	-2225	175

Table S19: Effective energy of mixing in an equimolar hexane-perfluorohexane mixture as a function of the Lennard-Jones energy parameters $\varepsilon(H)$, $\varepsilon(C_H)$, $\varepsilon(F)$, $\varepsilon(C_F)$. Calculation according to Equation 3 from the effective interaction energies of the auto- and cross-interactions.

parameter	value in kcal mol-1	$U^{\mathrm{eff}}_{\mathrm{auto,H}}$ in kcal mol-1	$U^{\mathrm{eff}}_{\mathrm{auto,F}}$, in kcal mol-1	$U^{\mathrm{eff}}_{\mathrm{cross,HF}}$ in kcal mol-1	$\Delta U^{\mathrm{eff}}_{\mathrm{mix}}$ in kcal mol-1
$\varepsilon(H)$	0.015	-372	-713	-1110	-26
$\varepsilon(H)$	0.023	-456	-696	-1176	-24
$\varepsilon(H)$	0.03	-581	-744	-1242	83
$\varepsilon(H)$	0.038	-746	-792	-1289	249
$\varepsilon(H)$	0.045	-977	-873	-1153	696
$\varepsilon(H)$	0.053	-1327	-965	-911	1381
$\varepsilon(H)$	0.06	-1540	-1000	-855	1684
$\varepsilon(C_H)$	0.012	-185	-1122	-349	958
$\varepsilon(C_H)$	0.026	-381	-974	-866	489
$\varepsilon(C_H)$	0.039	-518	-978	-1104	392
$\varepsilon(C_H)$	0.053	-708	-1030	-1223	516
$\varepsilon(C_H)$	0.066	-972	-1130	-1124	978
$\varepsilon(C_H)$	0.07	-1065	-1158	-1097	1125
$\varepsilon(C_H)$	0.073	-1116	-1168	-1095	1188
$\varepsilon(C_H)$	0.08	-1283	-1213	-1020	1476
$\varepsilon(C_H)$	0.093	-1583	-1278	-909	1952
$\varepsilon(F)$	0.027	-726	-414	-710	430
$\varepsilon(F)$	0.035	-658	-547	-976	229
$\varepsilon(F)$	0.042	-649	-659	-1123	185
$\varepsilon(F)$	0.053	-646	-832	-1326	152
$\varepsilon(F)$	0.058	-684	-937	-1321	299
$\varepsilon(F)$	0.064	-688	-1040	-1401	327
$\varepsilon(F)$	0.07	-699	-1148	-1477	371
$\varepsilon(F)$	0.075	-727	-1258	-1483	502
$\varepsilon(F)$	0.081	-753	-1390	-1470	673
$\varepsilon(F)$	0.088	-782	-1549	-1489	842
$\varepsilon(F)$	0.1	-899	-1937	-1276	1561
$\varepsilon(C_F)$	0.026	-694	-480	-766	408
$\varepsilon(C_F)$	0.039	-657	-607	-1003	261
$\varepsilon(C_F)$	0.053	-694	-775	-1248	220
$\varepsilon(C_F)$	0.066	-646	-832	-1326	152
$\varepsilon(C_F)$	0.08	-668	-951	-1423	197
$\varepsilon(C_F)$	0.093	-689	-1072	-1492	269
$\varepsilon(C_F)$	0.107	-718	-1205	-1545	378
$\varepsilon(C_F)$	0.12	-745	-1336	-1577	504
$\varepsilon(C_F)$	0.134	-783	-1491	-1555	719
$\varepsilon(C_F)$	0.147	-808	-1629	-1576	861
$\varepsilon(C_F)$	0.161	-917	-1911	-1320	1508
$\varepsilon(C_F)$	0.174	-971	-2122	-1203	1891

2 Additional results

2.1 Density as a function of the Lennard-Jones energy parameters

Figure S2 shows the global density as a function of the different Lennard-Jones energy parameters. The density of the system increases with the energy parameters of all atom types. This behavior complies with expectations. The deeper the interaction potential well, the more attractive are the intermolecular interactions, which leads to an overall compression of the system. At high values of ε, the increase is levelling off due to arising repulsive forces.

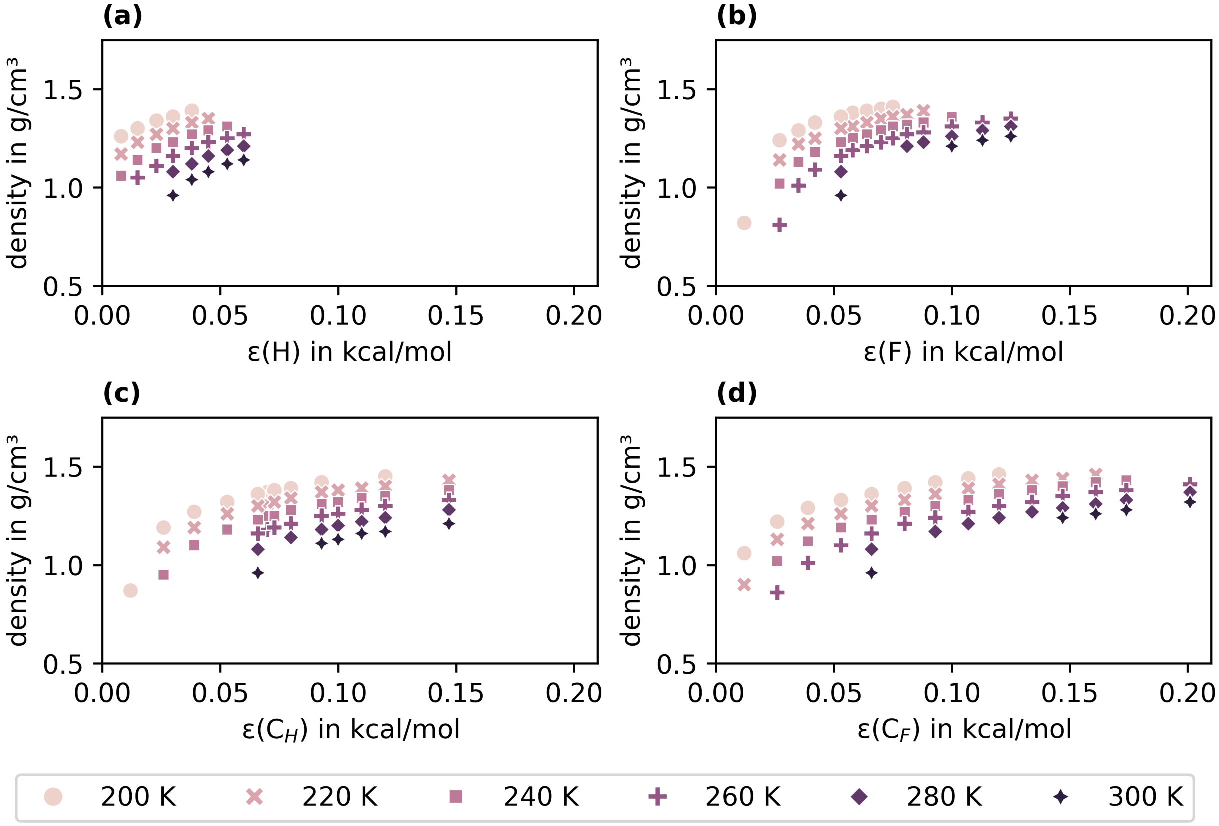

Figure S2: Global density of an equimolar hexane-perfluorohexane mixture as a function of the Lennard-Jones energy parameters: **a)** $\varepsilon(H)$, **b)** $\varepsilon(F)$, **c)** $\varepsilon(C_H)$, **d)** $\varepsilon(C_F)$. Associated simulation data were reported in reference [4].

2.2 Thermal expansion coefficients

For the whole simulation data set in reference [4], isobaric coefficients of thermal expansion were calculated as functions of the individual Lennard-Jones parameters from the change in box volume with temperature according to equation 4,

$$\alpha = \frac{1}{V_0}\left(\frac{\partial V}{\partial T}\right)_p \tag{4}$$

where V_0 equals the box volume at the lowest simulated temperature of each series. Results are summarized in Table S20 for variation in size parameters σ and in Table S21 for variation in energy parametes ε. Corresponding plots are shown in Figure S3 and Figure S4, respectively. In line with the density results, the cofficients of thermal expansion decrease with the energy parameters as intermolecular interactions are strengthened.

Table S20: Isobaric coefficents of thermal expansion determined from the linear change in box volume with temperature for different σ.

parameter	value in Å	range of T in K	V_0 in nm^3	$\frac{\partial V}{\partial T}$ in nm^3/K	α in 10^{-3}/K
$\sigma(H)$	2	200-260	119.31	0.3107	2.6
$\sigma(H)$	2.12	200-240	120.85	0.2912	2.41
$\sigma(H)$	2.25	200-240	123.13	0.3067	2.49
$\sigma(H)$	2.37	200-240	125.83	0.3231	2.57
$\sigma(H)$	2.5	200-280	129.25	0.417	3.23
$\sigma(H)$	2.62	200-260	132.73	0.4016	3.03
$\sigma(H)$	2.75	200-260	137.31	0.4387	3.19
$\sigma(H)$	2.87	200-260	141.66	0.4749	3.35
$\sigma(H)$	3	200-260	147.03	0.5499	3.74
$\sigma(H)$	3.12	200-260	153.04	0.5976	3.9
$\sigma(H)$	3.25	220-260	169.85	0.7871	4.63
$\sigma(H)$	3.37	220-260	177.6	0.9169	5.16
$\sigma(H)$	3.5	200-260	175.43	0.94	5.36
$\sigma(H)$	3.62	200-260	183.44	1.1723	6.39
$\sigma(H)$	3.75	200-260	194.2	1.5851	8.16
$\sigma(F)$	2.45	200-260	110.38	0.2598	2.35
$\sigma(F)$	2.7	200-240	118.8	0.2976	2.51
$\sigma(F)$	2.95	200-280	129.25	0.417	3.23
$\sigma(F)$	3.2	200-260	141.66	0.4491	3.17
$\sigma(F)$	3.45	200-260	156.85	0.5456	3.48
$\sigma(F)$	3.7	200-260	174.77	0.718	4.11
$\sigma(F)$	3.95	200-260	197.65	1.078	5.45
$\sigma(C_H)$	2.88	200-240	143.220	0.7841	5.47
$\sigma(C_H)$	3	200-240	138.590	0.6001	4.33
$\sigma(C_H)$	3.13	200-240	135.085	0.4856	3.59
$\sigma(C_H)$	3.25	200-240	132.573	0.4224	3.19
$\sigma(C_H)$	3.38	200-240	130.247	0.38	2.92
$\sigma(C_H)$	3.5	200-280	129.247	0.417	3.23
$\sigma(C_H)$	3.63	200-260	128.329	0.3413	2.66
$\sigma(C_H)$	3.75	200-260	128.558	0.3161	2.46
$\sigma(C_H)$	3.88	200-260	128.941	0.2946	2.28

continued on next page

Table S20: Isobaric coefficents of thermal expansion for different σ - Extension.

parameter	value in Å	range of T in K	V_0 in nm^3	$\frac{\partial V}{\partial T}$ in nm^3/K	α in $10^{-3}/K$
$\sigma(C_H)$	4	200-280	130.016	0.3066	2.36
$\sigma(C_H)$	4.13	200-280	131.484	0.2892	2.2
$\sigma(C_H)$	4.25	200-280	133.276	0.2808	2.11
$\sigma(C_H)$	4.38	200-260	135.401	0.2588	1.91
$\sigma(C_H)$	4.5	200-260	137.868	0.258	1.87
$\sigma(C_F)$	3	200-260	140.85	0.7987	5.67
$\sigma(C_F)$	3.13	200-260	136.91	0.6032	4.41
$\sigma(C_F)$	3.25	200-260	133.98	0.5093	3.8
$\sigma(C_F)$	3.37	200-260	131.64	0.4288	3.26
$\sigma(C_F)$	3.5	200-280	129.25	0.417	3.23
$\sigma(C_F)$	3.62	200-260	127.49	0.3324	2.61
$\sigma(C_F)$	3.75	200-260	125.68	0.3008	2.39
$\sigma(C_F)$	3.87	200-260	124.55	0.2742	2.2
$\sigma(C_F)$	4	200-280	123.80	0.2676	2.16
$\sigma(C_F)$	4.12	200-280	123.36	0.2462	2
$\sigma(C_F)$	4.25	200-280	123.36	0.2269	1.84
$\sigma(C_F)$	4.37	200-280	123.58	0.2166	1.75
$\sigma(C_F)$	4.5	200-280	124.33	0.2032	1.63
$\sigma(C_F)$	4.62	200-260	125.30	0.1859	1.48

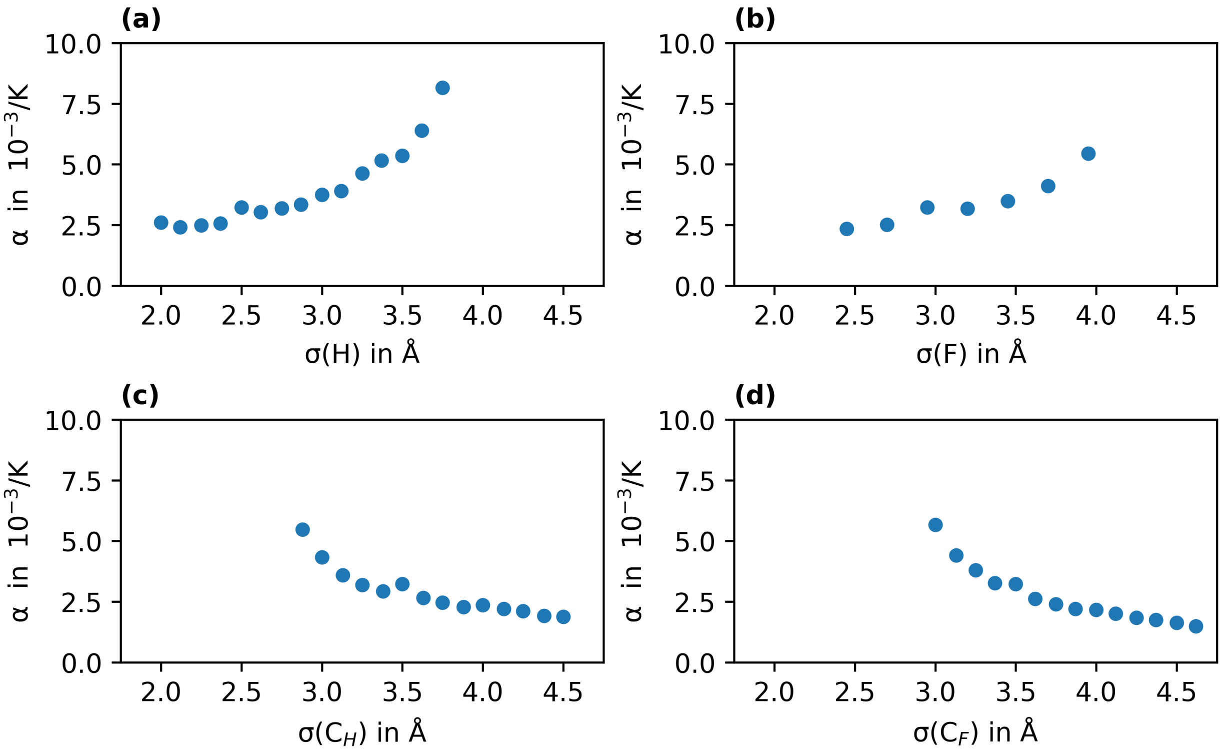

Figure S3: Isobaric coefficients of thermal expansion dependent on the Lennard-Jones size parameters.

Table S21: Isobaric coefficents of thermal expansion determined from the linear change in box volume with temperature for different ε.

parameter	value in kcal mol^{-1}	range of T in K	V_0 in nm^3	$\frac{\partial V}{\partial T}$ in nm^3/K	α in 10^{-3}/K
$\varepsilon(H)$	0.008	200-240	140.04	0.6470	4.62
$\varepsilon(H)$	0.015	200-260	135.64	0.5455	4.02
$\varepsilon(H)$	0.023	200-260	131.48	0.4368	3.32
$\varepsilon(H)$	0.030	200-280	129.25	0.4170	3.23
$\varepsilon(H)$	0.038	200-280	127.04	0.3649	2.87
$\varepsilon(H)$	0.045	220-280	130.40	0.3523	2.70
$\varepsilon(H)$	0.053	240-280	134.38	0.3457	2.57
$\varepsilon(F)$	0.027	200-240	142.32	0.7599	5.34
$\varepsilon(F)$	0.035	200-260	135.95	0.6301	4.63
$\varepsilon(F)$	0.042	200-260	132.50	0.4863	3.67
$\varepsilon(F)$	0.053	200-280	129.25	0.417	3.23
$\varepsilon(F)$	0.058	200-260	127.87	0.3402	2.66
$\varepsilon(F)$	0.064	200-260	126.58	0.3141	2.48
$\varepsilon(F)$	0.07	200-260	125.30	0.2993	2.39
$\varepsilon(F)$	0.075	200-260	124.78	0.2669	2.14
$\varepsilon(F)$	0.081	220-280	128.10	0.2902	2.27
$\varepsilon(F)$	0.088	220-280	127.04	0.2691	2.12
$\varepsilon(F)$	0.1	240-280	129.48	0.2520	1.95
$\varepsilon(C_H)$	0.026	200-240	147.53	0.9391	6.37
$\varepsilon(C_H)$	0.039	200-240	138.83	0.5362	3.86
$\varepsilon(C_H)$	0.053	200-240	132.89	0.4061	3.06
$\varepsilon(C_H)$	0.066	200-280	129.25	0.417	3.23
$\varepsilon(C_H)$	0.07	200-260	128.25	0.3564	2.78
$\varepsilon(C_H)$	0.073	200-260	127.57	0.3434	2.69
$\varepsilon(C_H)$	0.08	200-280	126.35	0.3507	2.78
$\varepsilon(C_H)$	0.093	200-280	124.10	0.3114	2.51
$\varepsilon(C_H)$	0.1	220-280	127.95	0.3123	2.44
$\varepsilon(C_H)$	0.11	220-300	126.51	0.3193	2.52
$\varepsilon(C_H)$	0.12	200-280	121.29	0.258	2.13
$\varepsilon(C_H)$	0.147	220-280	123.43	0.2389	1.94
$\varepsilon(C_F)$	0.026	200-240	144.79	0.9775	4.92
$\varepsilon(C_F)$	0.039	200-240	136.91	0.6037	3.61
$\varepsilon(C_F)$	0.053	200-260	132.34	0.4491	3.39
$\varepsilon(C_F)$	0.066	200-280	129.25	0.417	3.23
$\varepsilon(C_F)$	0.08	200-260	126.28	0.3291	2.61
$\varepsilon(C_F)$	0.093	200-280	124.25	0.3185	2.56
$\varepsilon(C_F)$	0.107	200-280	122.25	0.2885	2.36
$\varepsilon(C_F)$	0.12	200-280	120.70	0.264	2.19
$\varepsilon(C_F)$	0.134	220-280	123.06	0.2572	2.09
$\varepsilon(C_F)$	0.147	220-280	121.95	0.2342	1.92
$\varepsilon(C_F)$	0.161	220-280	120.26	0.2296	1.91
$\varepsilon(C_F)$	0.174	240-280	122.91	0.2279	1.85

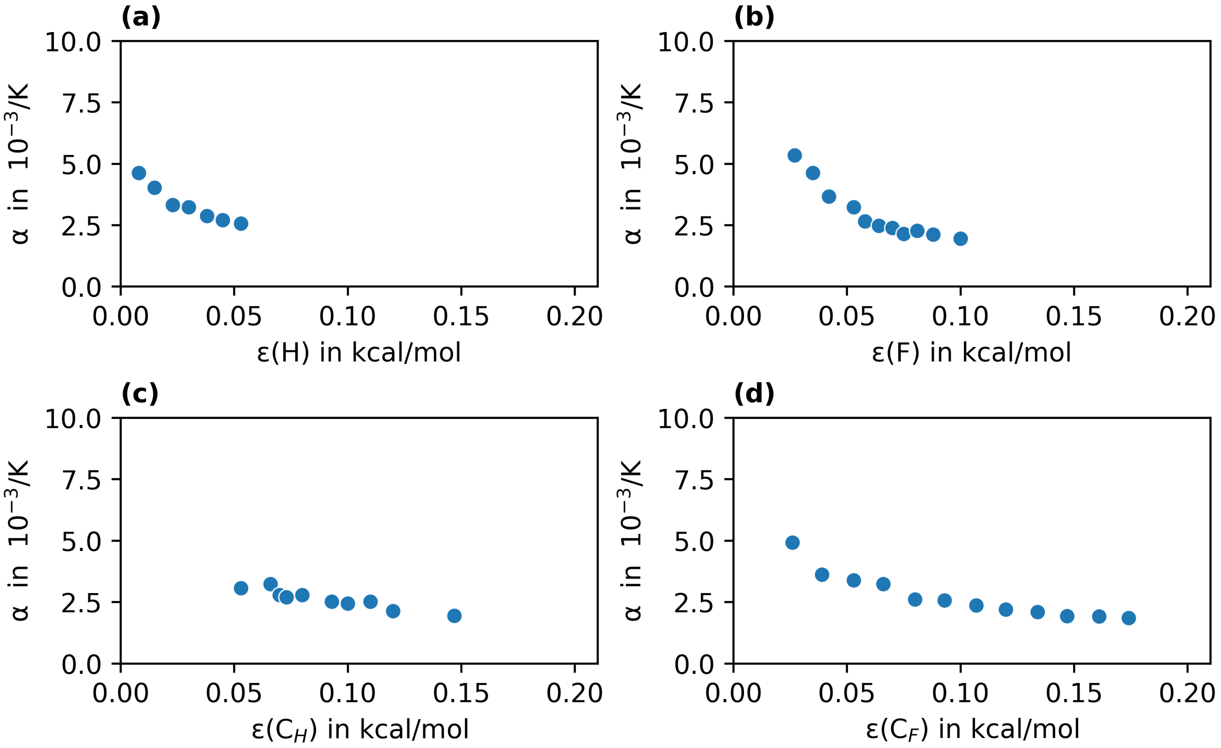

Figure S4: Isobaric coefficients of thermal expansion dependent on the Lennard-Jones energy parameters.

2.3 Energy of mixing diagrams

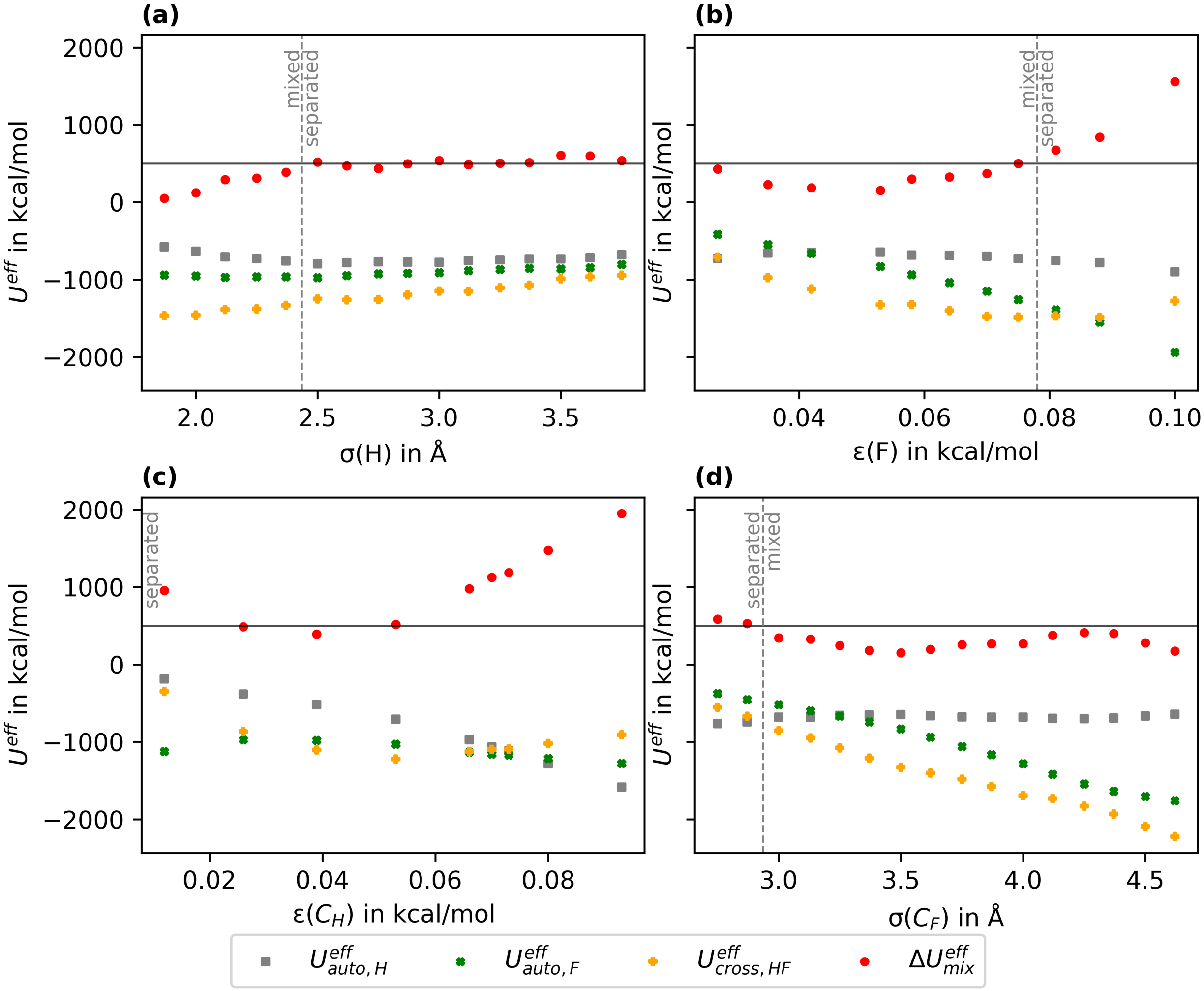

Figure S5: Effective interaction energies $U^{\text{eff}}_{\text{auto,H}}$, $U^{\text{eff}}_{\text{auto,F}}$ and $U^{\text{eff}}_{\text{cross,HF}}$ obtained from Equation 2 and effective energy of mixing $\Delta U^{\text{eff}}_{\text{mix}}$ obtained from Equation 3 as a function of some Lennard-Jones parameters. **a)** variation of $\sigma(H)$ at 220 K, **b)** variation of $\varepsilon(F)$ at 240 K, **c)** variation of $\varepsilon(C_H)$ at 200 K, **d)** variation of $\sigma(C_F)$ at 240 K. The vertical dashed lines indicate the mixed/separated phase transitions reported in the simulation dataset in reference [4]. The horizontal line at 500 kcal/mol acts as a visual aid to correlate $\Delta U^{\text{eff}}_{\text{mix}}$ to these observed transitions.

References

(1) Upterworth, A. L.; Sebastiani, D. Effective Interaction Strength in Simulations of Liquid Mixtures: A Configuration-Dependent Species-Specific Measure of Interaction Enthalpies. *J. Phys. Chem. B* **2025**, *129*, 7818–7825.

(2) Jorgensen, W. L.; Maxwell, D. S.; Tirado-Rives, J. Development and Testing of the OPLS All-Atom Force Field on Conformational Energetics and Properties of Organic Liquids. *J. Am. Chem. Soc.* **1996**, *118*, 11225–11236.

(3) Watkins, E. K.; Jorgensen, W. L. Perfluoroalkanes: Conformational Analysis and Liquid-State Properties from ab Initio and Monte Carlo Calculations. *J. Phys. Chem. A* **2001**, *105*, 4118–4125.

(4) Upterworth, A. L.; Steinkopf, T. E.; Sebastiani, D. Molecular Origins of Philicity: How Atomic Interactions Determine Miscibility and Diffusivity. *ChemPhysChem* **2026**, *27*, e202500875.
